# Trends in antibiotic dispensing for children in Belgian ambulatory care: time series analysis before, during, and after the COVID-19 pandemic

**DOI:** 10.1093/jacamr/dlaf135

**Published:** 2025-07-29

**Authors:** Hannelore Dillen, Axelle Van de Velde, Chloë Withofs, Laure Wynants, Jan Y Verbakel

**Affiliations:** Leuven Unit for Health Technology Assessment Research (LUHTAR), Department of Public Health and Primary Care, KU Leuven, 7 Kapucijnenvoer, Leuven 3000, Belgium; Academic Centre for General Practice, Department of Public Health and Primary Care, KU Leuven, 7 Kapucijnenvoer, Leuven 3000, Belgium; Faculty of Medicine, KU Leuven, 49 Herestraat, Leuven 3000, Belgium; Faculty of Medicine, KU Leuven, 49 Herestraat, Leuven 3000, Belgium; Leuven Unit for Health Technology Assessment Research (LUHTAR), Department of Public Health and Primary Care, KU Leuven, 7 Kapucijnenvoer, Leuven 3000, Belgium; Department of Epidemiology, CAPHRI Care and Public Health Research Institute, Maastricht University, 1 Peter Debyeplein, Maastricht 6229 HA, Netherlands; Department of Development and Regeneration, KU Leuven, 49 Herestraat, Leuven 3000, Belgium; Leuven Unit for Health Technology Assessment Research (LUHTAR), Department of Public Health and Primary Care, KU Leuven, 7 Kapucijnenvoer, Leuven 3000, Belgium; Academic Centre for General Practice, Department of Public Health and Primary Care, KU Leuven, 7 Kapucijnenvoer, Leuven 3000, Belgium

## Abstract

**Objective:**

The aim of this study is to analyse trends in paediatric antibiotic use in Belgian ambulatory care across three COVID-19 pandemic-related periods.

**Methods:**

We conducted a retrospective time series analysis using autoregressive integrated moving average modelling. The analysis is based on anonymized pharmacy dispensing data for antibiotics delivered to Belgian children aged 0–12 years, retrieved from Farmanet for the period from 2014 until 2023. The outcome measures were the number of packages, expenditures and DDDs. Outcomes were analysed for all antibiotics collectively and for subgroups based on patient characteristics, prescriber specialty, geographic region and antibiotic characteristics.

**Results:**

Antibiotic use among children in Belgian ambulatory care sharply declined during the COVID-19 pandemic (−42.7%), followed by a gradual return to pre-pandemic levels (+66.9%), which was primarily driven by prescriptions of antibiotics commonly used for respiratory tract infections. The initial reduction exceeded expected seasonal variations. The largest decreases during the pandemic and subsequent increases were observed among children aged 7–12 years, those with standard reimbursement, in prescriptions by general practitioners and in rural areas of Flanders and the Walloon region.

**Conclusions:**

The COVID-19 pandemic significantly disrupted paediatric antibiotic prescribing patterns in Belgian ambulatory care. These findings highlight the importance of sustained antimicrobial stewardship efforts, not only in routine healthcare settings but also during periods of altered care delivery.

## Introduction

Antimicrobial resistance (AMR) is among the leading global health threats, with an estimated 1.27 million deaths attributable to bacterial AMR in 2019, rivalling the combined mortality of influenza, tuberculosis and HIV/AIDS.^1^ Overuse and misuse of antibiotics are key drivers of AMR.^2^ Children are frequently prescribed antibiotics despite guidelines advising against their use, often with non-first-line agents or incorrect doses.^3^ Factors driving these patterns include diagnostic uncertainty, parental expectations, time constraints and financial incentives.^4,5^

The COVID-19 pandemic drastically altered routine healthcare delivery. Public health measures such as school closure, teleworking, travel restrictions and hygiene protocols reduced infection transmission but also limited in-person healthcare access.^6,7^ Remote consultations increased diagnostic uncertainty, further complicating antibiotic prescribing.^8–11^ In Belgium^12^ and several other countries,^8,9,13,14^ consultations for respiratory tract infections (RTIs) declined significantly during the pandemic, with reductions partially offset by remote consultations.^10,11^ At the beginning of the pandemic, Belgian ambulatory care physicians felt overwhelmed with the increased administrative workload^11^ and with the constantly changing information related to personal protective equipment, telephone triage and practical guidance on managing patients with COVID-19 symptoms and maintaining or restarting routine care.^10^

Understanding the impact of these shifts on paediatric antibiotic use can inform future antimicrobial stewardship efforts, particularly in supporting appropriate antibiotic consumption during and after pandemics. This study aims to analyse trends in antibiotic use in ambulatory care across three pandemic-related periods in the densely populated country of Belgium. We assess overall trends and conduct subgroup analyses by patient characteristics, prescriber specialties, geographic regions and antibiotic characteristics.

## Patients and methods

This study meets the REporting of studies Conducted using Observational Routinely collected health Data criteria (RECORD; Supplementary data).^15^

### Study design and setting

We conducted a retrospective analysis of anonymized pharmacy dispensing data in Belgium. The first COVID-19 preventative measures were implemented on 13 March 2020,^16^ with subsequent adjustments, until restrictions were lifted in March 2022.^16^

### Data sources

Pharmacy dispensing data for systemic antibiotics [classified according to the Anatomical Therapeutic Chemical (ATC) classification system, J01]^17^ were received from Farmanet, a Belgian database covering community pharmacy dispensation reimbursed by the compulsory health insurance.^18^ We requested weekly dispensing data (Monday to Sunday) for children aged 0 up to and including 12 years from 2014 to 2023. Age was calculated as the delivery year of the antibiotic minus the birth year of the child. Population data of children aged 0–12 years were sourced from the Belgian statistical office (Statbel) for normalization.^19^ Antibiotics for topical (ATC D06A) and ophthalmic (ATC S01A) use were excluded.

### Variables

Dispensing data included the number of packages, expenditures covered by the National Institute for Health and Disability Insurance (NIHDI) and DDDs. Subgroup analyses were stratified by the following:

- Patient characteristics: Age, sex and reimbursement type. N.B.: Some Belgian inhabitants are eligible for increased reimbursement based on their income and social situation.
- Prescriber specialty: General practitioners (GPs), specialists and dentists.
- Geographic region: Flanders, Walloon region, Brussels Capital Region and rurality (city, town/suburb, rural, based on the European Commission's DEGURBA classification).^20^ N.B.: Belgium is a densely populated country, averaging 385 inhabitants per km^2^ in 2025, with substantial regional variation: 504 in Flanders, 219 in the Walloon Region and 7732 in Brussels Capital Region.^21^
- Antibiotic characteristics: ATC-3 class, compound and spectrum of antibiotic activity.

The study period was divided into pre-COVID-19 (1 January 2014 to 15 March 2020; 324 weeks), during COVID-19 (16 March 2020 to 13 March 2022; 104 weeks) and post-COVID-19 (14 March 2022 to 31 December 2023; 94 weeks).

### Data analysis

For all antibiotics collectively and for each subgroup, the number of dispensed antibiotic packages, expenditures and DDDs were calculated by year and by time period related to COVID-19 (standardized by the number of weeks). Descriptive statistics included adjusted antibiotic use (i.e. per 1000 children), proportions and absolute/relative changes.

Trends in antibiotic dispensing were analysed using autoregressive integrated moving average (ARIMA) models.^22^ We applied the Box–Jenkins method and an automated algorithm (i.e. the R function auto.arima) to determine the parameters of the ARIMA model.^22^ We performed the Dickey–Fuller test to identify stationarity and the combined test to assess seasonality. We constructed (partial) autocorrelation functions [(P)ACFs] of differenced time series data to visually inspect autocorrelation. The final model includes the following external regressors (i.e. predictors of the outcome): (i) a binary variable for the during COVID-19 period; (ii) a binary variable for the post-COVID-19 period; (iii) the proportion of the outcome attributed to girls; and (iv) the proportion attributed to young children (aged 0–6 years). We diagnosed all models based on information criteria (e.g. AIC) and the Ljung–Box test for autocorrelation remaining in the residuals. We developed two types of forecasts using the final ARIMA models: one based on the complete dataset and another to compare observed with expected outcomes under the assumption of no COVID-19 pandemic (i.e. forecast based on the outcomes before the COVID-19 pandemic). We performed a sensitivity analysis with only the antibiotics primarily used for the treatment of RTIs in children. Only complete cases were considered for the analyses; missing data were not imputed.

Descriptive statistics were performed using Microsoft Excel (version 2408). Data visualization and ARIMA modelling were conducted in R (version 4.1.3), using the ggplot2 and forecast packages, respectively. The full R code, as well as (P)ACF, decomposition and residual plots are stored in a data repository.^23^

## Results

From 2014 to 2023, 11.9 million antibiotic packages were dispensed to children in Belgian community pharmacies. Packages were excluded due to missing data: 0.35% for geographic region, 0.046% for prescriber specialty and 0.019% for reimbursement type.

### General trends from 2014 until 2023

**Figure 1.**
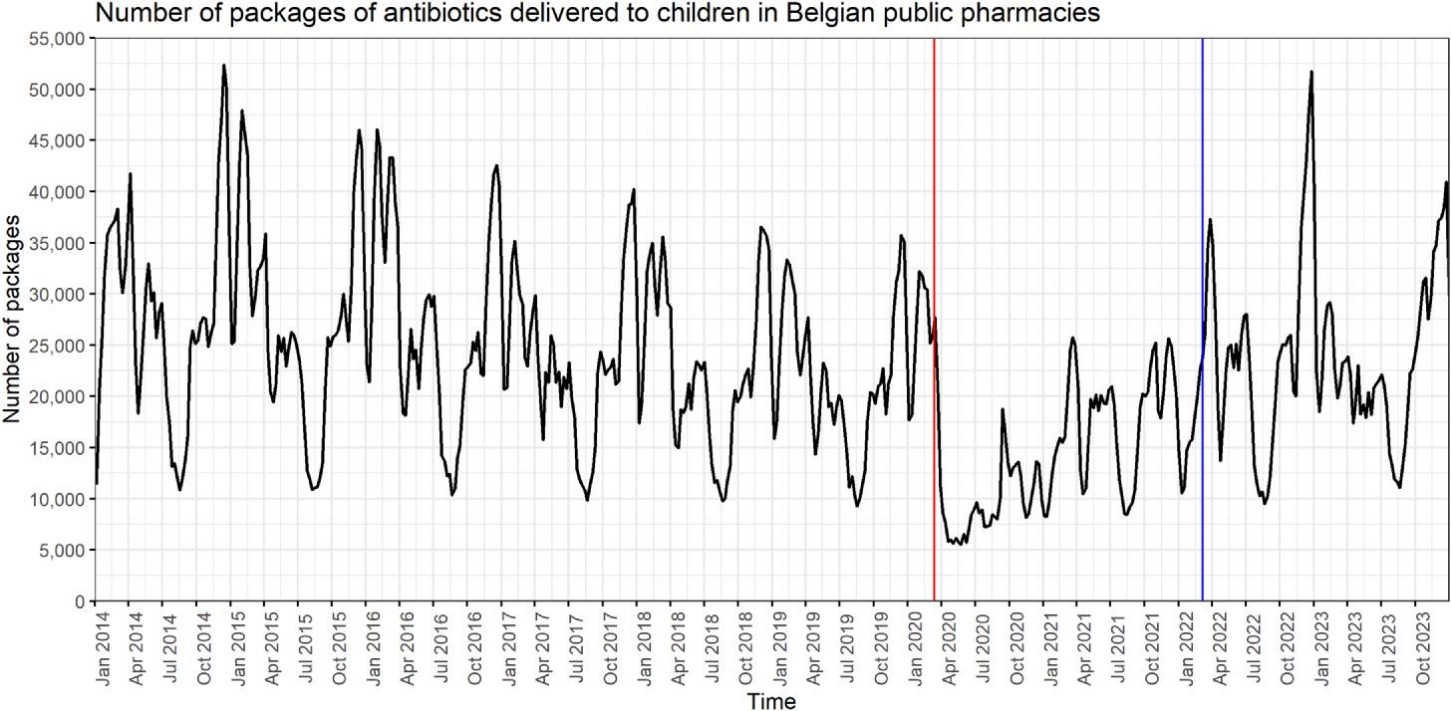
Number of packages of antibiotics delivered to children in Belgian public pharmacies, 2014–23. The vertical lines represent the start of the COVID-19 pandemic (i.e. the week of 16 March 2020) and the start of the post-COVID-19 period (i.e. the week of 14 March 2022), respectively.

In 2023, 744 packages of antibiotics per 1000 inhabitants were delivered in Belgian community pharmacies to children (Table S1, available as Supplementary data at *JAC-AMR* Online). Between 2014 and 2023, antibiotic use decreased by 17.3%, expenditures by 30.2% and DDDs by 3.58% (Figure 1; Table S2 and Figure S1), while DDDs per package increased by 16.5% (Table S3). During the pandemic, antibiotic use dropped by 42.7% compared to pre-COVID-19 levels (i.e. step change) but rebounded post-pandemic, increasing by 66.9% (i.e. pulse function) (Table S4 and Figures S1–S3). The rebound was most pronounced for RTI-related antibiotics (+72.1%) (Table S5).

All time series exhibited autocorrelation and seasonality. Differencing of the series succeeded in rendering them stationary. The COVID-19 period had a significant negative association with all outcomes, with the following weekly estimates: −6106 number of packages (95% CI −10 817 to −1395), −45 657 euros healthcare expenditures (95% CI −74 504 to −16 809) and −25 216 DDDs (95% CI −46 958 to −3473) (Table S6), which was also illustrated in the forecast plot of observed compared to expected outcomes (Figure S4 and Table S7). The percentage of the outcome attributed to young children had a significant negative association with all outcomes, while the period after COVID-19 and the percentage of the outcome attributed to girls did not (Table S6). Although the residuals of all models did not show any obvious pattern, the results of the autocorrelation tests were statistically significant based on the Ljung–Box test (Table S6). In the sensitivity analysis, ARIMA models showed similar results (Table S8). Antibiotic use and expenditures are expected to show a decreasing trend between 2024 and 2026 (Figure S5).

### Patient characteristics

During the pandemic, antibiotic use decreased comparably for both sexes and subsequently increased similarly after the pandemic (Table S9 and Figures S6 and S7). In 2023, 2–6-year-olds accounted for almost half (49.2%) of antibiotic use, followed by 7–12-year-olds (34.0%) and 0–1-year-olds (16.8%). Antibiotic use decreased across all age groups during COVID-19, with the highest decrease among 7–12-year-olds (Table S9 and Figures S8 and S9). After the pandemic, use increased across all age groups, with the highest increase across 7–12-year-olds and a slightly greater relative increase in boys within this age group (Figure S10). Antibiotic use decreased for children with both standard and increased reimbursement during the pandemic and increased thereafter, with the greatest reductions and subsequent increases occurring among children with a standard reimbursement (Table S10 and Figures S11 and S12).

### Specialty of the prescriber

**Figure 2.**
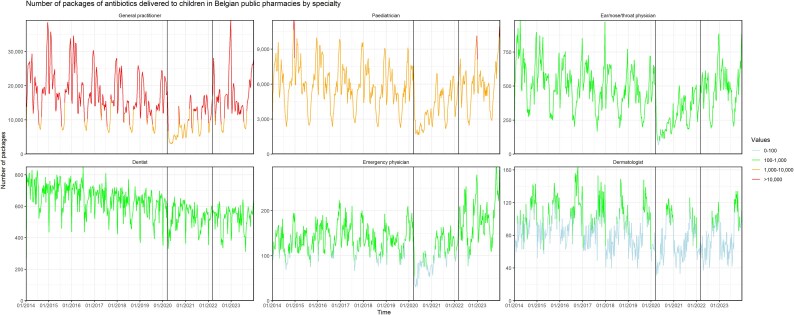
Number of packages of antibiotics delivered to children in Belgian public pharmacies, 2014–23, by specialty. The vertical lines represent the start of the COVID-19 pandemic (i.e. the week of 16 March 2020) and the start of the post-COVID-19 period (i.e. the week of 14 March 2022), respectively. The *y*-axes are on different scales.

GPs accounted for 67.1% of ambulatory paediatric antibiotic prescriptions in 2023, followed by paediatricians (25.3%), dentists (2.25%) and ear/nose/throat physicians (2.17%). During the pandemic, all specialties reduced antibiotic prescribing, with the largest reductions observed among GPs (Figure 2; Table S11 and Figures S13–S16). Post-pandemic, GPs and specialists increased prescribing, while dentists’ prescribing remained stable.

### Geographic regions

Antibiotic use decreased across all regions and rurality categories during the pandemic (Table S12 and Figures S17–S21). Post-pandemic, the highest increases were observed in the Walloon region and rural areas in Flanders.

### Antibiotic classes and compounds

Penicillins accounted for 77.6% of paediatric antibiotic use in 2023 and macrolides, lincosamides and streptogramins contributed 19.3%. Amoxicillin (60.1%), amoxicillin/clavulanate (16.6%) and azithromycin (13.6%) together accounted for 90.3% of total use. Amoxicillin accounted for 78.7% of the combined total of amoxicillin and amoxicillin/clavulanate packages (Table S13).

**Figure 3.**
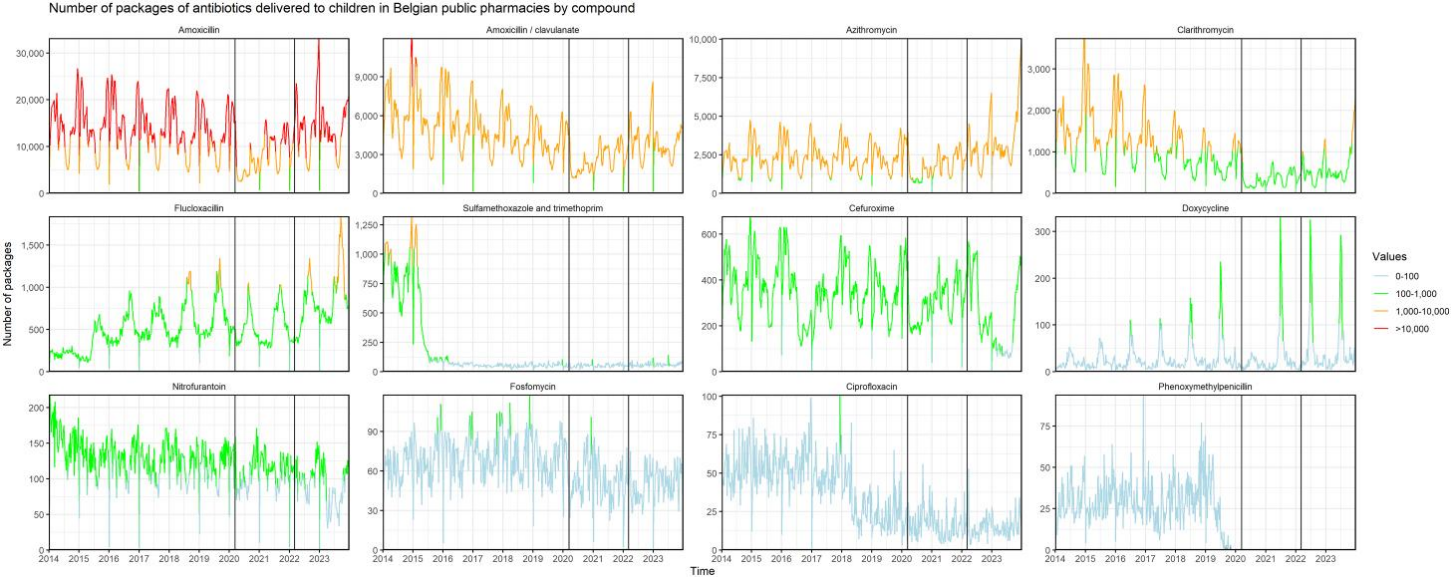
Number of packages of antibiotics delivered to children in Belgian public pharmacies, 2014–23, by compound. The vertical lines represent the start of the COVID-19 pandemic (i.e. the week of 16 March 2020) and the start of the post-COVID-19 period (i.e. the week of 14 March 2022), respectively. The *y*-axes are on different scales.

During the pandemic, antibiotic use decreased for all classes and remained stable for tetracyclines (Table S14 and Figures S22 and S23). A small increase was observed for the use of doxycycline and the narrow-spectrum antibiotic flucloxacillin (Figure 3; Tables S15 and S16 and Figures S24–S26). After the pandemic, antibiotic use continued to reduce for other antibacterials (i.e. J01X, e.g. nitrofurantoin and fosfomycin), aminoglycoside antibacterials, other beta-lactam antibacterials (i.e. J01D, e.g. cefuroxime) and quinolones (e.g. ciprofloxacin). An increase was observed for macrolides, lincosamides and streptogramins (e.g. azithromycin, clarithromycin), penicillins (e.g. amoxicillin, flucloxacillin), amphenicols, tetracyclines (e.g. doxycycline) and sulphonamides and trimethoprim.

## Discussion

### Main findings

Antibiotic use among children in Belgian ambulatory care sharply declined during the COVID-19 pandemic, exceeding expected seasonal variations, and was followed by a gradual return to pre-pandemic levels. This trend was primarily driven by changes in prescriptions of antibiotics commonly used for RTIs. Conversely, antibiotics mainly prescribed for urinary tract and skin infections were less affected. For example, flucloxacillin use did not decrease significantly, while cefuroxime and nitrofurantoin continued a sustained downward trend without rebounding post-pandemic. These shifts likely reflect a reduction in RTIs and changes in patient consultation patterns rather than fundamental changes in prescriber behaviour. The proportion between amoxicillin and amoxicillin/clavulanate approximated the national target of 80%.^24^ The largest decreases during the pandemic and subsequent increases were observed among 7–12-year-olds, children with standard reimbursement, in prescriptions by GPs and in rural areas of Flanders and the Walloon region. In contrast to younger children, who may have continued attending day care or preschool during the pandemic, children aged 7–12 years could be more affected by prolonged primary school closures. As a result, their exposure to infections might have dropped more sharply, leading to a steeper decline in antibiotic use, followed by a more pronounced rebound once schools reopened.

### Strengths and limitations

A major strength of this study is the use of ARIMA models, which account for secular trends, seasonality and autocorrelation,^22^ enabling a clear distinction between pandemic-related effects and ongoing trends in outpatient antibiotic use. Additionally, the Farmanet captures comprehensive antibiotic dispensing data, covering approximately 99% of the Belgian population,^25^ allowing for detailed stratified analyses. The study has some limitations. We were unable to assess the appropriateness of antibiotic prescriptions due to the absence of diagnostic information, leaving uncertainty about potential undertreatment, complications or increased hospital referrals. Further, we could not link prescription data to physician visit, limiting insights into the relative contributions of RTI incidence, decreased consultations and shifts to remote healthcare. Besides, given that DDDs are based on adult dosing and may not accurately capture paediatric antibiotic use, especially in younger children, we primarily present data on the number of dispensed packages. However, DDDs were included as a secondary measure to enable comparison with other studies, and we observed an increasing dose per package over time. Other alternatives for DDDs include the number of treatments or prescriptions (e.g. by diagnosis) and the days of therapy (in inpatient settings). Lastly, public pharmacy dispensing data may underestimate overall antibiotic use, excluding hospital prescriptions, leftovers, those obtained without prescription (e.g. abroad, friends and family) or the use of compounds outside of reimbursable clinical scenarios (e.g. quinolones).^25^

### Comparison with existing literature

The observed trends—pre-pandemic decline, sharp reduction during the COVID-19 pandemic and subsequent rebound—align with findings across all age groups in Belgium,^26^ within^27^ and outside Europe.^14,28^ Studies reported similar declines in face-to-face outpatient visits^13,29,30^ and RTI episodes during the pandemic.^8,14^ In Belgium, GP-reported infection rate among children dropped significantly in 2020 and 2021, before rebounding in 2022.^12^ These patterns support the hypothesis that reduced antibiotic use reflects a decrease in RTI incidence and altered health-seeking behaviour, encouraging parents to adopt watchful waiting approaches^31^ and improving self-management skills.^9^

### Implications for clinical practice and further research

This study provides valuable insights for national policymakers into how the COVID-19 pandemic affected antibiotic prescribing. Belgium is among the highest antibiotic consumers in Europe, with a population-weighted mean primary care consumption of 19.1 DDDs per 1000 inhabitants per day (DID) in 2023, exceeding the European average (18.3 DID), as well as neighbouring countries such as the Netherlands (8.8 DID) and Germany (11.7 DID).^27^ The findings of this study underscore the importance of sustained antimicrobial stewardship efforts, as highlighted in Belgium’s One Health-based Action Plan on AMR.^24^ This includes strategies such as infection prevention and control, the rational use of antibiotics, raising awareness and education and conducting audits.^24^ The observed reduction in antibiotic prescribing during the pandemic may represent an opportunity for prescribers to reflect on and refine their prescribing behaviours. Future stewardship initiatives could target reducing unnecessary prescriptions while ensuring patient safety, particularly in light of potential cost-savings (e.g. €4.7 million total reduction during the pandemic—based on the ARIMA model) and the broader economic implications of AMR (annual cost of €24 million in Belgium).^24^ Public health measures to curb COVID-19 also effectively reduced RTI transmission and influenced health-seeking behaviour. These outcomes highlight the potential of similar strategies to support AMR efforts. In particular, in Belgium, school closures were associated with reduced peak hospital occupancy during COVID-19,^32^ consistent with earlier findings that weekends and holidays slow influenza epidemics.^33^ Future epidemic response strategies should therefore incorporate carefully designed school closure policies^32^ alongside infection prevention measures such as mask wearing, hand hygiene and physical distancing.

Future studies should examine antibiotic prescriptions alongside infection episodes, consultation rates, symptom duration, complication rates and hospital referrals to provide a more comprehensive understanding of prescribing behaviours and appropriateness. Evaluating the long-term impact of these trends on AMR is crucial. The pandemic has advanced genomics-based infectious disease surveillance, offering new opportunities for AMR monitoring.^26^ Additionally, qualitative research through interviews with physicians, parents and children could inform customized antimicrobial stewardship programmes by capturing their experiences with antibiotic use during and after the pandemic.

### Conclusions

The COVID-19 pandemic significantly disrupted paediatric antibiotic prescribing patterns in Belgian ambulatory care. The observed reductions and subsequent rebounds underscore the importance of sustained antimicrobial stewardship efforts. These findings provide valuable insights for optimizing antibiotic use and addressing AMR in evolving healthcare contexts.

## Supplementary Material

dlaf135_Supplementary_Data

## Supplementary data

Figures S1–S26 and Tables S1–S16 are available as Supplementary data at *JAC-AMR* Online.

## References

[1] Antimicrobial Resistance Collaborators . Global burden of bacterial antimicrobial resistance in 2019: a systematic analysis. Lancet 2022; 399: 629–55. 10.1016/S0140-6736(21)02724-0 35065702 PMC8841637

[2] McDonagh MS, Peterson K, Winthrop K et al Interventions to reduce inappropriate prescribing of antibiotics for acute respiratory tract infections: summary and update of a systematic review. J Int Med Res 2018; 46: 3337–57. 10.1177/0300060518782519 29962311 PMC6134646

[3] Dillen H, Wouters J, Snijders D et al Factors associated with inappropriateness of antibiotic prescriptions for acutely ill children presenting to ambulatory care in high-income countries: a systematic review and meta-analysis. J Antimicrob Chemother 2024; 79: 498–511. 10.1093/jac/dkad383 38113395 PMC10904728

[4] Rose J, Crosbie M, Stewart A. A qualitative literature review exploring the drivers influencing antibiotic over-prescribing by GPs in primary care and recommendations to reduce unnecessary prescribing. Perspect Public Heal 2021; 141: 19–27. 10.1177/1757913919879183 31633458

[5] Lucas PJ, Cabral C, Hay AD et al A systematic review of parent and clinician views and perceptions that influence prescribing decisions in relation to acute childhood infections in primary care. Scand J Prim Health 2015; 33: 11–20. 10.3109/02813432.2015.1001942 PMC4377734 25716427

[6] van Ballegooijen H, Goossens L, Bruin RH et al Concerns, quality of life, access to care and productivity of the general population during the first 8 weeks of the coronavirus lockdown in Belgium and the Netherlands. BMC Health Serv Res 2021; 21: 227. 10.1186/s12913-021-06240-7 33712010 PMC7953179

[7] Hussain AZ, Paudyal V, Hadi MA. Impact of the COVID-19 pandemic on the prescribing patterns of first-line antibiotics in English primary care: a longitudinal analysis of national prescribing dataset. Antibiotics 2021; 10: 591. 10.3390/antibiotics10050591 34067546 PMC8156075

[8] van de Pol AC, Boeijen JA, Venekamp RA-O et al Impact of the COVID-19 pandemic on antibiotic prescribing for common infections in the Netherlands: a primary care-based observational cohort study. Antibiotics 2021; 10: 196. 10.3390/antibiotics10020196 33670657 PMC7922191

[9] Borek AJ, Maitland K, McLeod M et al Impact of the COVID-19 pandemic on community antibiotic prescribing and stewardship: a qualitative interview study with general practitioners in England. Antibiotics 2021; 10: 1531. 10.3390/antibiotics10121531 34943743 PMC8698307

[10] Wanat M, Hoste M, Gobat N et al Transformation of primary care during the COVID-19 pandemic: experiences of healthcare professionals in eight European countries. Brit J Gen Pract 2021; 71: e634–42. 10.3399/BJGP.2020.1112 33979303 PMC8274627

[11] Verhoeven V, Tsakitzidis G, Philips H et al Impact of the COVID-19 pandemic on the core functions of primary care: will the cure be worse than the disease? A qualitative interview study in Flemish GPs. BMJ Open 2020; 10: e039674. 10.1136/bmjopen-2020-039674 PMC7306272 32554730

[12] Burvenich R, De Boodt S, Lowie L et al Temporal trends in antibiotic prescribing and serious and nonserious infections in children presenting to general practice: a registry-based longitudinal cohort study of 162 507 individuals. J Antimicrob Chemother 2024; 79: 1397–406. 10.1093/jac/dkae117 38714502

[13] Kitano T, Brown KA, Daneman N et al The impact of COVID-19 on outpatient antibiotic prescriptions in Ontario, Canada; an interrupted time series analysis. Open Forum Infect Dis 2021; 8: ofab533. 10.1093/ofid/ofab533 34805442 PMC8601042

[14] Dutcher L, Li Y, Lee G et al COVID-19 and antibiotic prescribing in pediatric primary care. Pediatrics 2022; 149: e2021053079. 10.1542/peds.2021-053079 35102416 PMC9825803

[15] Benchimol EI, Smeeth L, Guttmann A et al The REporting of studies Conducted using Observational Routinely-collected health Data (RECORD) statement. PLOS Med 2015; 12: e1001885. 10.1371/journal.pmed.1001885 26440803 PMC4595218

[16] Nationaal Crisiscentrum . Coronavirus: de antwoorden op al je vragen. 2021. https://crisiscentrum.be/nl/newsroom/coronavirus-de-antwoorden-op-al-je-vragen.

[17] Norwegian Institute of Public Health . ATC—Structure and principles. 2022. https://atcddd.fhi.no/atc/structure_and_principles/.

[18] RIZIV . Statistieken over geneesmiddelen afgeleverd in openbare apotheken (Farmanet). https://www.riziv.fgov.be/nl/statistieken/statistieken-van-geneesmiddelen/statistieken-over-geneesmiddelen-afgeleverd-in-openbare-apotheken-farmanet.

[19] Belgische Federale Overheidsdiensten . STATBEL—België in cijfers. 2017. https://statbel.fgov.be/nl/themas/bevolking/structuur-van-de-bevolking#figures.

[20] European Union . Degree of Urbanisation (DEGURBA). 2017. https://www.eea.europa.eu/data-and-maps/data/external/degree-of-urbanisation-degurba.

[21] North Gate II & III—INS (STATBEL—Statistics Belgium) . Population Density. 2025. http://data.europa.eu/88u/dataset/c37c003f8cc1737d21f3f4bdb84eacfc7b407320.

[22] Schaffer AL, Dobbins TA, Pearson S-A. Interrupted time series analysis using autoregressive integrated moving average (ARIMA) models: a guide for evaluating large-scale health interventions. BMC Med Res Methodol 2021; 21: 58. 10.1186/s12874-021-01235-8 33752604 PMC7986567

[23] Dillen H . Trends in Antibiotic Dispensing for Children in Belgian Ambulatory Care: Time Series Analysis Before, During, and After the COVID-19 Pandemic. KU Leuven RDR, V1, 2025. 10.48804/DMZ3DN PMC12305424 40735513

[24] FOD Volksgezondheid, Veiligheid van de Voedselketen en Leefmilieu . Belgisch Nationaal Actieplan “One Health” voor de bestrijding van antimicrobiële resistentie (AMR) 2020–2024. 2021.

[25] Damian E, Bonacini L, Kelly M et al Monitoring community antibiotic consumption in Belgium: reimbursement versus retail data (2013–22). J Antimicrob Chemother 2025; 80: 138–46. 10.1093/jac/dkae384 39450851 PMC11695903

[26] *BELMAP 2024: One Health Report of Antimicrobial Consumption and Resistance in Belgium*. Brussels, Belgium, 2024. 10.25608/v1x6-1e19

[27] European Centre for Disease Prevention and Control . *Antimicrobial Consumption in the EU/EEA (ESAC—Net)—Annual Epidemiological Report 2023*. ECDC, 2024.

[28] Nandi A, Pecetta S, Bloom DE. Global antibiotic use during the COVID-19 pandemic: analysis of pharmaceutical sales data from 71 countries, 2020–2022. EClinicalMedicine 2023; 57: 101848. 10.1016/j.eclinm.2023.101848 36776504 PMC9900305

[29] Colliers A, De Man J, Adriaenssens N et al Antibiotic prescribing trends in Belgian out-of-hours primary care during the COVID-19 pandemic: observational study using routinely collected health data. Antibiotics 2021; 10: 1488. 10.3390/antibiotics10121488 34943701 PMC8698421

[30] Armitage R, Nellums LB. Antibiotic prescribing in general practice during COVID-19. Lancet Infect Dis 2021; 21: e144. 10.1016/S1473-3099(20)30917-8 33275941 PMC9761101

[31] Chua KP, Volerman A, Conti RM. Prescription drug dispensing to US children during the COVID-19 pandemic. Pediatrics 2021; 148: e2021049972. 10.1542/peds.2021-049972 34285080 PMC8344340

[32] Ragonnet R, Hughes AE, Shipman DS et al Estimating the impact of school closures on the COVID-19 dynamics in 74 countries: a modelling analysis. PLOS Med 2025; 22: e1004512. 10.1371/journal.pmed.1004512 39836691 PMC11793732

[33] De Luca G, Kerckhove KV, Coletti P et al The impact of regular school closure on seasonal influenza epidemics: a data-driven spatial transmission model for Belgium. BMC Infect Dis 2018; 18: 29. 10.1186/s12879-017-2934-3 29321005 PMC5764028

