# Supplementary material for: Trends in antibiotic dispensing for children in Belgian ambulatory care: time series analysis before, during, and after the COVID-19 pandemic
Source: JAC Antimicrob Resist. 2025 Jul 29;7(4):dlaf135. doi: 10.1093/jacamr/dlaf135 (PMC12305424; doi:10.1093/jacamr/dlaf135)
Supplement: dlaf135_Supplementary_Data [file dlaf135_supplementary_data.pdf]

SUPPLEMENTARY MATERIAL

Trends in antibiotic dispensing for children in Belgian ambulatory care: Time series analysis before, during, and after the COVID-19 pandemic

TABLE OF CONTENTS

REPORTING OF STUDIES CONDUCTED USING OBSERVATIONAL ROUTINELY COLLECTED HEALTH DATA CRITERIA (RECORD) 4

GENERAL TRENDS FROM 2014 UNTIL 2023 8

Table S1. Number of packages, healthcare expenditures and Defined Daily Doses (DDDs) per 1000 inhabitants per year. 8

Table S2. Relative change of number of packages, healthcare expenditures and Defined Daily Doses (DDDs) per year. 8

Table S3. Proportion of Defined Daily Doses (DDDs) compared to number of packages, per year. 8

Table S4. Relative change of number of packages, healthcare expenditures and Defined Daily Doses (DDDs) per period (standardised per week). 8

Table S5. Relative change of number of packages, healthcare expenditures and Defined Daily Doses (DDDs) per period (standardised per week), sensitivity analysis*. 8

Figure S1. Line chart of antibiotics delivered to children in Belgian public pharmacies, 2014 to 2023, expressed as healthcare expenditures (a) and Defined Daily Doses (DDDs) (b). ...10

Figure S2. Line chart of antibiotics delivered to children in Belgian public pharmacies by year and week, expressed as number of packages (a), healthcare expenditures (b), and Defined Daily Doses (DDDs) (c). 13

Figure S3. Heatmap of antibiotics delivered to children in Belgian public pharmacies by year and month, expressed as number of packages (a), healthcare expenditures (b), and Defined Daily Doses (DDDs) (c). 15

Table S6. Results from ARIMA models........16

Figure S4. Antibiotics delivered to children in Belgian public pharmacies: observed and expected outcomes of the number of packages (a), healthcare expenditures (b), and Defined Daily Doses (DDDs) (c). 20

Table S7. Accuracy measures from ARIMA models based on the outcomes before the COVID-19 pandemic. 21

Table S8. Results from ARIMA models, sensitivity analysis*. 22

Figure S5. Forecast of antibiotics delivered to children in Belgian public pharmacies, expressed as number of packages (a), healthcare expenditures (b), and Defined Daily Doses (DDDs) (c). 26

PATIENT CHARACTERISTICS........27

Table S9. Relative change of number of packages, healthcare expenditures and Defined Daily Doses (DDDs) by age group and sex, per period (standardised per week). 27

Table S10. Relative change of number of packages, healthcare expenditures and Defined Daily Doses (DDDs) by reimbursement type, per period (standardised per week). 27

Figure S6. Line chart of antibiotics delivered to children in Belgian public pharmacies by sex, expressed as number of packages (a), healthcare expenditures (b), and Defined Daily Doses (DDDs) (c). 30

Figure S7. Bar chart of antibiotics delivered to children in Belgian public pharmacies by sex, expressed as number of packages (a), healthcare expenditures (b), and Defined Daily Doses (DDDs) (c). 33

Figure S8. Line chart of antibiotics delivered to children in Belgian public pharmacies by age group, expressed as number of packages (a), healthcare expenditures (b), and Defined Daily Doses (DDDs) (c). 36

Figure S9. Bar chart of antibiotics delivered to children in Belgian public pharmacies by age group, expressed as number of packages (a), healthcare expenditures (b), and Defined Daily Doses (DDDs) (c). 39

Figure S10. Line chart of antibiotics delivered to children in Belgian public pharmacies by age and sex, expressed as number of packages (a), healthcare expenditures (b), and Defined Daily Doses (DDDs) (c). 42

Figure S11. Line chart of antibiotics delivered to children in Belgian public pharmacies by reimbursement type, expressed as number of packages (a), healthcare expenditures (b), and Defined Daily Doses (DDDs) (c). 45

Figure S12. Bar chart of antibiotics delivered to children in Belgian public pharmacies by reimbursement type, expressed as number of packages (a), healthcare expenditures (b), and Defined Daily Doses (DDDs) (c). 48

SPECIALTY OF THE PRESCRIBER....49

Table S11. Relative change of number of packages, healthcare expenditures and Defined Daily Doses (DDDs) by specialty, per period (standardised per week). 49

Figure S13. Line chart of antibiotics delivered to children in Belgian public pharmacies by specialty group, expressed as number of packages (a), healthcare expenditures (b), and Defined Daily Doses (DDDs) (c). 52

Figure S14. Bar chart of antibiotics delivered to children in Belgian public pharmacies by specialty group, expressed as number of packages (a), healthcare expenditures (b), and Defined Daily Doses (DDDs) (c). 55

Figure S15. Line chart of antibiotics delivered to children in Belgian public pharmacies by specialty, expressed as healthcare expenditures (a) and Defined Daily Doses (DDDs) (b)...57

Figure S16. Bar chart of antibiotics delivered to children in Belgian public pharmacies by specialty, expressed as number of packages (a), healthcare expenditures (b), and Defined Daily Doses (DDDs) (c). 60

GEOGRAPHIC REGIONS....61

Table S12. Relative change of number of packages, healthcare expenditures and Defined Daily Doses (DDDs) by geographic region, per period (standardised per week). 61

Figure S17. Line chart of antibiotics delivered to children in Belgian public pharmacies by region, expressed as number of packages (a), healthcare expenditures (b), and Defined Daily Doses (DDDs) (c). 64

Figure S18. Bar chart of antibiotics delivered to children in Belgian public pharmacies by region, expressed as number of packages (a), healthcare expenditures (b), and Defined Daily Doses (DDDs) (c). 67

Figure S19. Line chart of antibiotics delivered to children in Belgian public pharmacies by rurality category, expressed as number of packages (a), healthcare expenditures (b), and Defined Daily Doses (DDDs) (c). 70

Figure S20. Bar chart of antibiotics delivered to children in Belgian public pharmacies by rurality category, expressed as number of packages (a), healthcare expenditures (b), and Defined Daily Doses (DDDs) (c). 73

Figure S21. Line chart of antibiotics delivered to children in Belgian public pharmacies by region and rurality category, expressed as number of packages (a), healthcare expenditures (b), and Defined Daily Doses (DDDs) (c). 76

ANTIBIOTIC CLASSES AND COMPOUNDS 77

Table S13. Proportion of Amoxicillin packages compared to the sum of Amoxicillin and Amoxicillin / clavulanate packages, per year. 77

Table S14. Relative change of number of packages, healthcare expenditures and Defined Daily Doses (DDDs) by ATC-3 class, per period (standardised per week). 77

Table S15. Relative change of number of packages, healthcare expenditures and Defined Daily Doses (DDDs) by compound, per period (standardised per week). 77

Table S16. Relative change of number of packages, healthcare expenditures and Defined Daily Doses (DDDs) by spectrum of antibiotic activity, per period (standardised per week). . 78

Figure S22. Line chart of antibiotics delivered to children in Belgian public pharmacies by ATC-3 class, expressed as number of packages (a), healthcare expenditures (b), and Defined Daily Doses (DDDs) (c). 81

Figure S23. Bar chart of antibiotics delivered to children in Belgian public pharmacies by ATC-3 class, expressed as number of packages (a), healthcare expenditures (b), and Defined Daily Doses (DDDs) (c). 84

Figure S24. Line chart of antibiotics delivered to children in Belgian public pharmacies by compound, expressed as healthcare expenditures (a) and Defined Daily Doses (DDDs) (b). 86

Figure S25. Bar chart of antibiotics delivered to children in Belgian public pharmacies by compound, expressed as number of packages (a), healthcare expenditures (b), and Defined Daily Doses (DDDs) (c). 89

Figure S26. Line chart of antibiotics delivered to children in Belgian public pharmacies by spectrum of antibiotic activity, expressed as number of packages (a), healthcare expenditures (b), and Defined Daily Doses (DDDs) (c). 92

REPORTING OF STUDIES CONDUCTED USING OBSERVATIONAL ROUTINELY COLLECTED HEALTH DATA CRITERIA (RECORD)

	Item number	RECORD items	Location in manuscript where items are reported
Title and abstract			
	1	1.1: The type of data used should be specified in the title or abstract. When possible, the name of the databases used should be included.<br>1.2: If applicable, the geographic region and timeframe within which the study took place should be reported in the title or abstract.<br>1.3: If linkage between databases was conducted for the study, this should be clearly stated in the title or abstract.	1.1 Line 2, 33-35<br>1.2 Line 2-3, 34-35<br>1.3 Not applicable
Introduction			
Background and rationale	2	Explain the scientific background and rationale for the investigation being reported.	Line 83-103
Objectives	3	State specific objectives, including any prespecified hypotheses.	Line 103-106
Methods			
Study Design	4	Present key elements of study design early in the paper.	Line 112
Setting	5	Describe the setting, locations, and relevant dates, including periods of recruitment, exposure, follow-up, and data collection.	Line 113-114, 119-120
Participants	6	6.1: The methods of study population selection (such as codes or algorithms used to identify subjects) should be listed in detail. If this is not possible, an explanation should be provided.<br>6.2: Any validation studies of the codes or algorithms used to select the population should be referenced. If validation was conducted for this study and not published elsewhere, detailed methods and results should be provided.<br>6.3: If the study involved linkage of databases, consider use of a flow diagram or other graphical display to demonstrate the data linkage process, including the number of individuals with linked data at each stage.	6.1 Line 116-121<br>6.2 Not applicable<br>6.3 Not applicable
Variables	7	A complete list of codes and algorithms used to classify exposures, outcomes, confounders, and effect modifiers should be provided. If these cannot be reported, an explanation should be provided.	Line 124-139

Data sources / measurement	8	For each variable of interest, give sources of data and details of methods of assessment (measurement).<br>Describe comparability of assessment methods if there is more than one group.	Line 116-119, 121-122
Bias	9	Describe any efforts to address potential sources of bias.	Line 145-150, 153-155
Study size	10	Explain how the study size was arrived at.	Line 117-119
Quantitative variables	11	Explain how quantitative variables were handled in the analyses. If applicable, describe which groupings were chosen, and why.	Line 141-164
Statistical methods	12	12.1 Describe all statistical methods, including those used to control for confounding.<br>12.2 Describe any methods used to examine subgroups and interactions.<br>12.3 Explain how missing data were addressed.<br>12.4 Cohort study - If applicable, explain how loss to follow-up was addressed.<br>Case-control study - If applicable, explain how matching of cases and controls was addressed.<br>Cross-sectional study - If applicable, describe analytical methods taking account of sampling strategy.<br>12.5 Describe any sensitivity analyses.	12.1 Line 141-146<br>12.2 Line 127-136<br>12.3 Line 159-160<br>12.4 Not applicable<br>12.5 Line 158-159
Data access and cleaning methods	12	12.6 Authors should describe the extent to which the investigators had access to the database population used to create the study population.<br>12.7 Authors should provide information on the data cleaning methods used in the study.	12.6 Line 117-119<br>12.7 Line 141-143
Linkage	12	12.8 State whether the study included person-level, institutional-level, or other data linkage across two or more databases. The methods of linkage and methods of linkage quality evaluation should be provided.	Not applicable
Results			
Participants	13	Describe in detail the selection of the persons included in the study (i.e., study population selection) including filtering based on data quality, data availability and linkage. The selection of included persons can be described in the text and/or by means of the study flow diagram.	Line 167
Descriptive data	14	14.1 Give characteristics of study participants (e.g., demographic, clinical,	14.1 Line 167<br>14.2 Line 168-169

		social) and information on exposures and potential confounders.<br>14.2 Indicate the number of participants with missing data for each variable of interest.<br>14.3 Cohort study - summarise follow-up time (e.g., average and total amount).	14.3 Not applicable
Outcome data	15	Cohort study - Report numbers of outcome events or summary measures over time.<br>Case-control study - Report numbers in each exposure category, or summary measures of exposure.<br>Cross-sectional study - Report numbers of outcome events or summary measures.	Line 167
Main results	16	16.1 Give unadjusted estimates and, if applicable, confounder-adjusted estimates and their precision (e.g., 95% confidence interval). Make clear which confounders were adjusted for and why they were included.<br>16.2 Report category boundaries when continuous variables were categorized.<br>16.3 If relevant, consider translating estimates of relative risk into absolute risk for a meaningful time period.	16.1 Line 171-191<br>16.2 Not applicable<br>16.3 Not applicable
Other analyses	17	Report other analyses done—e.g., analyses of subgroups and interactions, and sensitivity analyses.	Line 189-228
Discussion			
Key results	18	Summarise key results with reference to study objectives.	Line 231-248
Limitations	19	Discuss the implications of using data that were not created or collected to answer the specific research question(s). Include discussion of misclassification bias, unmeasured confounding, missing data, and changing eligibility over time, as they pertain to the study being reported.	Line 249-267
Interpretation	20	Give a cautious overall interpretation of results considering objectives, limitations, multiplicity of analyses, results from similar studies, and other relevant evidence.	Line 268-276
Generalisability	21	Discuss the generalisability (external validity) of the study results.	Line 277-306
Other Information			
Funding	22	Give the source of funding and the role of the funders for the present study and, if applicable, for the original study on which the present article is based.	Line 336-337

Accessibility of protocol, raw data, and programming code	23	Authors should provide information on how to access any supplemental information such as the study protocol, raw data, or programming code.	Line 163-164

GENERAL TRENDS FROM 2014 UNTIL 2023

Table S1. Number of packages, healthcare expenditures and Defined Daily Doses (DDDs) per 1000 inhabitants per year.

Year	Number of packages	Healthcare expenditures	DDDs
2014	888	6,813	3,664
2015	854	6,514	3,605
2016	846	6,400	3,643
2017	730	4,976	3,216
2018	708	4,501	3,168
2019	671	4,050	3,059
2020	416	2,613	1,937
2021	550	3,396	2,527
2022	737	4,516	3,444
2023	744	4,820	3,579

Table S2. Relative change of number of packages, healthcare expenditures and Defined Daily Doses (DDDs) per year.

Year	Number of packages	Healthcare expenditures	DDDs
2014-2015	- 3.18%	- 3.77%	- 1.00%
2015-2016	- 0.48%	- 1.28%	+ 1.55%
2016-2017	- 13.6%	- 22.1%	- 11.6%
2017-2018	- 3.06%	- 9.58%	- 1.53%
2018-2019	- 5.33%	- 10.2%	- 3.67%
2019-2020	- 38.6%	- 35.9%	- 37.1%
2020-2021	+ 31.8%	+ 29.3%	+ 29.8%
2021-2022	+ 33.9%	+ 33.0%	+ 36.4%
2022-2023	- 0.13%	+ 5.57%	+ 2.79%

Table S3. Proportion of Defined Daily Doses (DDDs) compared to number of packages, per year.

Year	DDDs divided by number of packages
2014	4.13
2015	4.22
2016	4.31
2017	4.41
2018	4.48
2019	4.56
2020	4.66
2021	4.59
2022	4.67
2023	4.81

Table S4. Relative change of number of packages, healthcare expenditures and Defined Daily Doses (DDDs) per period (standardised per week).

Period	Number of packages	Healthcare expenditures	DDDs
During vs before COVID	- 42.7%	- 49.1%	- 39.5%
After vs during COVID	+ 66.9%	+ 68.8%	+ 73.8%

Table S5. Relative change of number of packages, healthcare expenditures and Defined Daily Doses (DDDs) per period (standardised per week), sensitivity analysis*.

Period	Number of packages	Healthcare expenditures	DDDs
During vs before COVID	- 43.0%	- 51.4%	- 40.9%
After vs during COVID	+ 72.1%	+ 81.4%	+ 83.6%

* Sensitivity analysis with only the antibiotics primarily indicated for the treatment of respiratory infections, i.e., amoxicillin, amoxicillin / clavulanate, azithromycin, clarithromycin, sulfamethoxazole and trimethoprim, and phenoxymethylpenicillin.

(a)

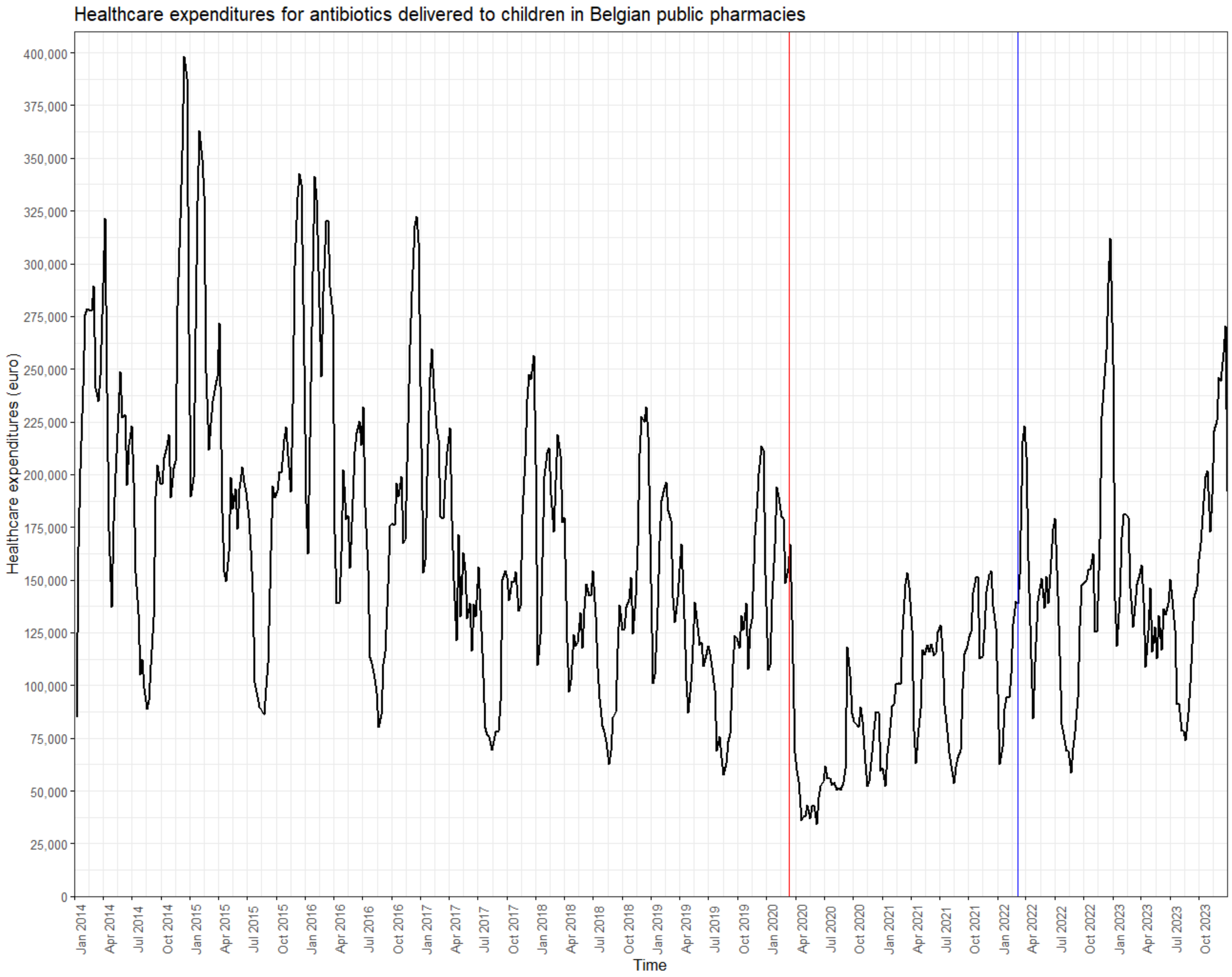
Healthcare expenditures for antibiotics delivered to children in Belgian public pharmacies
Healthcare expenditures (euro)
400,000
375,000
350,000
325,000
300,000
275,000
250,000
225,000
200,000
175,000
150,000
125,000
100,000
75,000
50,000
25,000
0
Jan 2014
Apr 2014
Jul 2014
Oct 2014
Jan 2015
Apr 2015
Jul 2015
Oct 2015
Jan 2016
Apr 2016
Jul 2016
Oct 2016
Jan 2017
Apr 2017
Jul 2017
Oct 2017
Jan 2018
Apr 2018
Jul 2018
Oct 2018
Jan 2019
Apr 2019
Jul 2019
Oct 2019
Jan 2020
Apr 2020
Jul 2020
Oct 2020
Jan 2021
Apr 2021
Jul 2021
Oct 2021
Jan 2022
Apr 2022
Jul 2022
Oct 2022
Jan 2023
Apr 2023
Jul 2023
Oct 2023
Time

(b)

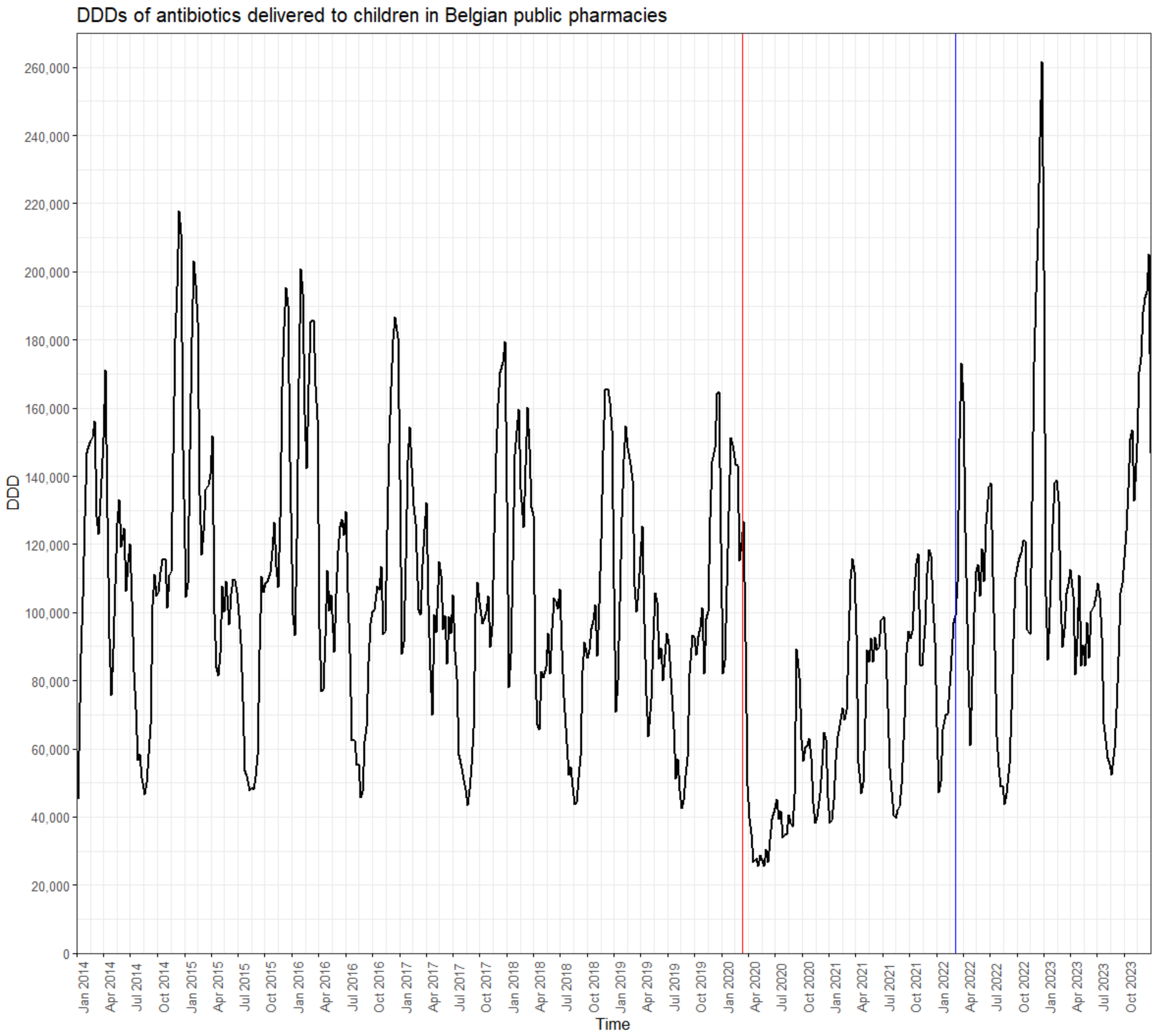

Figure S1. Line chart of antibiotics delivered to children in Belgian public pharmacies, 2014 to 2023, expressed as healthcare expenditures (a) and Defined Daily Doses (DDDs) (b).

The red line represents the start of the COVID-19 pandemic (i.e., the week of 16 March 2020). The blue line represents the start of the post-COVID period (i.e., the week of 14 March 2022).

(a)

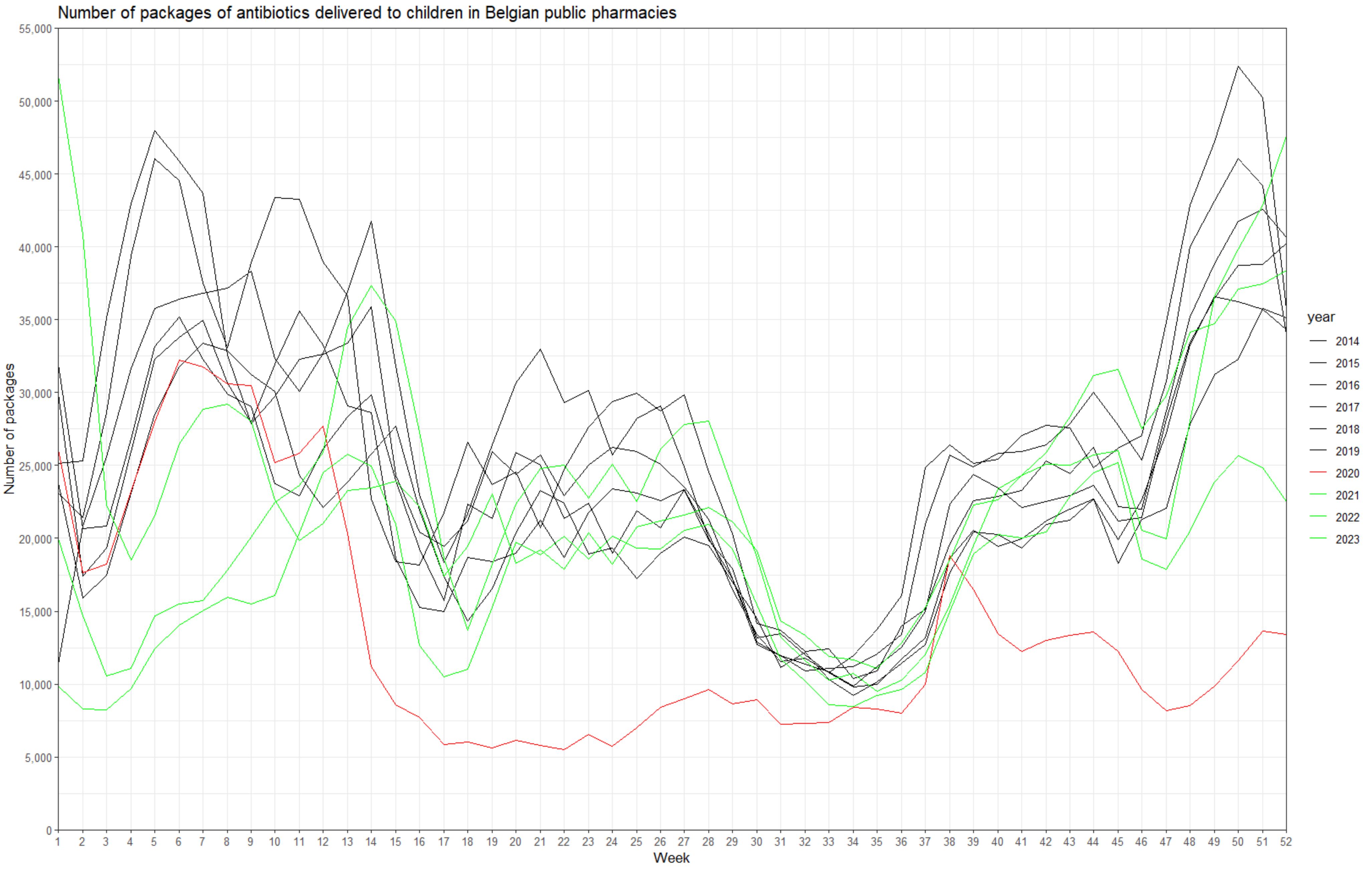

Number of packages of antibiotics delivered to children in Belgian public pharmacies
Number of packages
Week
55,000
50,000
45,000
40,000
35,000
30,000
25,000
20,000
15,000
10,000
5,000
0
1 2 3 4 5 6 7 8 9 10 11 12 13 14 15 16 17 18 19 20 21 22 23 24 25 26 27 28 29 30 31 32 33 34 35 36 37 38 39 40 41 42 43 44 45 46 47 48 49 50 51 52
year
2014
2015
2016
2017
2018
2019
2020
2021
2022
2023

(b)

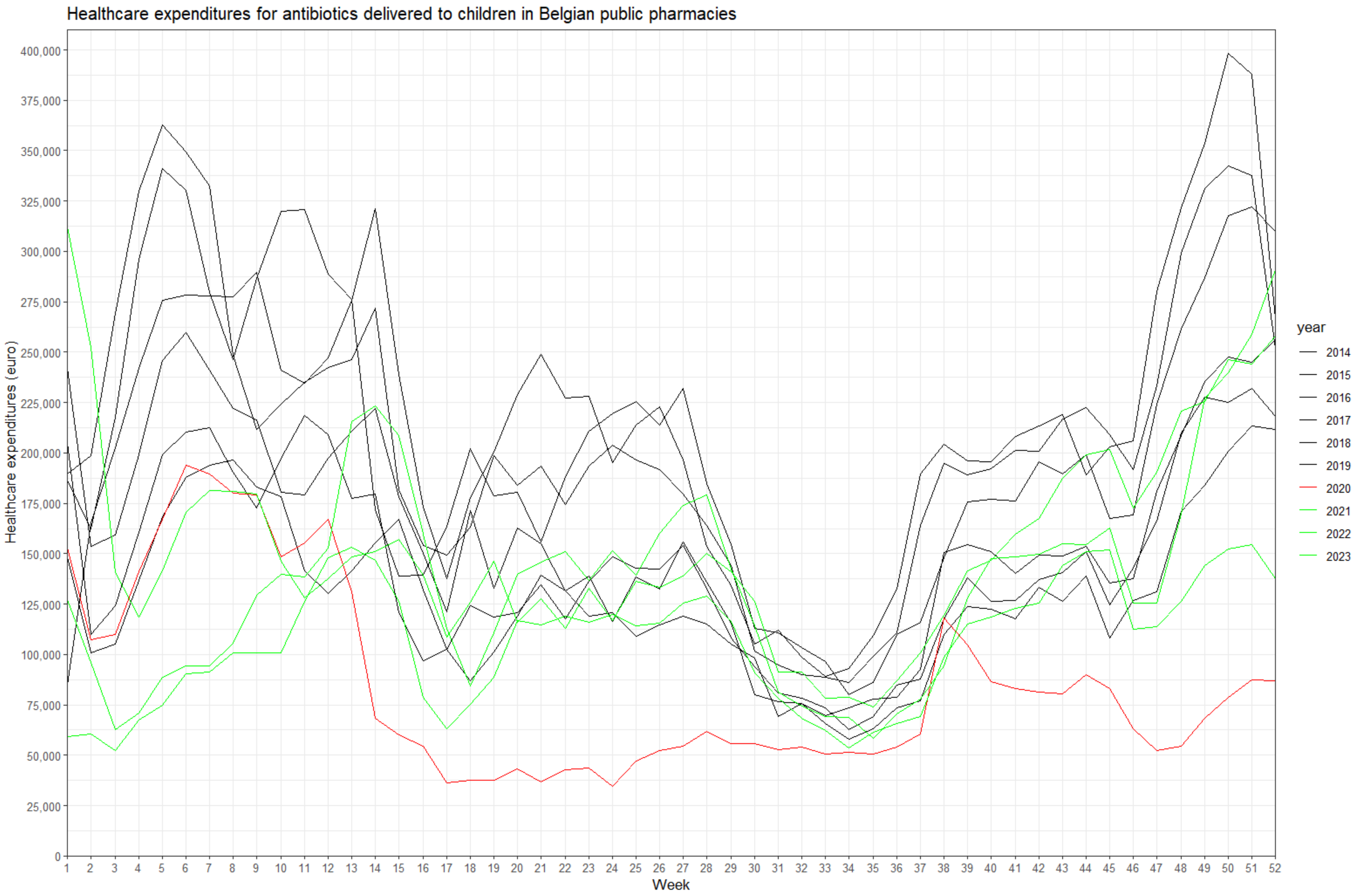
Healthcare expenditures for antibiotics delivered to children in Belgian public pharmacies
Healthcare expenditures (euro)
400,000
375,000
350,000
325,000
300,000
275,000
250,000
225,000
200,000
175,000
150,000
125,000
100,000
75,000
50,000
25,000
0
1 2 3 4 5 6 7 8 9 10 11 12 13 14 15 16 17 18 19 20 21 22 23 24 25 26 27 28 29 30 31 32 33 34 35 36 37 38 39 40 41 42 43 44 45 46 47 48 49 50 51 52
Week
year
2014
2015
2016
2017
2018
2019
2020
2021
2022
2023

(c)

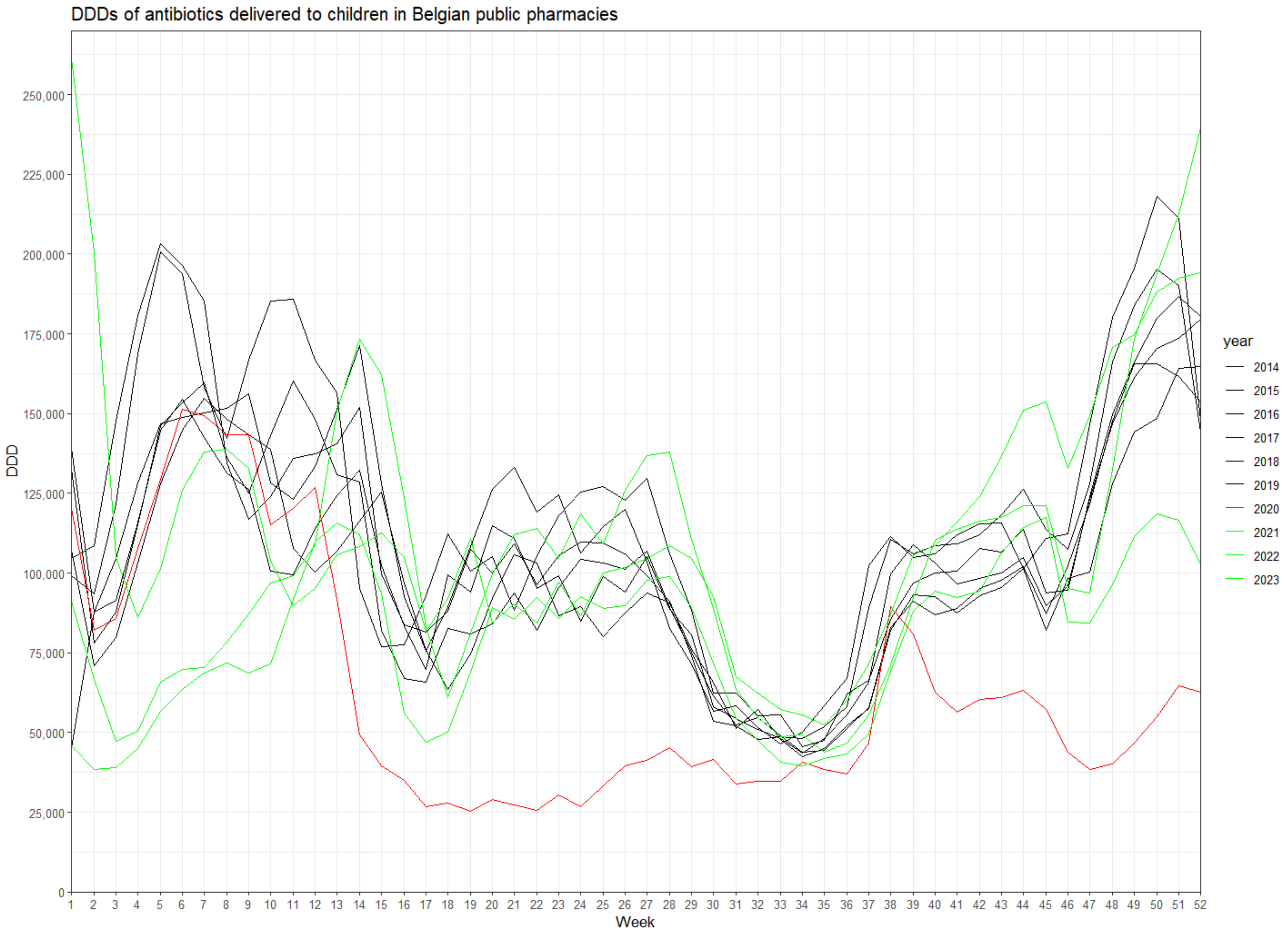

Figure S2. Line chart of antibiotics delivered to children in Belgian public pharmacies by year and week, expressed as number of packages (a), healthcare expenditures (b), and Defined Daily Doses (DDDs) (c).

Black lines represent the years before the COVID pandemic, the red line represents 2020, and the green lines represent the years after 2020.

(a)

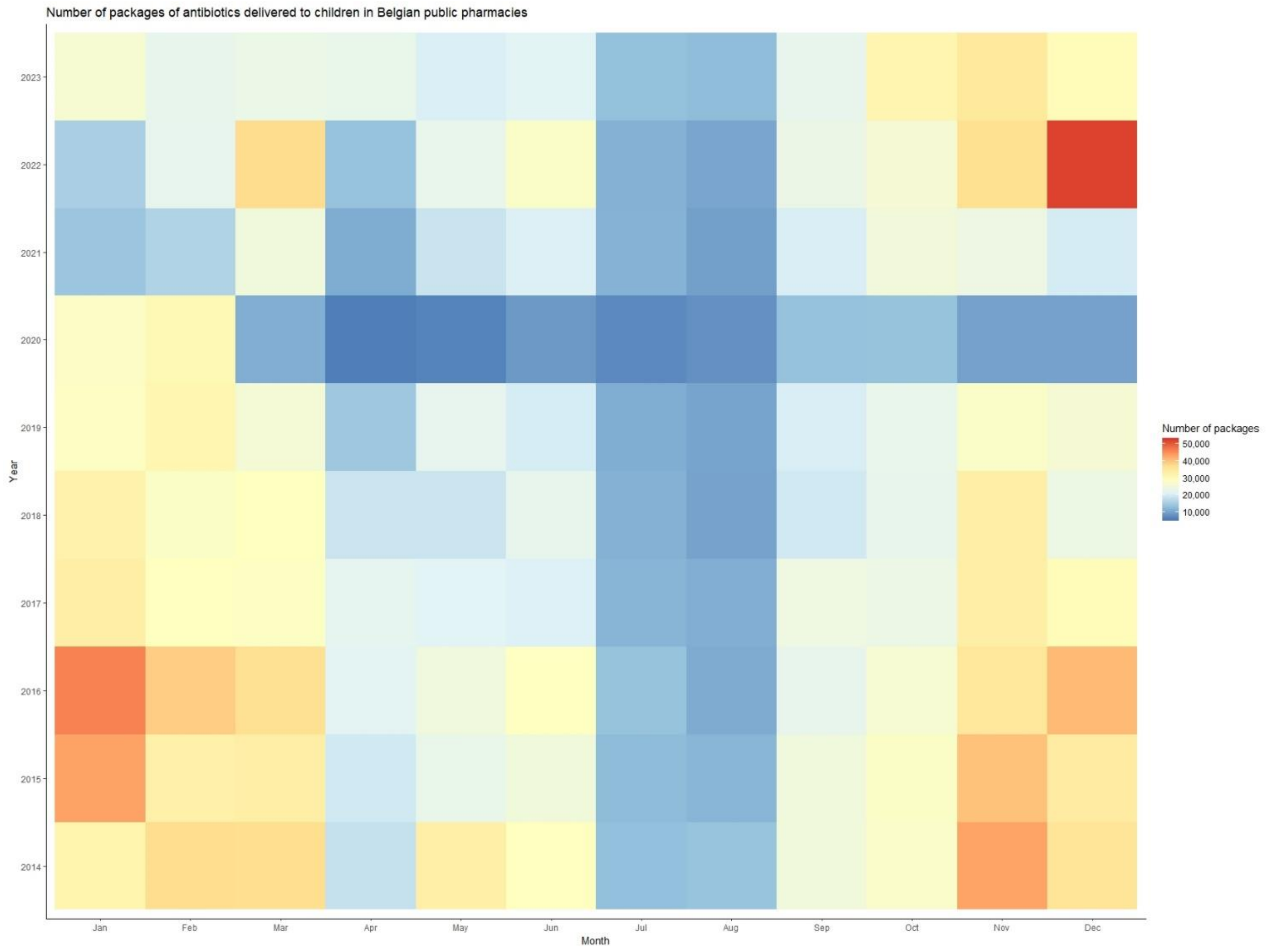
Number of packages of antibiotics delivered to children in Belgian public pharmacies
Year
2023
2022
2021
2020
2019
2018
2017
2016
2015
2014
Month
Jan
Feb
Mar
Apr
May
Jun
Jul
Aug
Sep
Oct
Nov
Dec
Number of packages
50,000
40,000
30,000
20,000
10,000

(b)

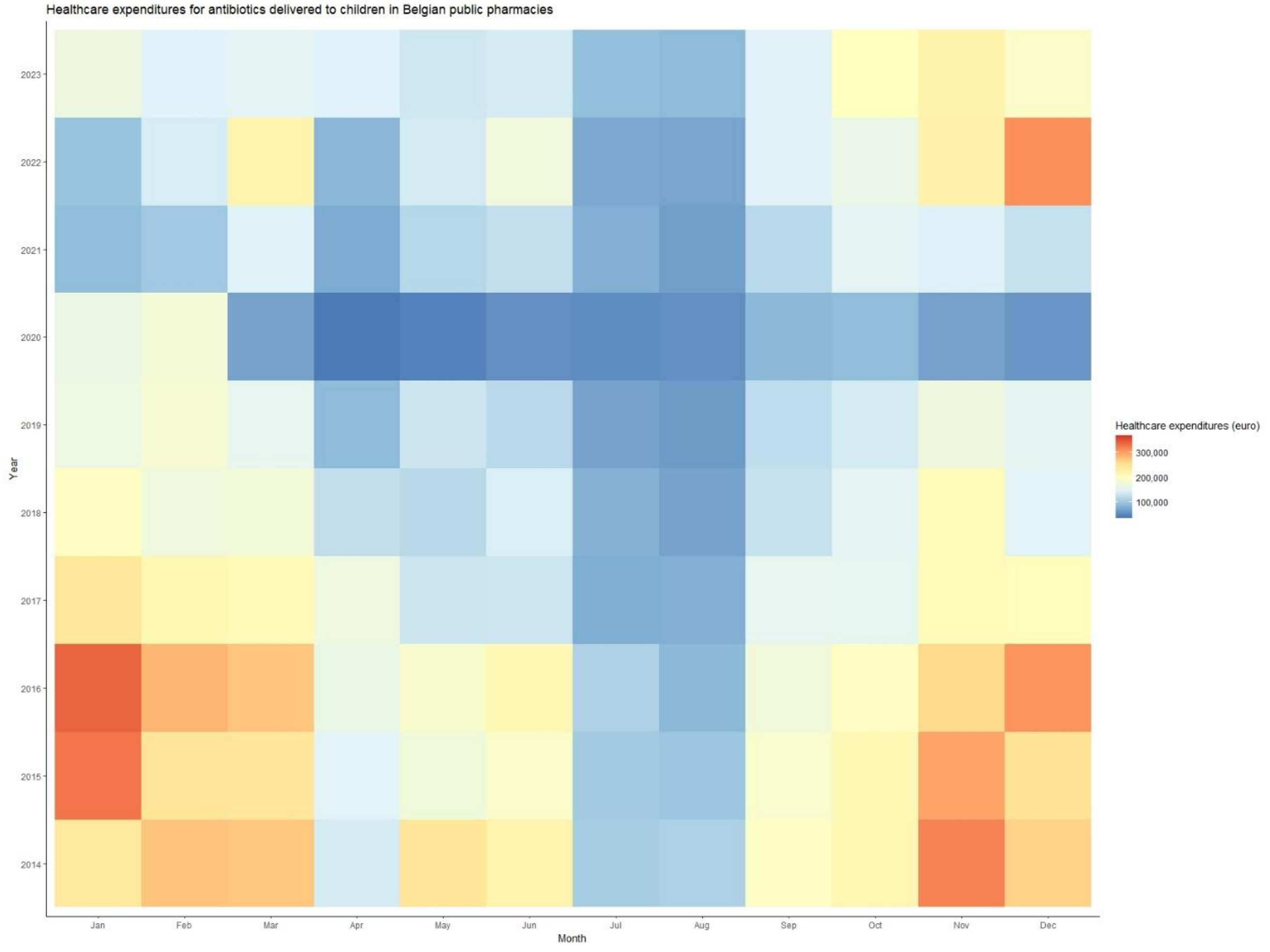
Healthcare expenditures for antibiotics delivered to children in Belgian public pharmacies
Year
2023
2022
2021
2020
2019
2018
2017
2016
2015
2014
Month
Jan
Feb
Mar
Apr
May
Jun
Jul
Aug
Sep
Oct
Nov
Dec
Healthcare expenditures (euro)
300,000
200,000
100,000

(c)

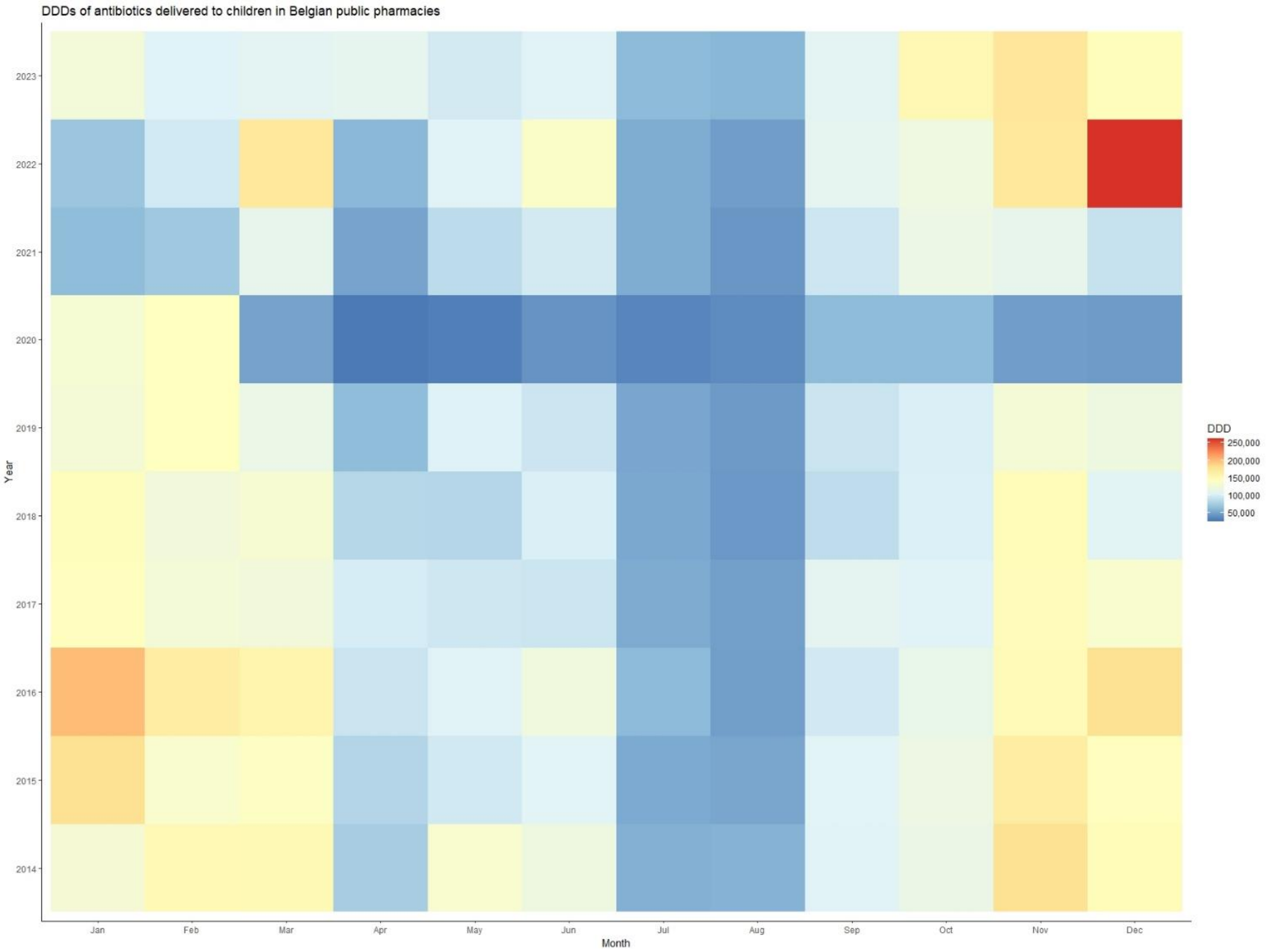

Figure S3. Heatmap of antibiotics delivered to children in Belgian public pharmacies by year and month, expressed as number of packages (a), healthcare expenditures (b), and Defined Daily Doses (DDDs) (c).

Table S6. Results from ARIMA models.

	Number of packages		Healthcare expenditures		Defined Daily Doses (DDDs)	
Augmented Dickey-Fuller test	p<0.01 at lag order 8		p<0.01 at lag order 8		p<0.01 at lag order 8	
Combined test for seasonality	TRUE		TRUE		TRUE	
	Model without external regressors	Model with external regressors	Model without external regressors	Model with external regressors	Model without external regressors	Model with external regressors
model	(3,1,1)(0,1,1)	(3,1,1)(0,1,1)	(1,1,2)(2,1,0)	(2,1,1)(1,1,0)	(3,1,1)(0,1,1)	(3,1,1)(0,1,1)
ar1 [95% CI]	**0.64 [0.37 to 0.91]**	**0.65 [0.34 to 0.96]**	**0.71 [0.61 to 0.81]**	**1.01 [0.91 to 1.11]**	**0.70 [0.41 to 0.99]**	**0.71 [0.33 to 1.07]**
ar2 [95% CI]	**-0.25 [-0.38 to -0.12]**	**-0.29 [-0.44 to -0.14]**	NA	**-0.28 [-0.37 to -0.18]**	**-0.27 [-0.40 to -0.13]**	**-0.33 [-0.50 to -0.15]**
ar3 [95% CI]	**-0.16 [-0.29 to -0.034]**	**-0.15 [-0.29 to -0.0032]**	NA	NA	-0.13 [-0.26 to 0.0036]	-0.11 [-0.28 to 0.054]
ma1 [95% CI]	**-0.46 [-0.73 to -0.20]**	**-0.45 [-0.76 to -0.13]**	**-0.67 [-0.78 to -0.56]**	**-0.95 [-1.01 to -0.89]**	**-0.52 [-0.81 to -0.24]**	**-0.47 [-0.84 to -0.11]**
ma2 [95% CI]	NA	NA	**-0.27 [-0.37 to -0.18]**	NA	NA	NA
sar1 [95% CI]	NA	NA	**-0.40 [-0.50 to -0.31]**	**-0.30 [-0.39 to -0.20]**	NA	NA
sar2 [95% CI]	NA	NA	**-0.33 [-0.43 to -0.22]**	NA	NA	NA
sma1 [95% CI]	**-0.53 [-0.63 to -0.42]**	**-0.52 [-0.62 to -0.42]**	NA	NA	**-0.53 [-0.63 to -0.42]**	**-0.52 [-0.62 to -0.42]**
xreg1 [95% CI] (during-COVID period)	NA	**-6,106 [-10,817 to -1,395]**	NA	**-45,657 [-74,504 to -16,809]**	NA	**-25,216 [-46,958 to -3,473]**
xreg2 [95% CI] (post-COVID period)	NA	1,049 [-6,056 to 8,153]	NA	-3,587 [-48,668 to 41,494]	NA	7,382 [-25,381 to 39,945]
xreg3 [95% CI] (proportion attributed to girls)	NA	-5,119 [-31,971 to 21,733]	NA	-22,817 [-86,741 to 41,106]	NA	-63,999 [-195,047 to 67,049]
xreg4 [95% CI] (proportion attributed to young children)	NA	**-20,418 [-29,732 to -11,104]**	NA	**-137,294 [-182,694 to -91,894]**	NA	**-121,627 [-159,941 to -83,313]**
sigma^2	8,760,718	8,182,106	448,703,596	441,236,879	192,213,032	172,831,607
log likelihood	-4,420	-4,402	-5,343	-5,332	-5,145	-5,117

AIC	8,853	8,824	10,699	10,682	10,301	10,255
AICc	8,853	8,824	10,699	10,683	10,301	10,255
BIC	8,878	8,865	10,724	10,720	10,326	10,296
ME	-55	-70	-328	-715	-221	-292
RMSE	2,791	2,685	19,971	19,740	13,071	12,341
MAE	1,896	1,838	13,654	13,443	8,836	8,405
MPE	-1.09	-1.09	-1.02	-1.06	-1.20	-1.20
MAPE	9.68	9.60	10.51	10.46	9.93	9.68
MASE	0.37	0.35	0.39	0.38	0.38	0.36
ACF1	-0.0077	-0.0081	0.023	0.0097	-0.0055	-0.0071
Ljung-Box test	p<0.001	p<0.001	p=0.0033	p<0.001	p<0.001	p<0.001

Significant values are indicated in **bold**.
Abbreviations: NA, not applicable; ar, autoregressive terms; ma, moving average; sar, seasonal autoregressive terms; sma, seasonal moving average; xreg, external regressor; AIC, Akaike information criterion; BIC, Bayesian information criterion; ME, mean error; RMSE, root mean square error; MAE, mean absolute error; MPE, mean percentage error; MAPE, mean absolute percentage error; MASE, mean absolute scaled error; ACF1, first lag autocorrelation of residuals.

(a)

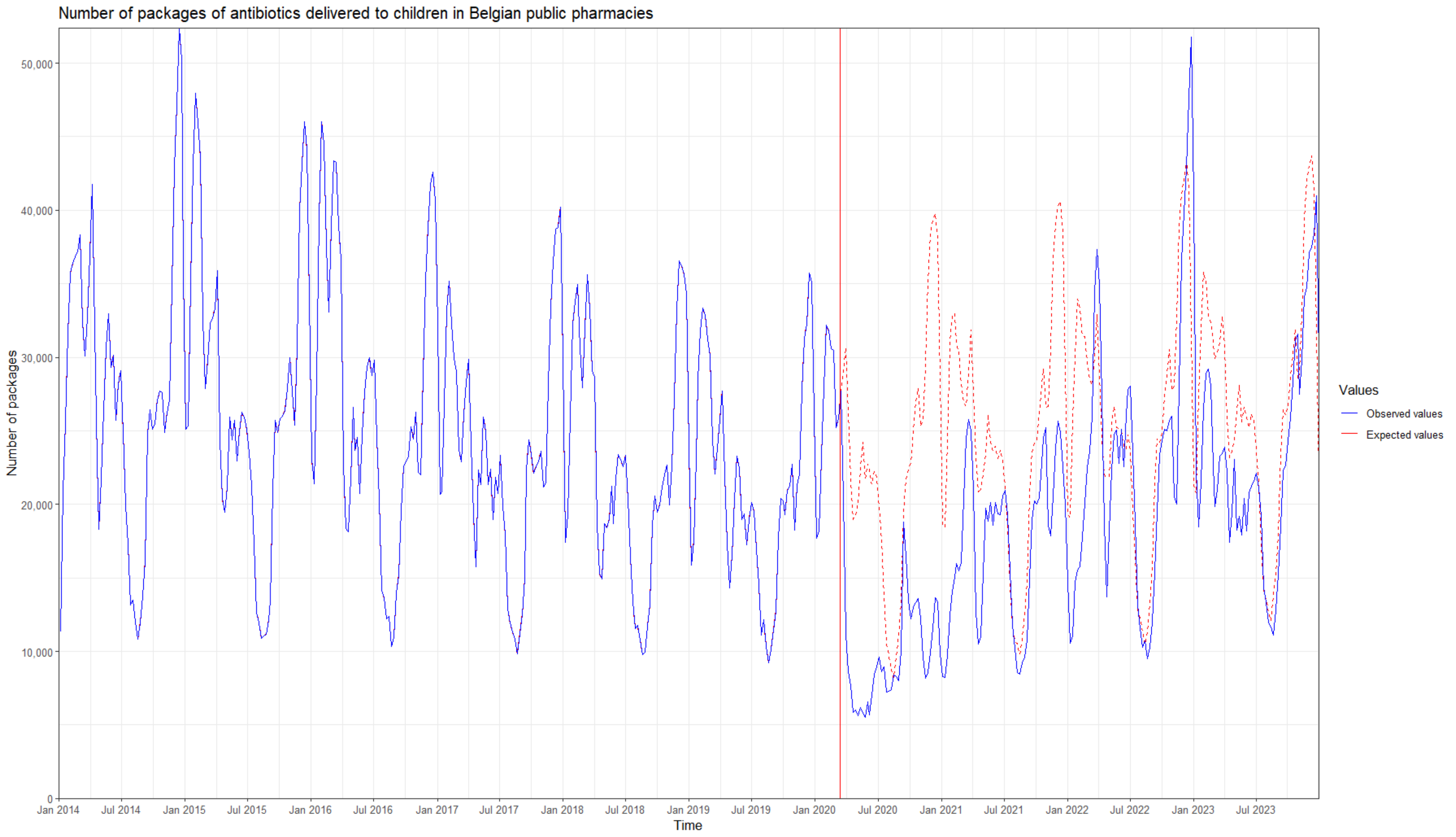

Number of packages of antibiotics delivered to children in Belgian public pharmacies
Number of packages
50,000
40,000
30,000
20,000
10,000
0
Jan 2014
Jul 2014
Jan 2015
Jul 2015
Jan 2016
Jul 2016
Jan 2017
Jul 2017
Jan 2018
Jul 2018
Jan 2019
Jul 2019
Jan 2020
Jul 2020
Jan 2021
Jul 2021
Jan 2022
Jul 2022
Jan 2023
Jul 2023
Time
Values
Observed values
Expected values

(b)

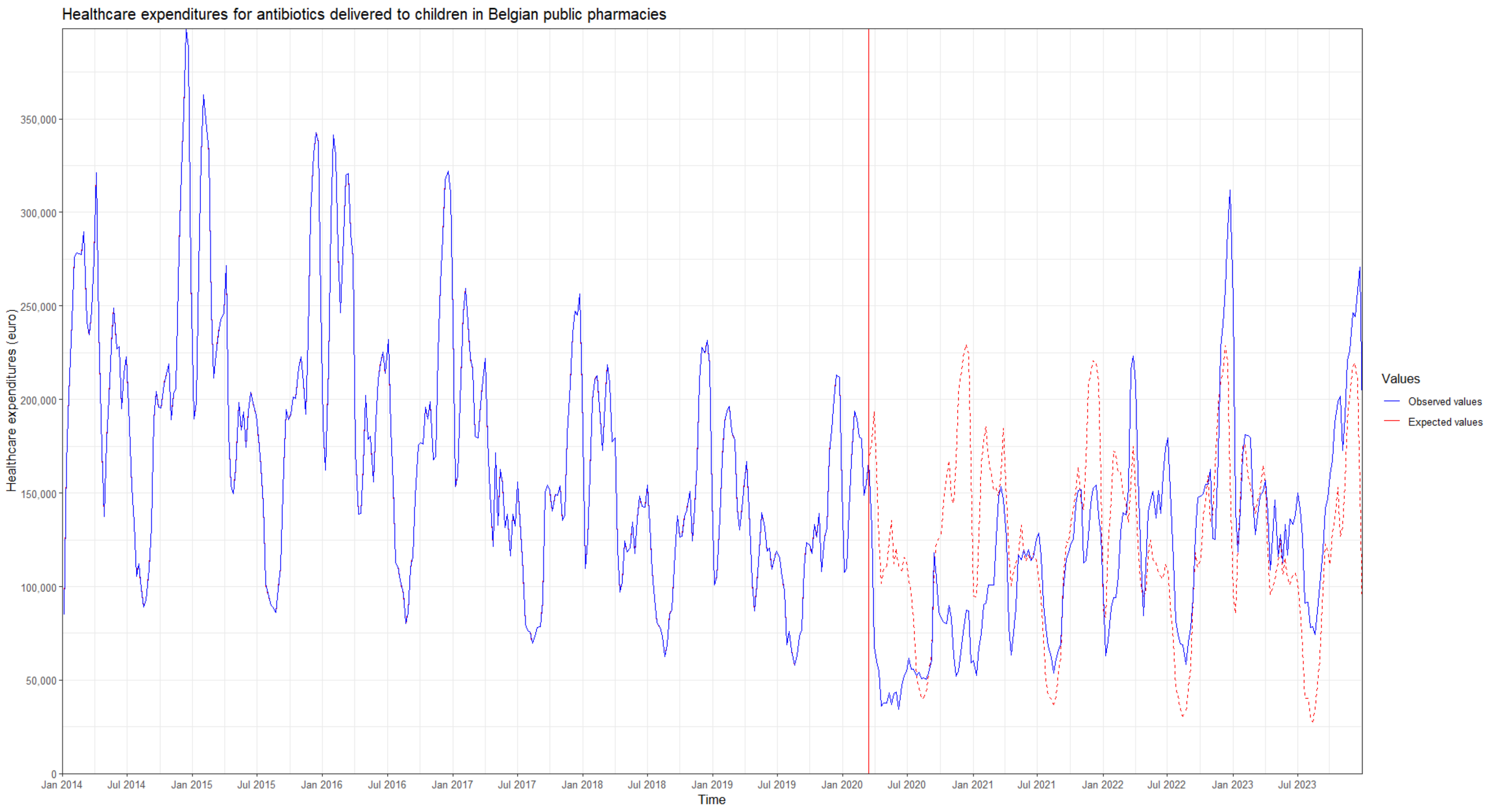
Healthcare expenditures for antibiotics delivered to children in Belgian public pharmacies
Healthcare expenditures (euro)
350,000
300,000
250,000
200,000
150,000
100,000
50,000
0
Jan 2014
Jul 2014
Jan 2015
Jul 2015
Jan 2016
Jul 2016
Jan 2017
Jul 2017
Jan 2018
Jul 2018
Jan 2019
Jul 2019
Jan 2020
Jul 2020
Jan 2021
Jul 2021
Jan 2022
Jul 2022
Jan 2023
Jul 2023
Time
Values
Observed values
Expected values

(c)

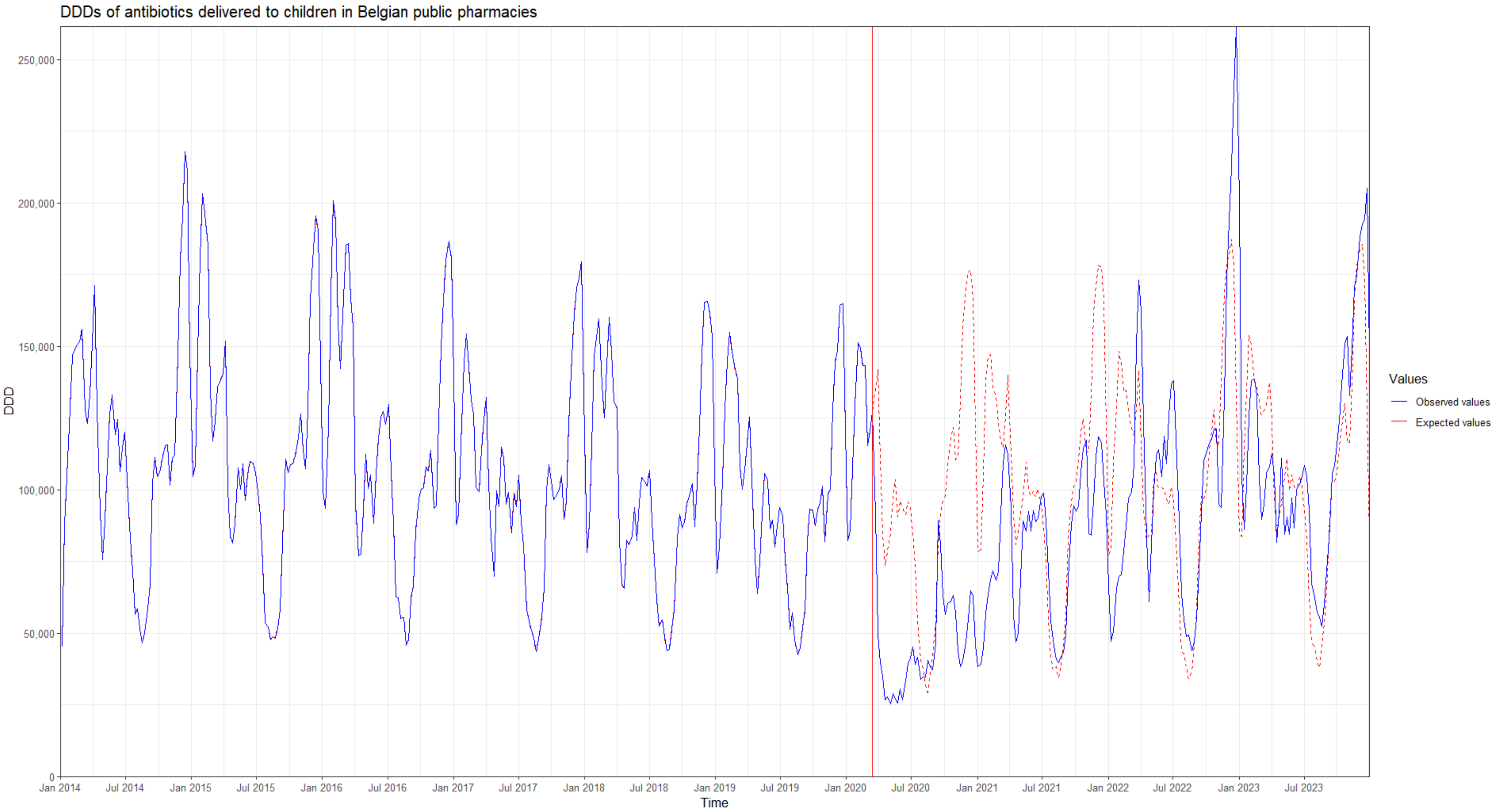

Figure S4. Antibiotics delivered to children in Belgian public pharmacies: observed and expected outcomes of the number of packages (a), healthcare expenditures (b), and Defined Daily Doses (DDDs) (c).

The solid blue line represents the observed outcomes. The dashed red line represents the expected outcomes (i.e., assumption of no COVID-19 pandemic). The vertical solid red line represents the start of the COVID-19 pandemic (i.e., the week of 16 March 2020).

Table S7. Accuracy measures from ARIMA models based on the outcomes before the COVID-19 pandemic.

	Number of packages	Healthcare expenditures	Defined Daily Doses (DDDs)
ME	-63.66	-986	-678
RMSE	2,867	19,186	11,003
MAE	1,838	12,393	7,139
MPE	-0.89	-0.74	-1.11
MAPE	7.97	7.83	6.99
MASE	0.54	0.43	0.50
ACF1	-0.026	-0.0015	-0.0071

Abbreviations: ME, mean error; RMSE, root mean square error; MAE, mean absolute error; MPE, mean percentage error; MAPE, mean absolute percentage error; MASE, mean absolute scaled error; ACF1, first lag autocorrelation of residuals.

Table S8. Results from ARIMA models, sensitivity analysis*.

	Number of packages		Healthcare expenditures		Defined Daily Doses (DDDs)	
Augmented Dickey-Fuller test	p<0.01 at lag order 8		p<0.01 at lag order 8		p<0.01 at lag order 8	
Combined test for seasonality	TRUE		TRUE		TRUE	
	Model without external regressors	Model with external regressors	Model without external regressors	Model with external regressors	Model without external regressors	Model with external regressors
model	(5,1,2)(0,1,1)	(3,1,1)(0,1,1)	(5,1,2)(0,1,1)	(2,1,2)(0,1,1)	(3,1,1)(0,1,1)	(3,1,1)(0,1,1)
ar1 [95% CI]	**0.32 [0.14 to 0.51]**	**0.70 [0.35 to 1.05]**	**0.31 [0.13 to 0.48]**	**1.02 [0.87 to 1.17]**	**0.73 [0.43 to 1.04]**	**0.74 [0.35 to 1.13]**
ar2 [95% CI]	**0.60 [0.39 to 0.81]**	**-0.33 [-0.49 to -0.16]**	**0.59 [0.40 to 0.78]**	**-0.65 [-0.83 to -0.47]**	**-0.30 [-0.45 to -0.15]**	**-0.34 [-0.54 to -0.15]**
ar3 [95% CI]	**-0.39 [-0.52 to -0.26]**	-0.12 [-0.28 to 0.038]	**-0.38 [-0.51 to -0.26]**	NA	-0.12 [-0.26 to 0.022]	-0.10 [-0.28 to 0.074]
ar4 [95% CI]	0.031 [-0.071 to 0.13]	NA	-0.0016 [-0.10 to 0.098]	NA	NA	NA
ar5 [95% CI]	**0.20 [0.10 to 0.29]**	NA	**0.23 [0.13 to 0.32]**	NA	NA	NA
ma1 [95% CI]	-0.12 [-0.28 to 0.048]	**-0.45 [-0.80 to -0.10]**	-0.13 [-0.29 to 0.026]	**-0.82 [-1.00 to -0.63]**	**-0.51 [-0.82 to -0.21]**	**-0.47 [-0.86 to -0.082]**
ma2 [95% CI]	**-0.84 [-1.00 to -0.68]**	NA	**-0.81 [-0.96 to -0.67]**	**0.29 [0.055 to 0.52]**	NA	NA
sma1 [95% CI]	**-0.52 [-0.62 to -0.41]**	**-0.53 [-0.63 to -0.43]**	-0.49 [-0.59 to -0.38]	-0.50 [-0.61 to -0.40]	**-0.53 [-0.64 to -0.43]**	**-0.53 [-0.63 to -0.43]**
xreg1 [95% CI] (during-COVID period)	NA	**-5,434 [-9,807 to -1,060]**	NA	**-35,994 [-64,430 to -7,557]**	NA	**-23,049 [-43,003 to -3,094]**
xreg2 [95% CI] (post-COVID period)	NA	1,176 [-5,422 to 7,775]	NA	-1,503 [-44,906 to 41,900]	NA	4,930 [-25,088 to 34,949]
xreg3 [95% CI] (proportion attributed to girls)	NA	-2,666 [-27,176 to 21,843]	NA	-7,049 [-60,614 to 46,515]	NA	-71,917 [-190,467 to 46,632]

xreg4 [95% CI] (proportion attributed to young children)	NA	**-16,546 [-25,071 to -8,022]**	NA	**-53,699 [-90,976 to -16,423]**	NA	**-104,033 [-138,804 to -69,261]**
sigma^2	7,336,795	7,101,686	312,855,248	308,737,580	162,040,112	147,294,618
log likelihood	-4,377	-4,369	-5,256	-5,253	-5,105	-5,080
AIC	8,773	8,758	10,531	10,526	10,221	10,180
AICc	8,773	8,758	10,531	10,526	10,222	10,181
BIC	8,810	8,800	10,568	10,567	10,246	10,222
ME	-52	-63	-384	-396	-197	-256
RMSE	2,545	2,502	16,622	16,494	12,001	11,393
MAE	1,715	1,712	11,094	11,209	8,113	7,758
MPE	-1.46	-1.18	-1.15	-1.01	-1.28	-1.28
MAPE	9.73	9.91	10.11	10.32	10.30	10.07
MASE	0.35	0.35	0.34	0.34	0.37	0.35
ACF1	-0.0060	-0.0081	0.0089	-0.019	-0.0050	-0.0063
Ljung-Box test	p=0.0032	p<0.001	p=0.0038	p<0.001	p<0.001	p<0.001

* Sensitivity analysis with only the antibiotics primarily indicated for the treatment of respiratory infections, i.e., amoxicillin, amoxicillin / clavulanate, azithromycin, clarithromycin, sulfamethoxazole and trimethoprim, and phenoxymethylpenicillin.

Significant values are indicated in **bold**.

Abbreviations: NA, not applicable; ar, autoregressive terms; ma, moving average; sma, seasonal moving average; xreg, external regressor; AIC, Akaike information criterion; BIC, Bayesian information criterion; ME, mean error; RMSE, root mean square error; MAE, mean absolute error; MPE, mean percentage error; MAPE, mean absolute percentage error; MASE, mean absolute scaled error; ACF1, first lag autocorrelation of residuals

(a)

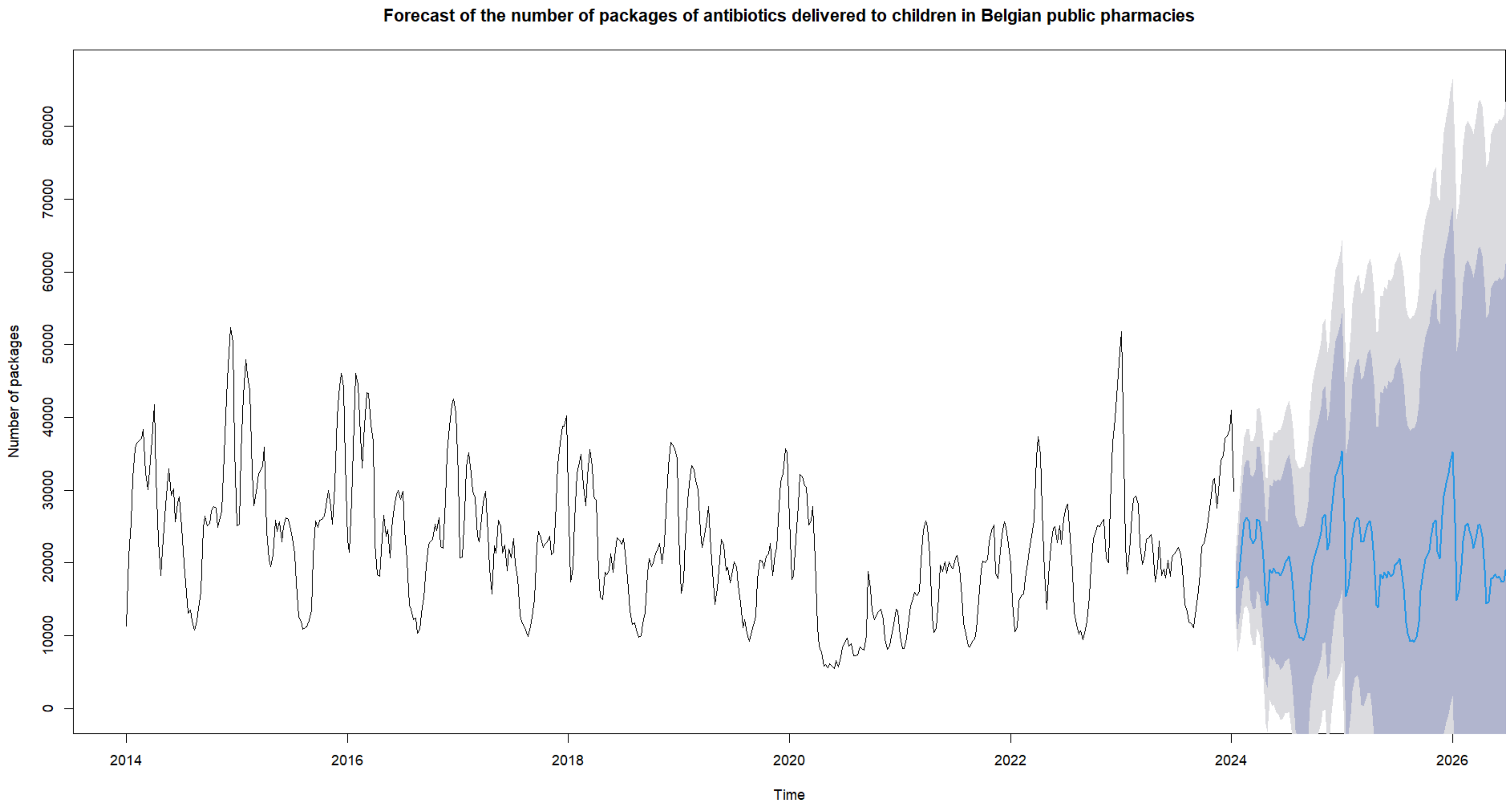

Forecast of the number of packages of antibiotics delivered to children in Belgian public pharmacies
Number of packages
0
10000
20000
30000
40000
50000
60000
70000
80000
2014
2016
2018
2020
2022
2024
2026
Time

(b)

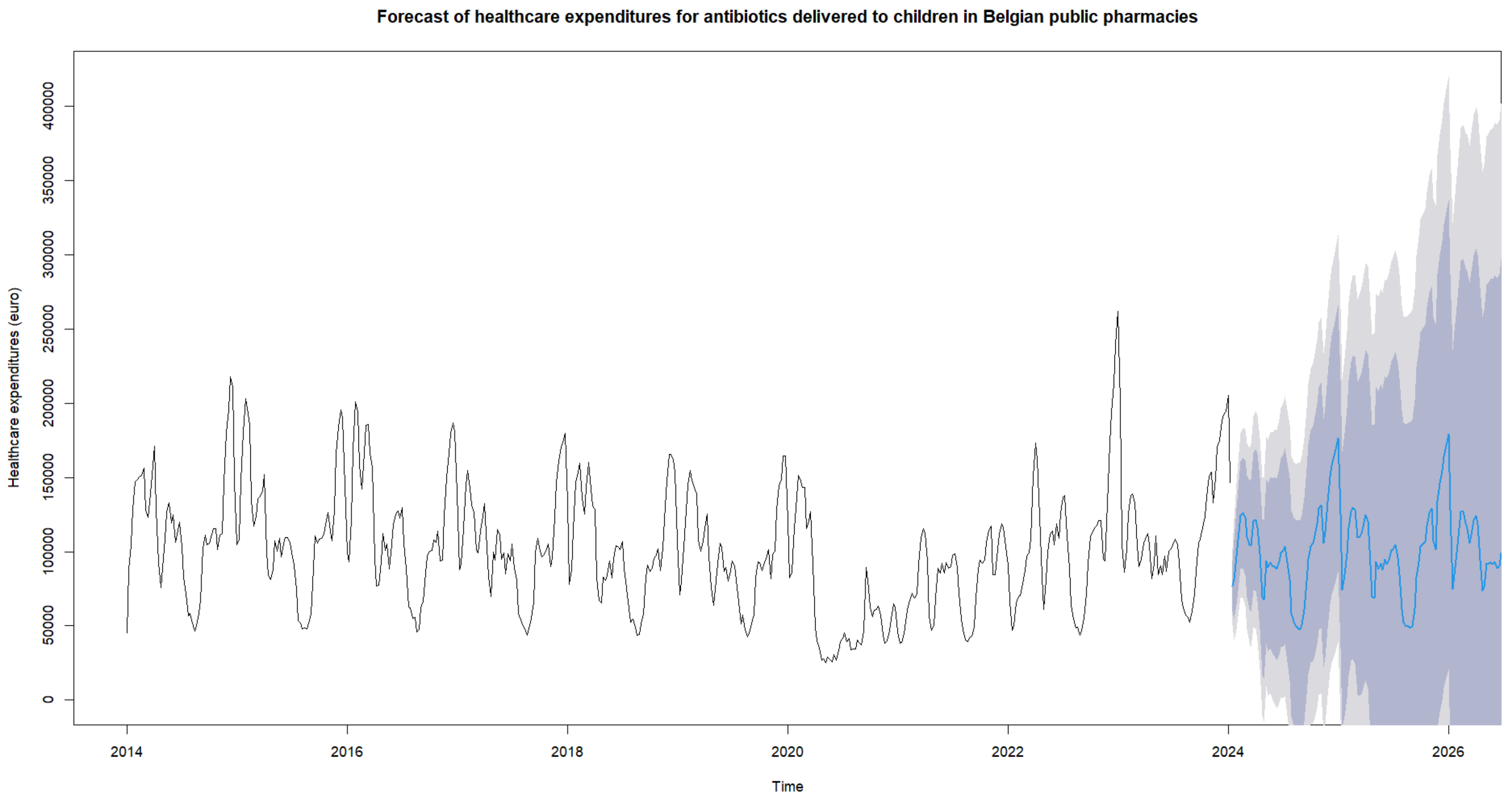
Forecast of healthcare expenditures for antibiotics delivered to children in Belgian public pharmacies
Healthcare expenditures (euro)
0
50000
100000
150000
200000
250000
300000
350000
400000
2014
2016
2018
2020
2022
2024
2026
Time

(c)

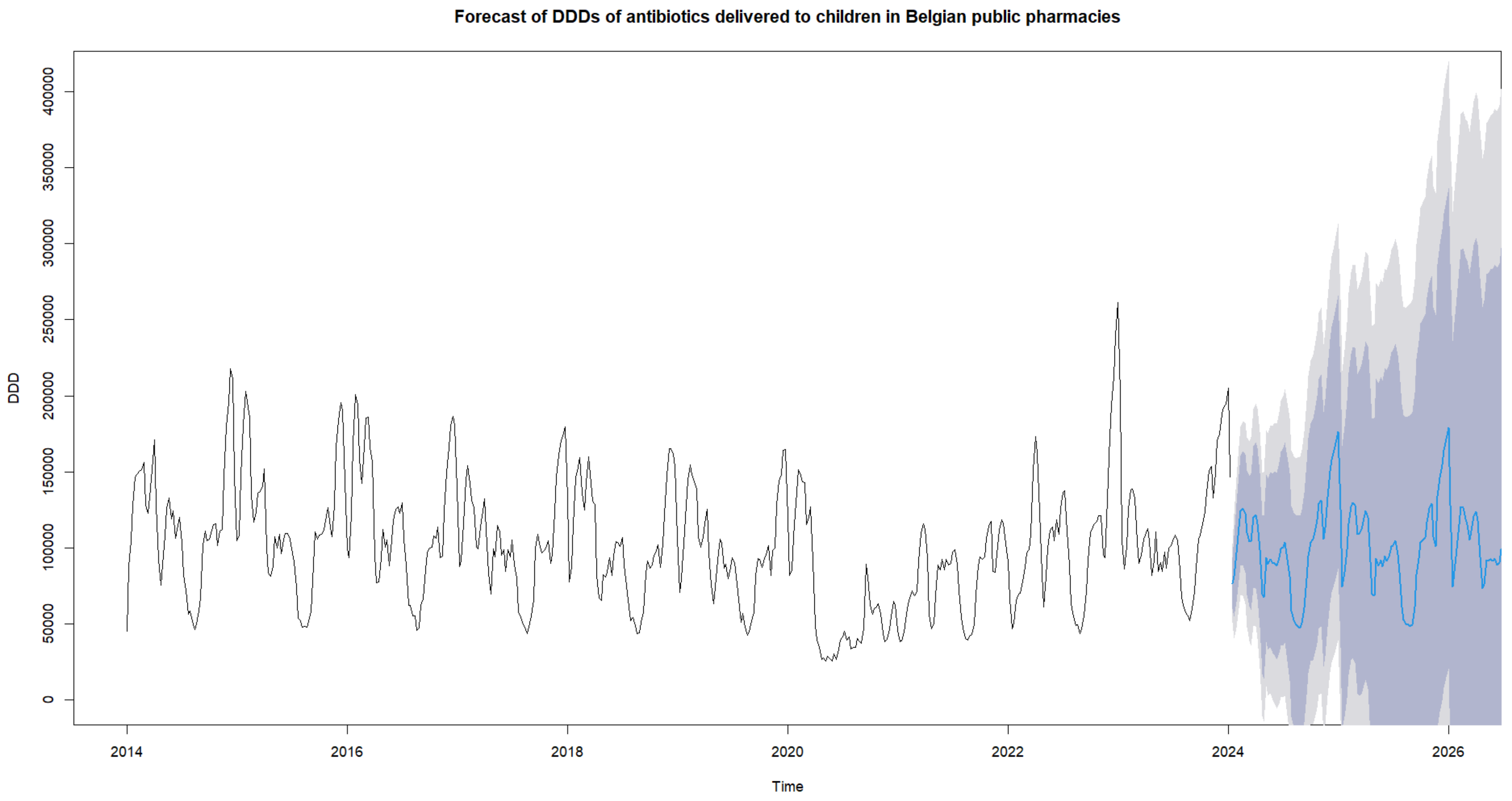

Figure S5. Forecast of antibiotics delivered to children in Belgian public pharmacies, expressed as number of packages (a), healthcare expenditures (b), and Defined Daily Doses (DDDs) (c).
The light blue line represents the point forecasts. The blue and grey shades represent the 80% and 95% prediction intervals, respectively.

PATIENT CHARACTERISTICS

Table S9. Relative change of number of packages, healthcare expenditures and Defined Daily Doses (DDDs) by age group and sex, per period (standardised per week).

	Number of packages	Healthcare expenditures	DDDs
During vs before COVID			
Boys	- 42.3%	- 48.8%	- 39.8%
Girls	- 43.1%	- 49.4%	- 39.3%
0- to 1-year-olds	- 34.0%	- 42.5%	- 27.3%
2- to 6-year-olds	- 42.7%	- 50.0%	- 38.2%
7- to 12-year-olds	- 47.5%	- 50.9%	- 45.2%
0- to 1-year-old boys	- 33.8%	- 42.2%	- 27.5%
2- to 6-year-old boys	- 42.6%	- 50.0%	- 38.5%
7- to 12-year-old boys	- 47.1%	- 50.6%	- 46.3%
0- to 1-year-old girls	- 34.3%	- 42.9%	- 27.1%
2- to 6-year-old girls	- 42.8%	- 50.0%	- 37.8%
7- to 12-year-old girls	- 47.9%	- 51.2%	- 44.2%
After vs during COVID			
Boys	+ 67.1%	+ 69.2%	+ 76.4%
Girls	+ 66.7%	+ 68.4%	+ 71.0%
0- to 1-year-olds	+ 46.6%	+ 54.1%	+ 47.1%
2- to 6-year-olds	+ 62.7%	+ 67.5%	+ 68.0%
7- to 12-year-olds	+ 89.2%	+ 78.7%	+ 93.2%
0- to 1-year-old boys	+ 4.37%	+ 8.75%	+ 4.44%
2- to 6-year-old boys	+ 28.8%	+ 30.9%	+ 33.1%
7- to 12-year-old boys	+ 43.5%	+ 35.9%	+ 49.3%
0- to 1-year-old girls	+ 3.61%	+ 7.28%	+ 3.14%
2- to 6-year-old girls	+ 28.4%	+ 31.6%	+ 30.5%
7- to 12-year-old girls	+ 38.8%	+ 29.5%	+ 37.4%

Table S10. Relative change of number of packages, healthcare expenditures and Defined Daily Doses (DDDs) by reimbursement type, per period (standardised per week).

	Number of packages	Healthcare expenditures	DDDs
During vs before COVID			
Standard reimbursement	- 43.5%	- 50.5%	- 40.8%
Increased reimbursement	- 39.8%	- 44.9%	- 35.0%
After vs during COVID			
Standard reimbursement	+ 68.5%	+ 75.1%	+ 75.7%
Increased reimbursement	+ 61.5%	+ 52.0%	+ 67.5%

(a)

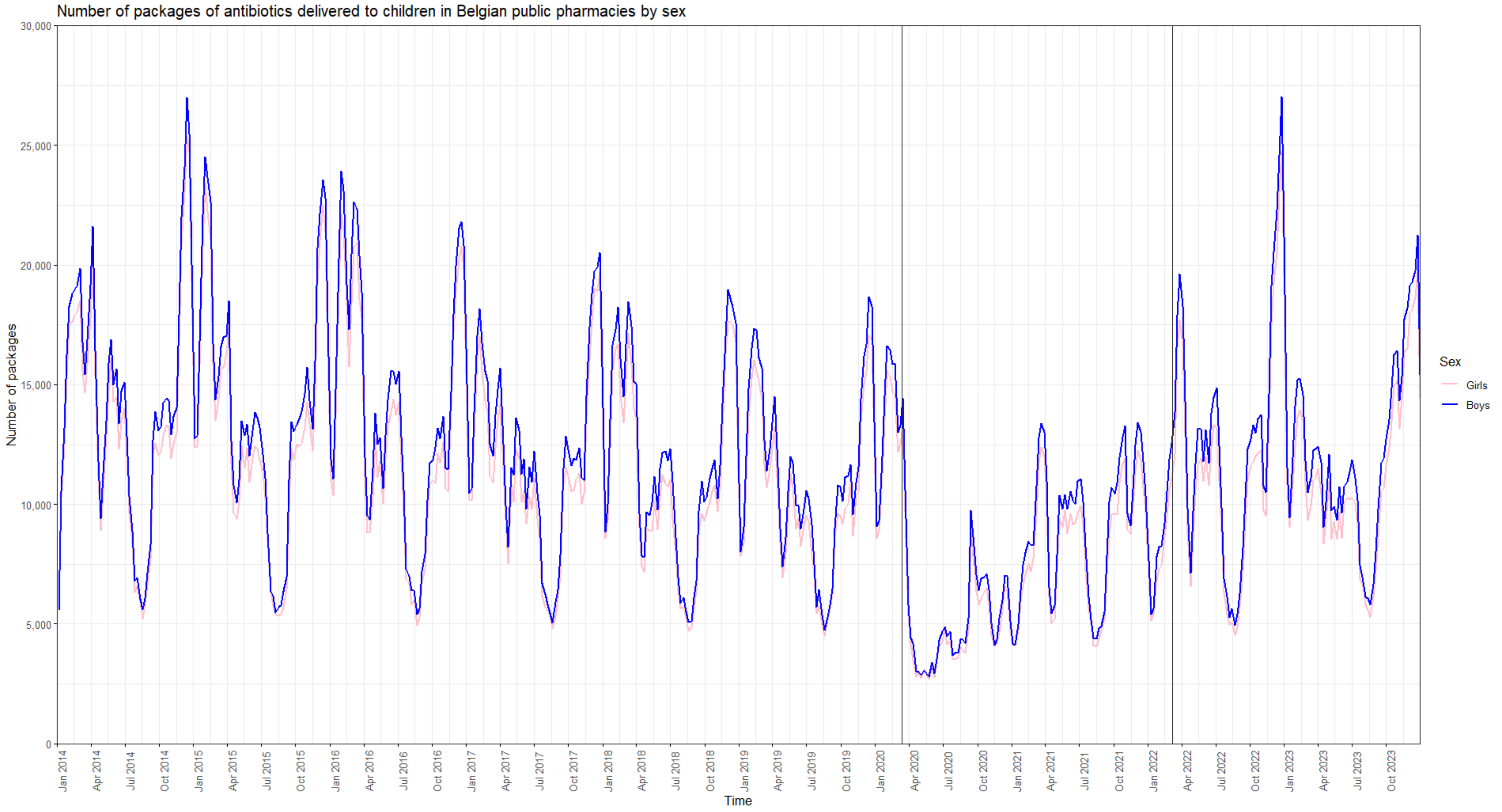

Number of packages of antibiotics delivered to children in Belgian public pharmacies by sex
Number of packages
30,000
25,000
20,000
15,000
10,000
5,000
0
Jan 2014
Apr 2014
Jul 2014
Oct 2014
Jan 2015
Apr 2015
Jul 2015
Oct 2015
Jan 2016
Apr 2016
Jul 2016
Oct 2016
Jan 2017
Apr 2017
Jul 2017
Oct 2017
Jan 2018
Apr 2018
Jul 2018
Oct 2018
Jan 2019
Apr 2019
Jul 2019
Oct 2019
Jan 2020
Apr 2020
Jul 2020
Oct 2020
Jan 2021
Apr 2021
Jul 2021
Oct 2021
Jan 2022
Apr 2022
Jul 2022
Oct 2022
Jan 2023
Apr 2023
Jul 2023
Oct 2023
Time
Sex
Girls
Boys

(b)

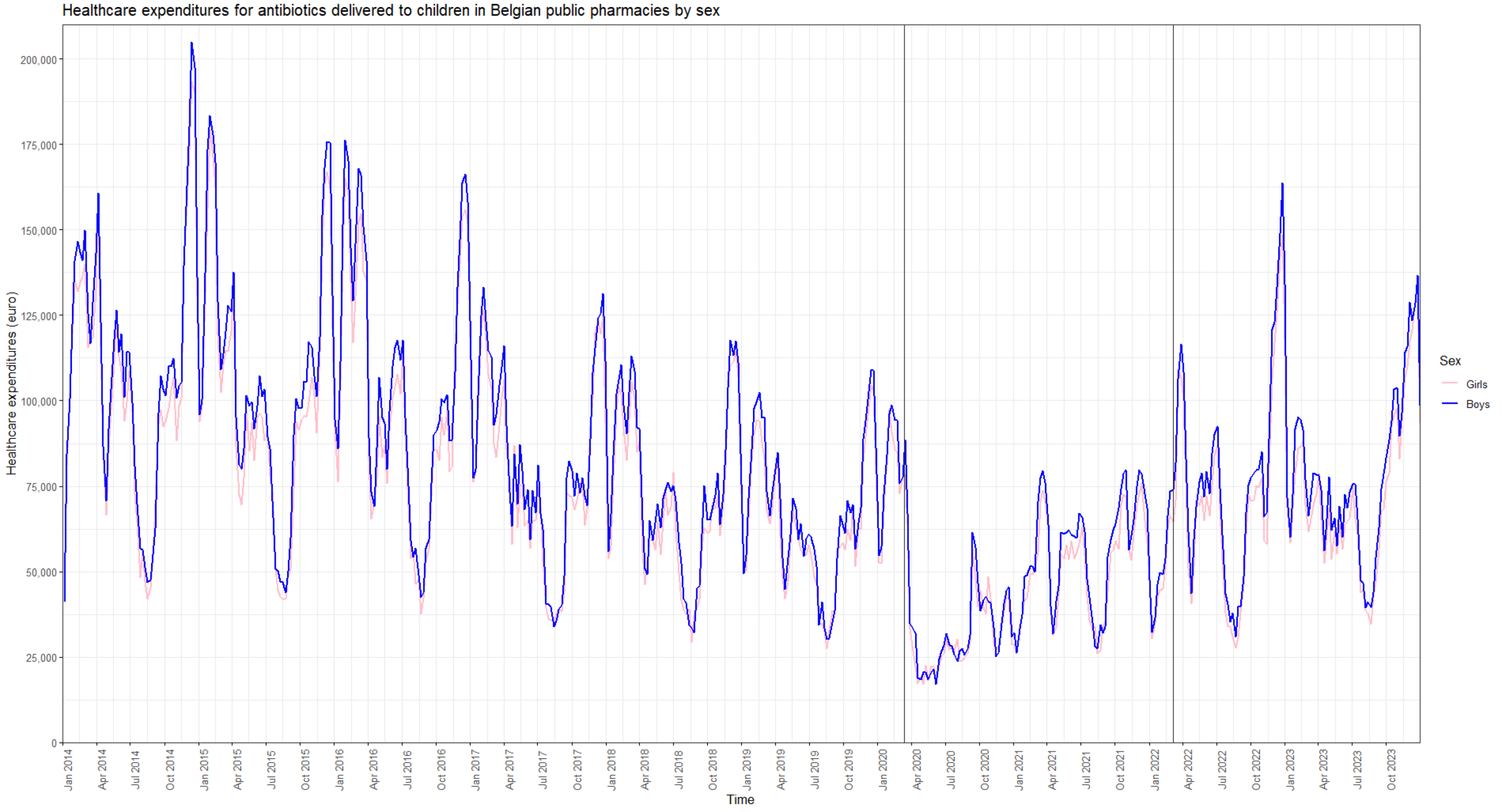
Healthcare expenditures for antibiotics delivered to children in Belgian public pharmacies by sex
Healthcare expenditures (euro)
200,000
175,000
150,000
125,000
100,000
75,000
50,000
25,000
0
Jan 2014
Apr 2014
Jul 2014
Oct 2014
Jan 2015
Apr 2015
Jul 2015
Oct 2015
Jan 2016
Apr 2016
Jul 2016
Oct 2016
Jan 2017
Apr 2017
Jul 2017
Oct 2017
Jan 2018
Apr 2018
Jul 2018
Oct 2018
Jan 2019
Apr 2019
Jul 2019
Oct 2019
Jan 2020
Apr 2020
Jul 2020
Oct 2020
Jan 2021
Apr 2021
Jul 2021
Oct 2021
Jan 2022
Apr 2022
Jul 2022
Oct 2022
Jan 2023
Apr 2023
Jul 2023
Oct 2023
Time
Sex
Girls
Boys

(c)

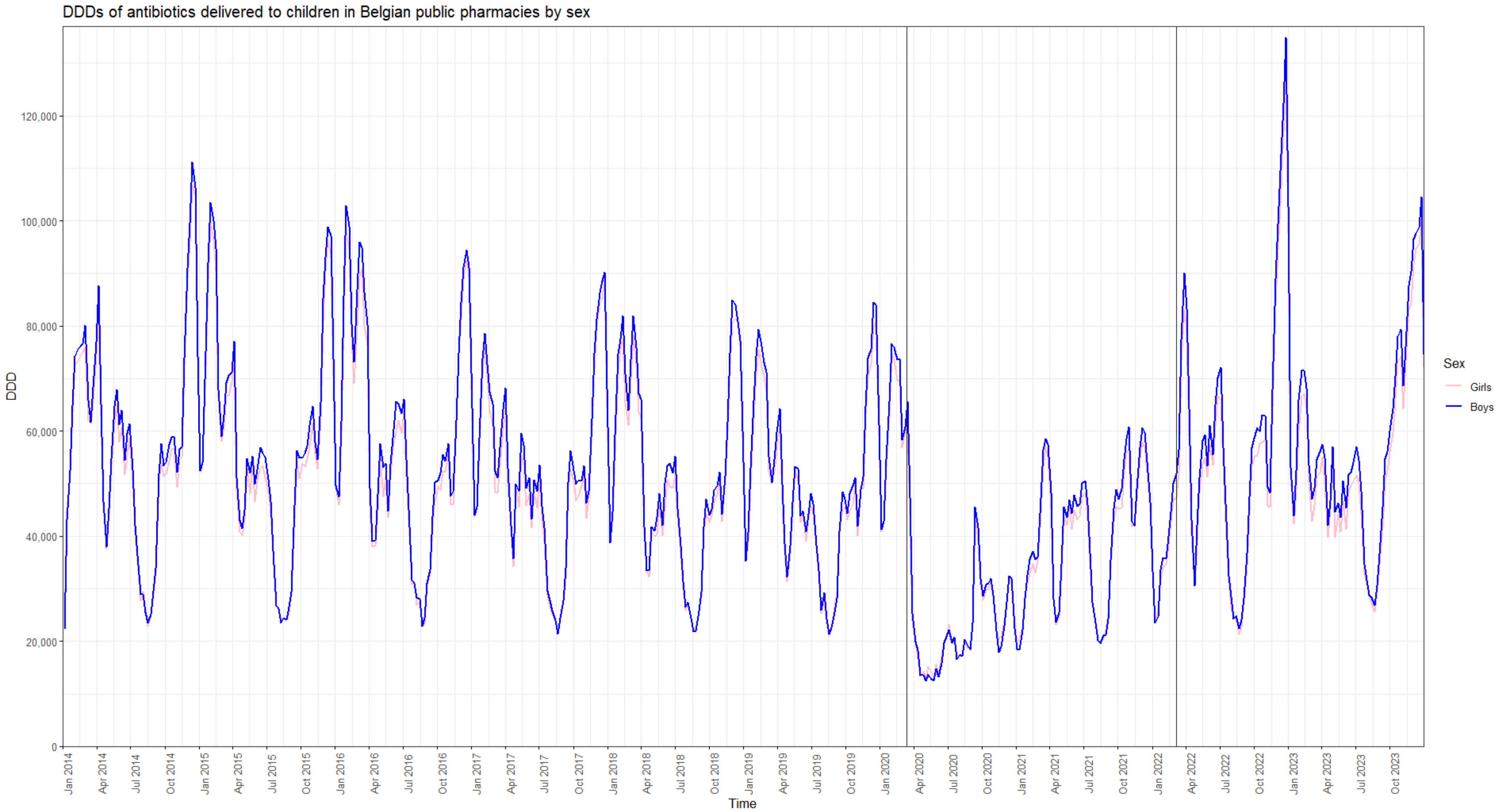

Figure S6. Line chart of antibiotics delivered to children in Belgian public pharmacies by sex, expressed as number of packages (a), healthcare expenditures (b), and Defined Daily Doses (DDDs) (c).

The grey vertical lines represent the start of the COVID-19 pandemic (i.e., the week of 16 March 2020) and the start of the post-COVID period (i.e., the week of 14 March 2022), respectively.

(a)

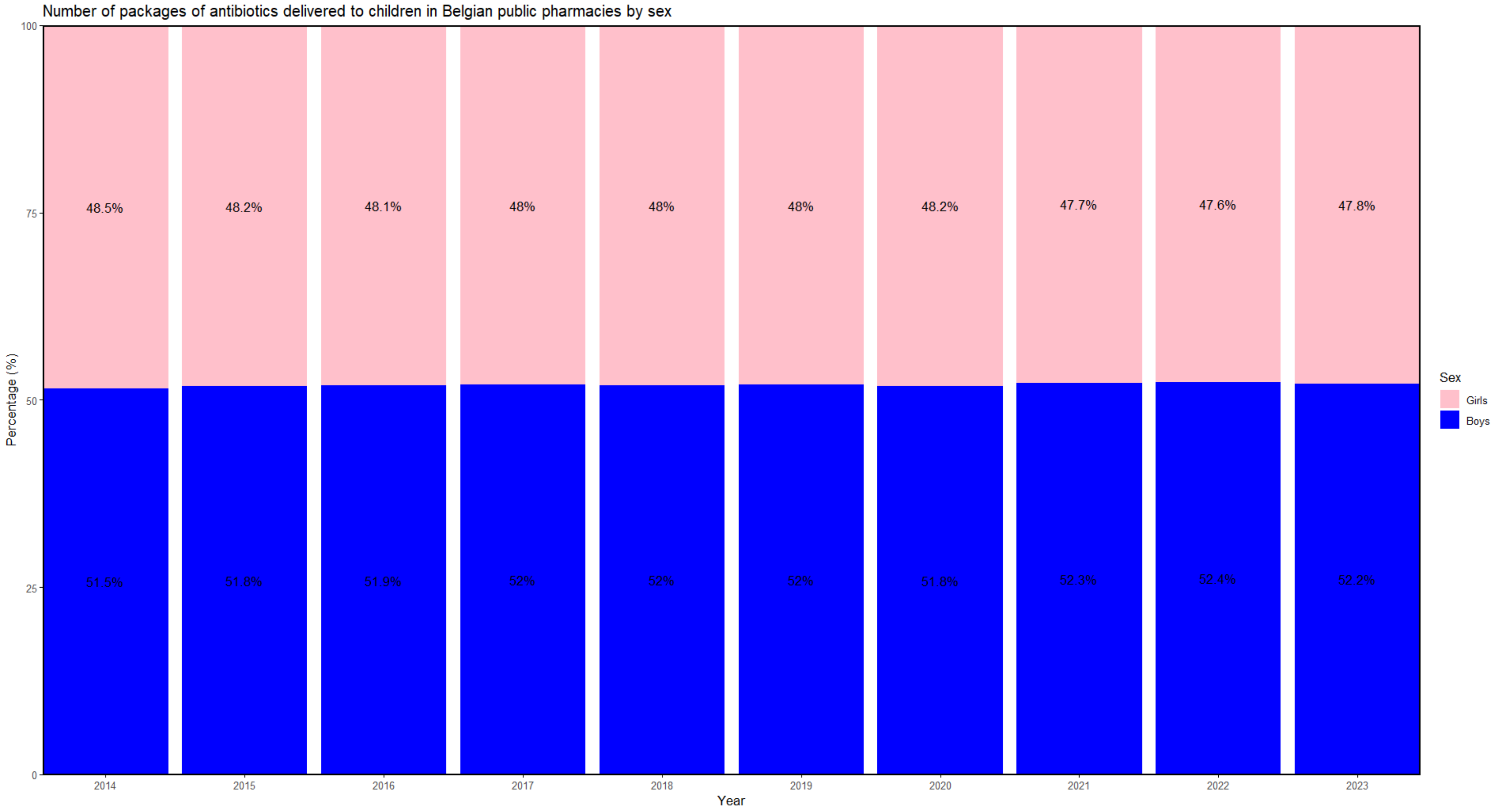
Number of packages of antibiotics delivered to children in Belgian public pharmacies by sex
100
75
50
25
0
Percentage (%)
48.5%
48.2%
48.1%
48%
48%
48%
48.2%
47.7%
47.6%
47.8%
51.5%
51.8%
51.9%
52%
52%
52%
51.8%
52.3%
52.4%
52.2%
2014
2015
2016
2017
2018
2019
2020
2021
2022
2023
Year
Sex
Girls
Boys

(b)

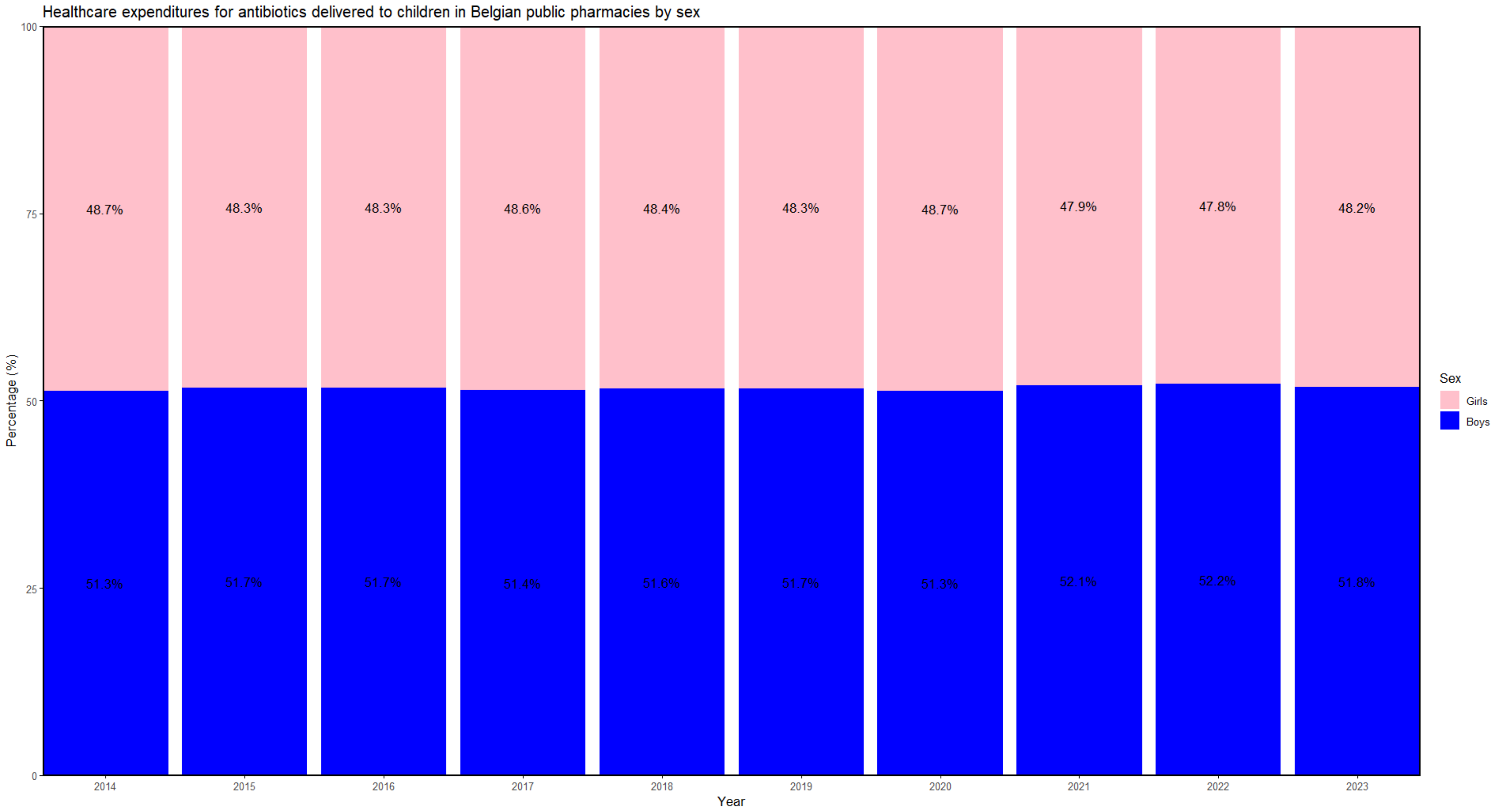
Healthcare expenditures for antibiotics delivered to children in Belgian public pharmacies by sex
Percentage (%)
100
75
50
25
0
48.7%
48.3%
48.3%
48.6%
48.4%
48.3%
48.7%
47.9%
47.8%
48.2%
51.3%
51.7%
51.7%
51.4%
51.6%
51.7%
51.3%
52.1%
52.2%
51.8%
2014
2015
2016
2017
2018
2019
2020
2021
2022
2023
Year
Sex
Girls
Boys

(c)

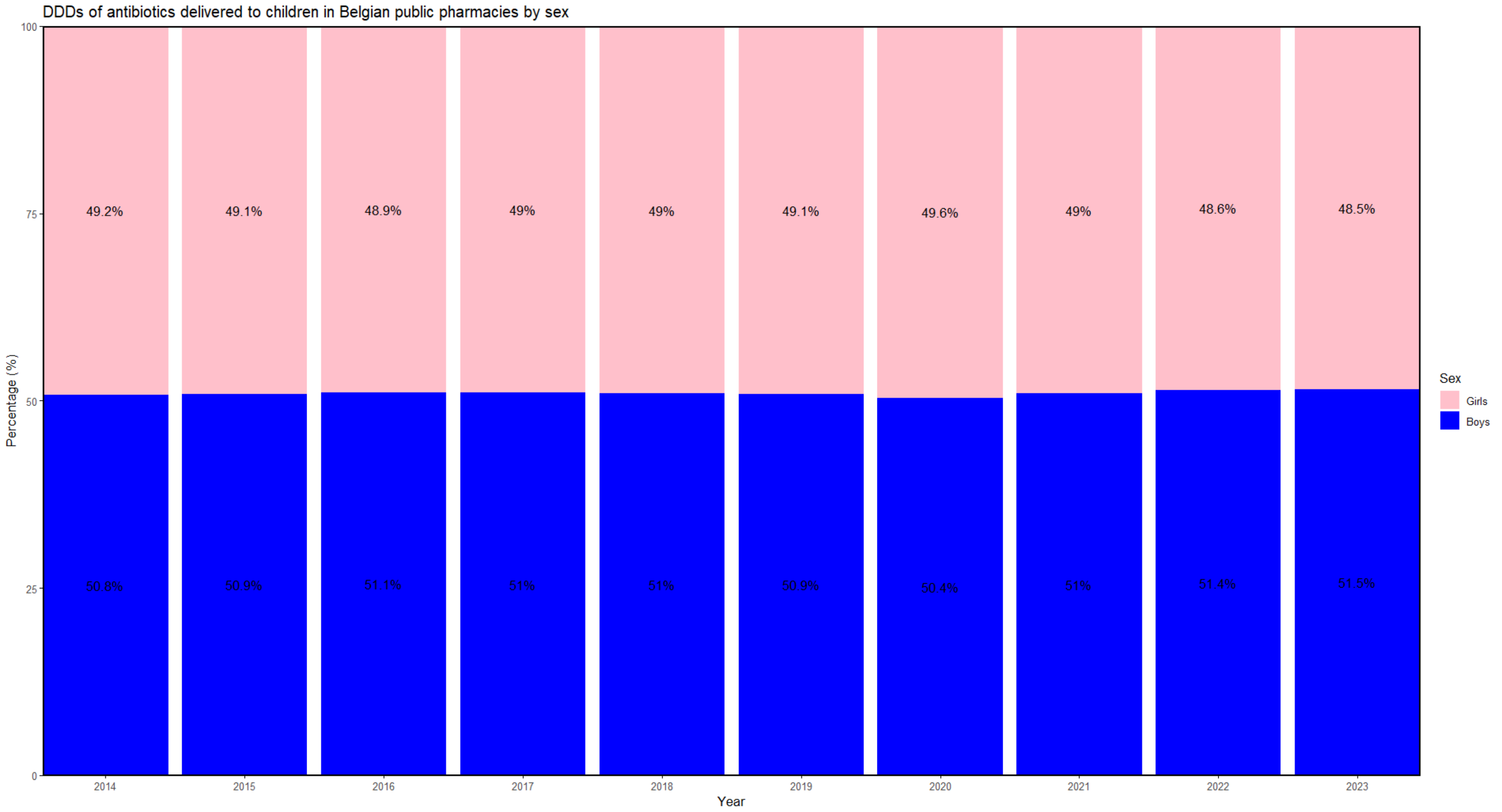

Figure S7. Bar chart of antibiotics delivered to children in Belgian public pharmacies by sex, expressed as number of packages (a), healthcare expenditures (b), and Defined Daily Doses (DDDs) (c).

(a)

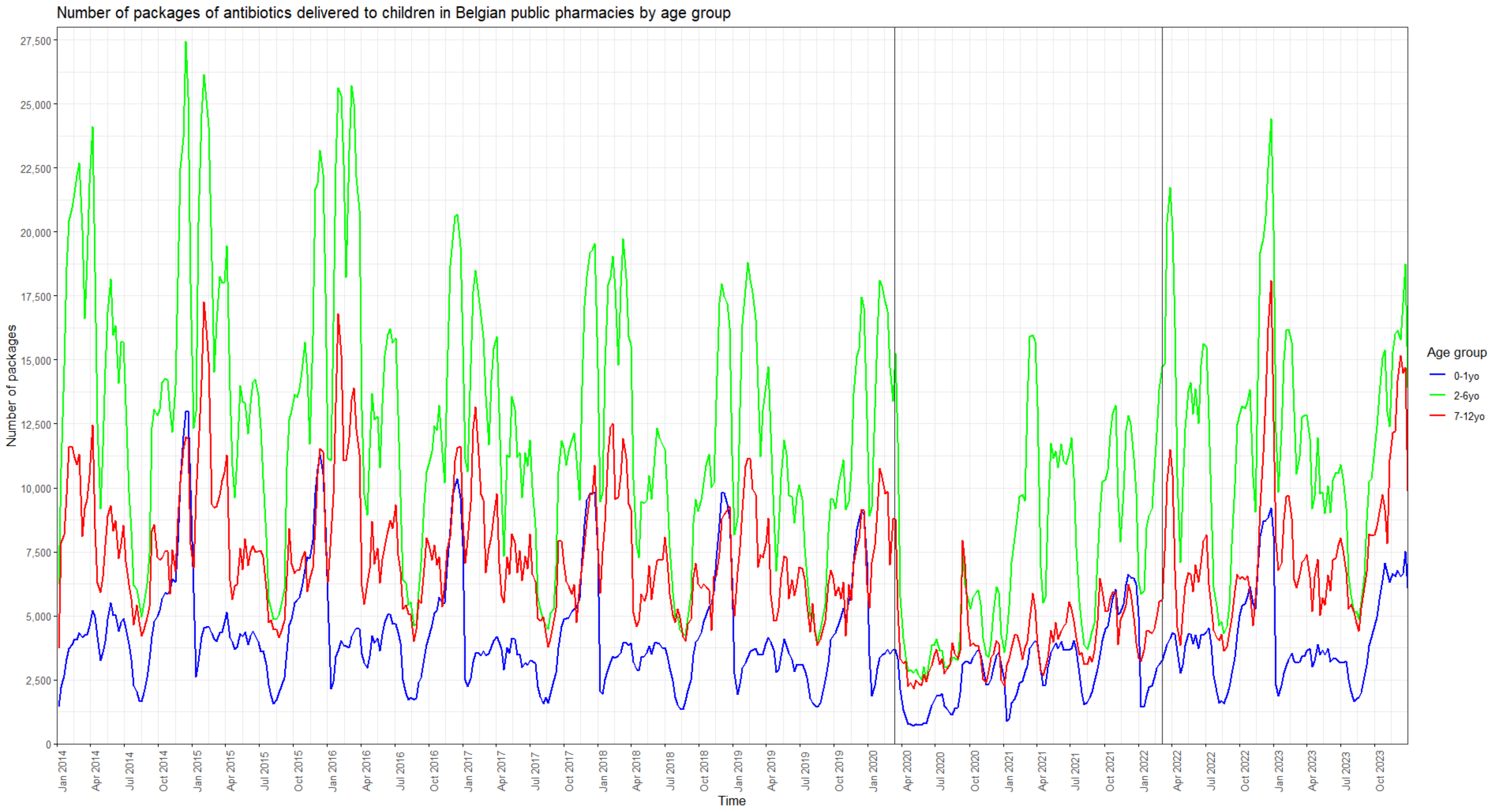
Number of packages of antibiotics delivered to children in Belgian public pharmacies by age group
Number of packages
27,500
25,000
22,500
20,000
17,500
15,000
12,500
10,000
7,500
5,000
2,500
0
Jan 2014
Apr 2014
Jul 2014
Oct 2014
Jan 2015
Apr 2015
Jul 2015
Oct 2015
Jan 2016
Apr 2016
Jul 2016
Oct 2016
Jan 2017
Apr 2017
Jul 2017
Oct 2017
Jan 2018
Apr 2018
Jul 2018
Oct 2018
Jan 2019
Apr 2019
Jul 2019
Oct 2019
Jan 2020
Apr 2020
Jul 2020
Oct 2020
Jan 2021
Apr 2021
Jul 2021
Oct 2021
Jan 2022
Apr 2022
Jul 2022
Oct 2022
Jan 2023
Apr 2023
Jul 2023
Oct 2023
Time
Age group
0-1yo
2-6yo
7-12yo

(b)

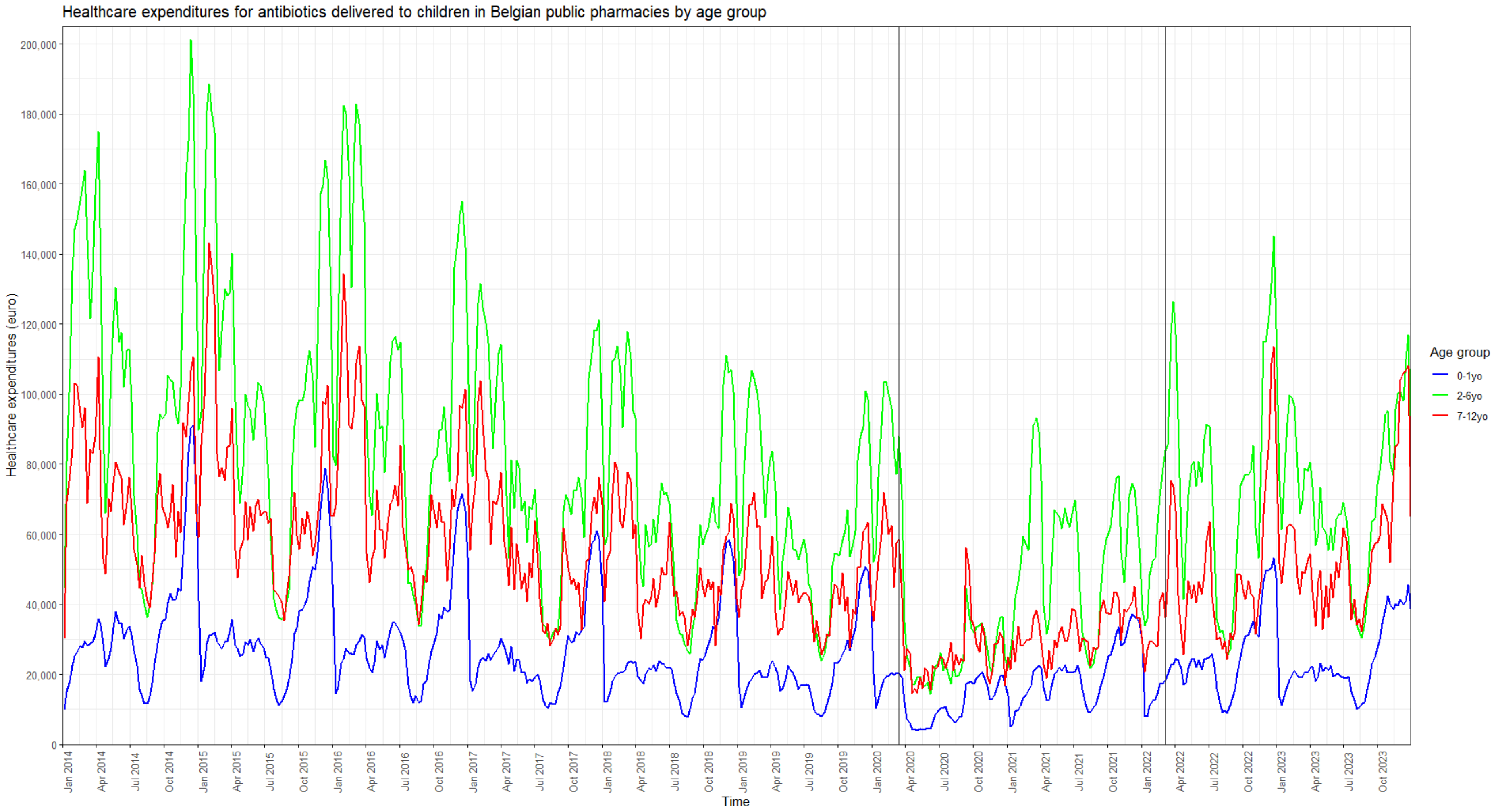

Healthcare expenditures for antibiotics delivered to children in Belgian public pharmacies by age group
Healthcare expenditures (euro)
200,000
180,000
160,000
140,000
120,000
100,000
80,000
60,000
40,000
20,000
0
Jan 2014
Apr 2014
Jul 2014
Oct 2014
Jan 2015
Apr 2015
Jul 2015
Oct 2015
Jan 2016
Apr 2016
Jul 2016
Oct 2016
Jan 2017
Apr 2017
Jul 2017
Oct 2017
Jan 2018
Apr 2018
Jul 2018
Oct 2018
Jan 2019
Apr 2019
Jul 2019
Oct 2019
Jan 2020
Apr 2020
Jul 2020
Oct 2020
Jan 2021
Apr 2021
Jul 2021
Oct 2021
Jan 2022
Apr 2022
Jul 2022
Oct 2022
Jan 2023
Apr 2023
Jul 2023
Oct 2023
Time
Age group
0-1yo
2-6yo
7-12yo

(c)

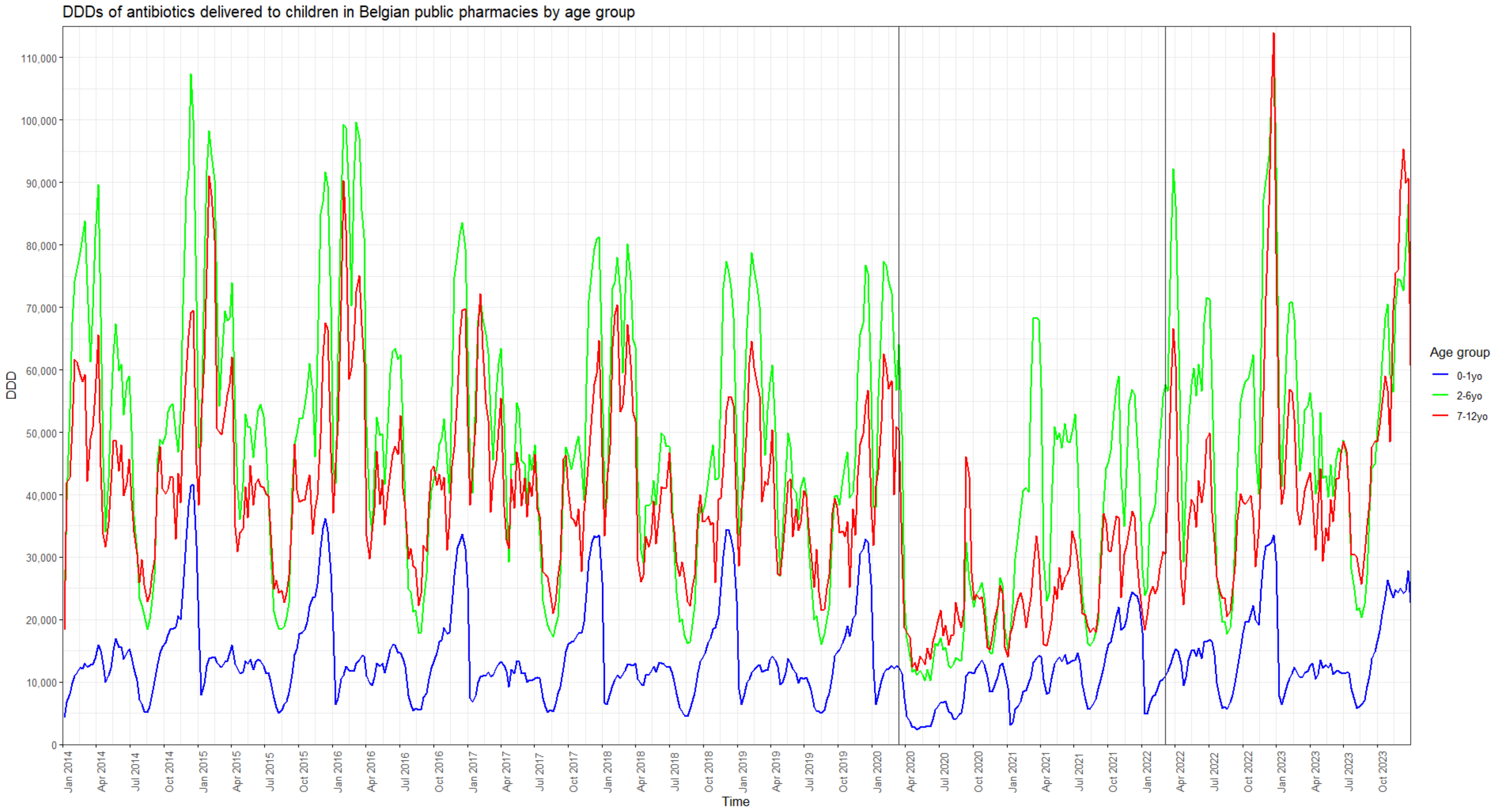

Figure S8. Line chart of antibiotics delivered to children in Belgian public pharmacies by age group, expressed as number of packages (a), healthcare expenditures (b), and Defined Daily Doses (DDDs) (c).

The grey vertical lines represent the start of the COVID-19 pandemic (i.e., the week of 16 March 2020) and the start of the post-COVID period (i.e., the week of 14 March 2022), respectively.

(a)

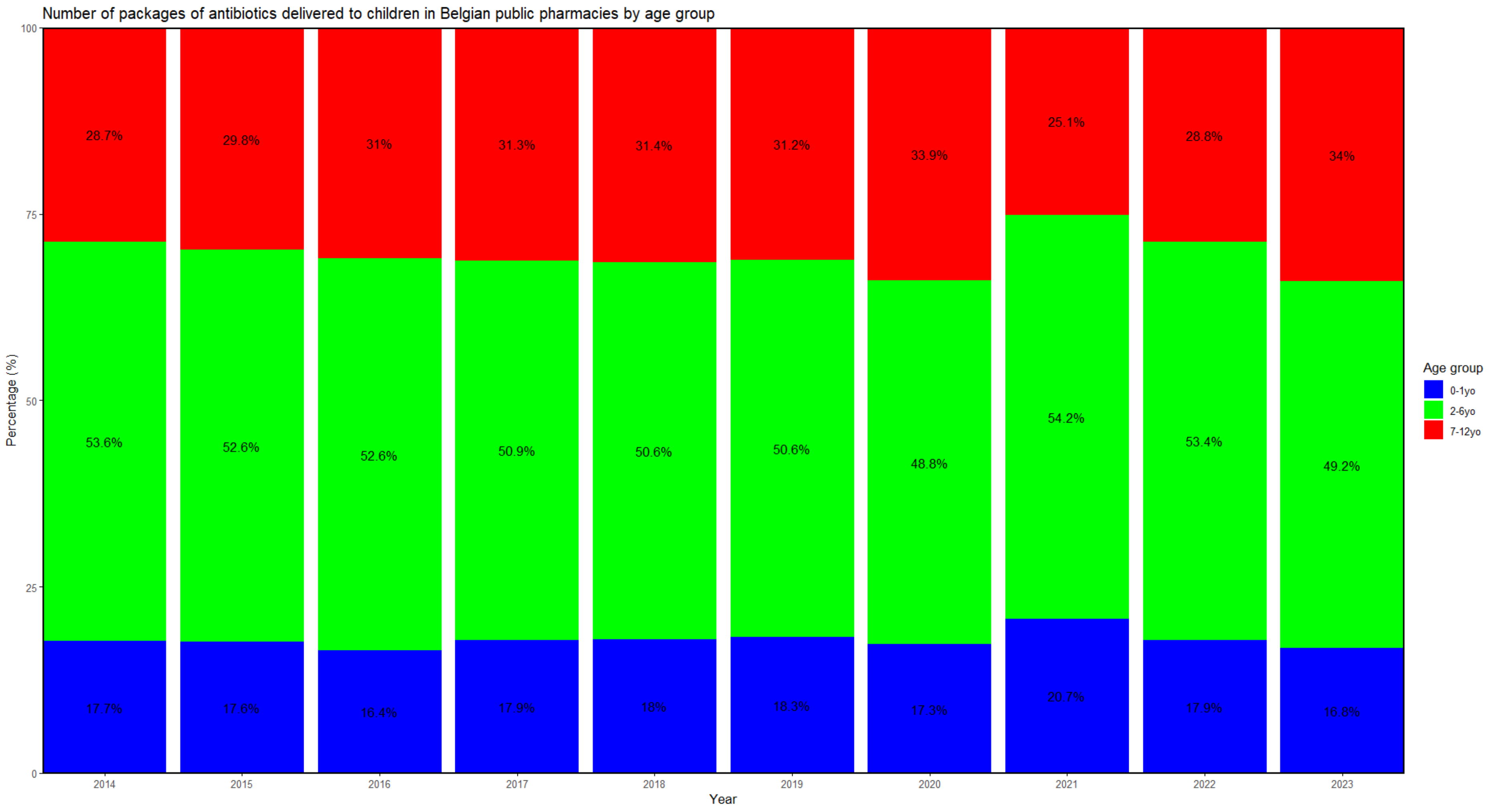
Number of packages of antibiotics delivered to children in Belgian public pharmacies by age group
Percentage (%)
100
75
50
25
0
28.7%
53.6%
17.7%
29.8%
52.6%
17.6%
31%
52.6%
16.4%
31.3%
50.9%
17.9%
31.4%
50.6%
18%
31.2%
50.6%
18.3%
33.9%
48.8%
17.3%
25.1%
54.2%
20.7%
28.8%
53.4%
17.9%
34%
49.2%
16.8%
2014
2015
2016
2017
2018
2019
2020
2021
2022
2023
Year
Age group
0-1yo
2-6yo
7-12yo

(b)

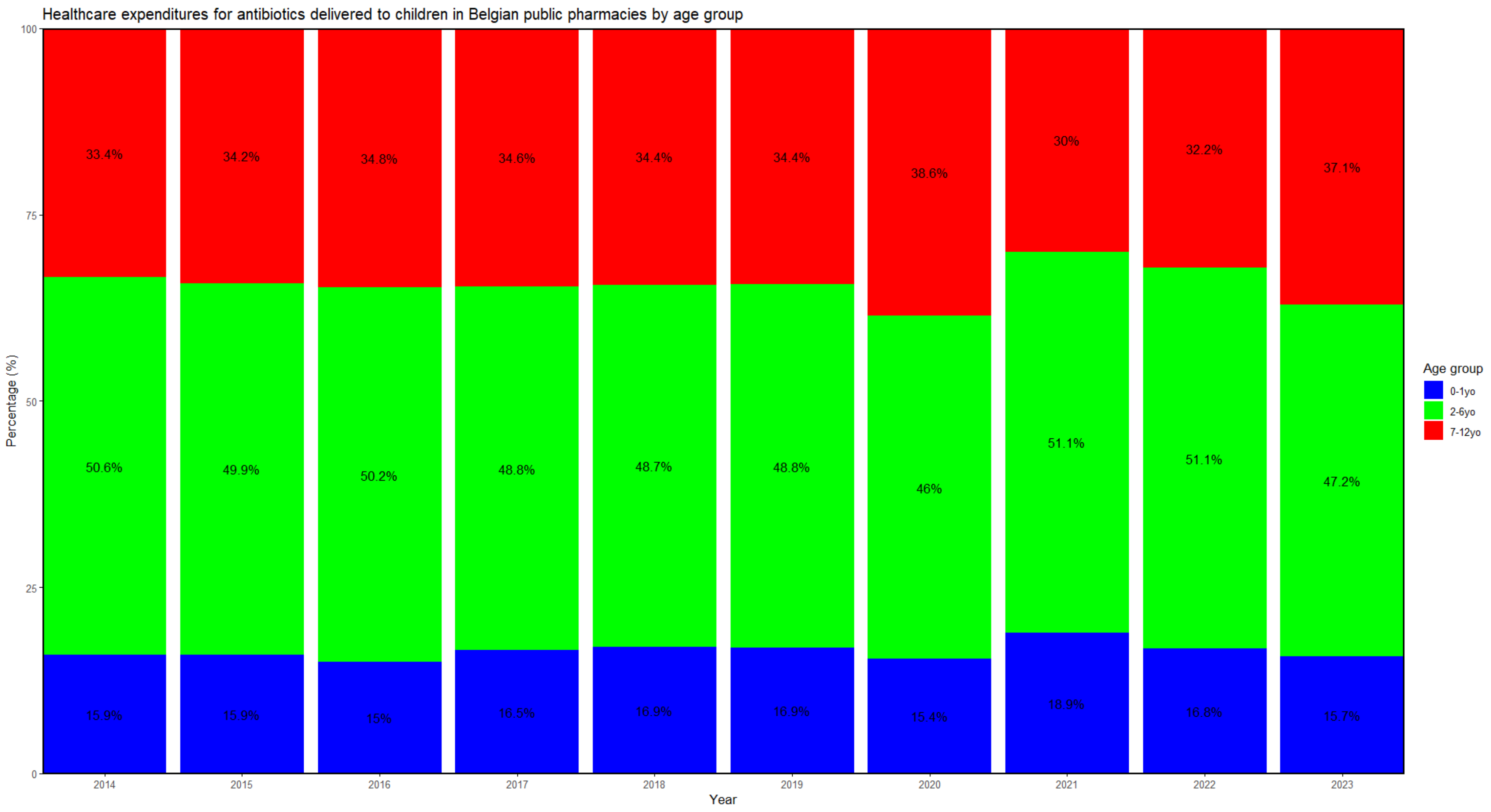
Healthcare expenditures for antibiotics delivered to children in Belgian public pharmacies by age group
Percentage (%)
100
75
50
25
0
33.4%
34.2%
34.8%
34.6%
34.4%
34.4%
38.6%
30%
32.2%
37.1%
50.6%
49.9%
50.2%
48.8%
48.7%
48.8%
46%
51.1%
51.1%
47.2%
15.9%
15.9%
15%
16.5%
16.9%
16.9%
15.4%
18.9%
16.8%
15.7%
2014
2015
2016
2017
2018
2019
2020
2021
2022
2023
Year
Age group
0-1yo
2-6yo
7-12yo

(c)

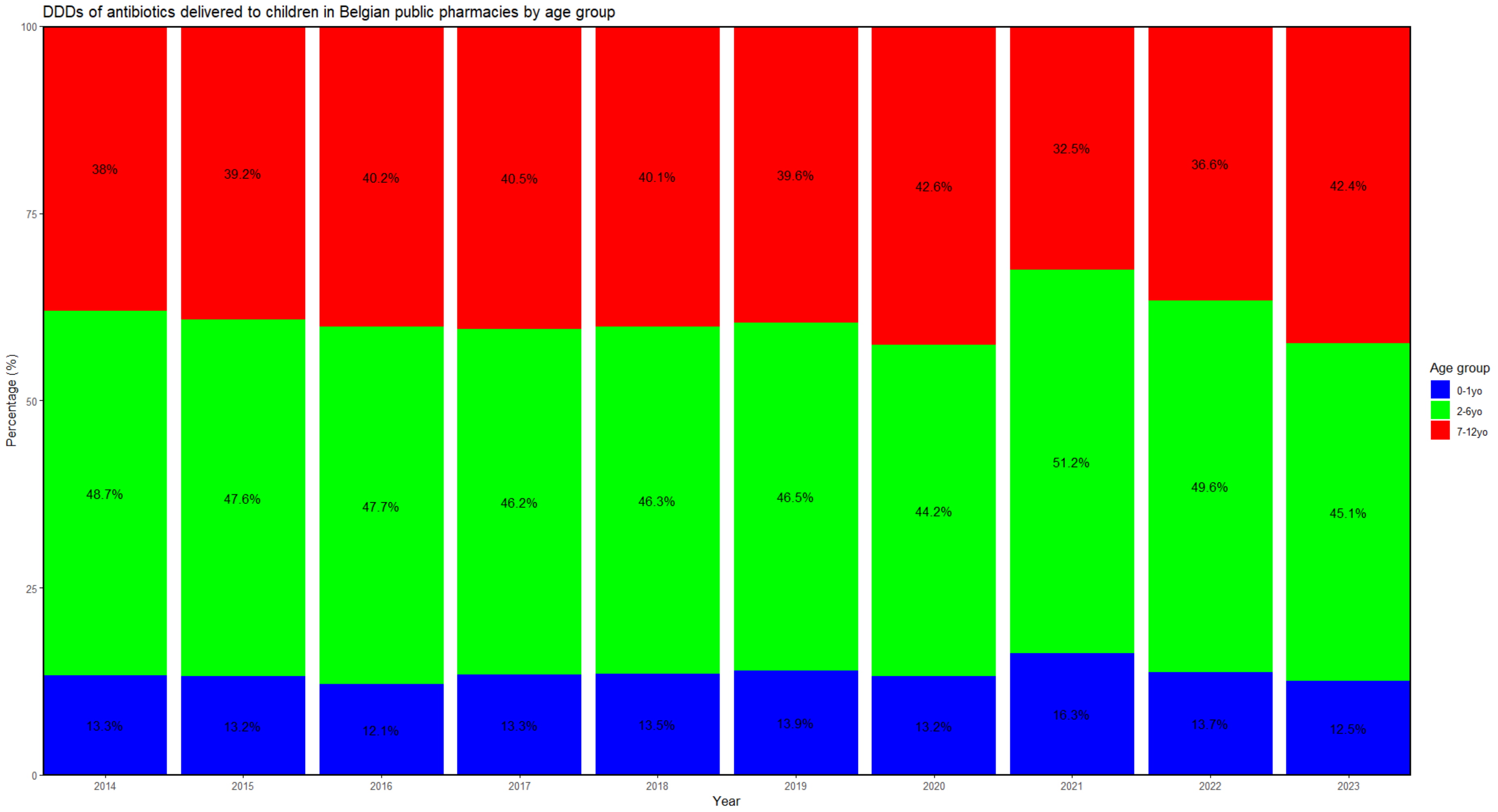

Figure S9. Bar chart of antibiotics delivered to children in Belgian public pharmacies by age group, expressed as number of packages (a), healthcare expenditures (b), and Defined Daily Doses (DDDs) (c).

(a)

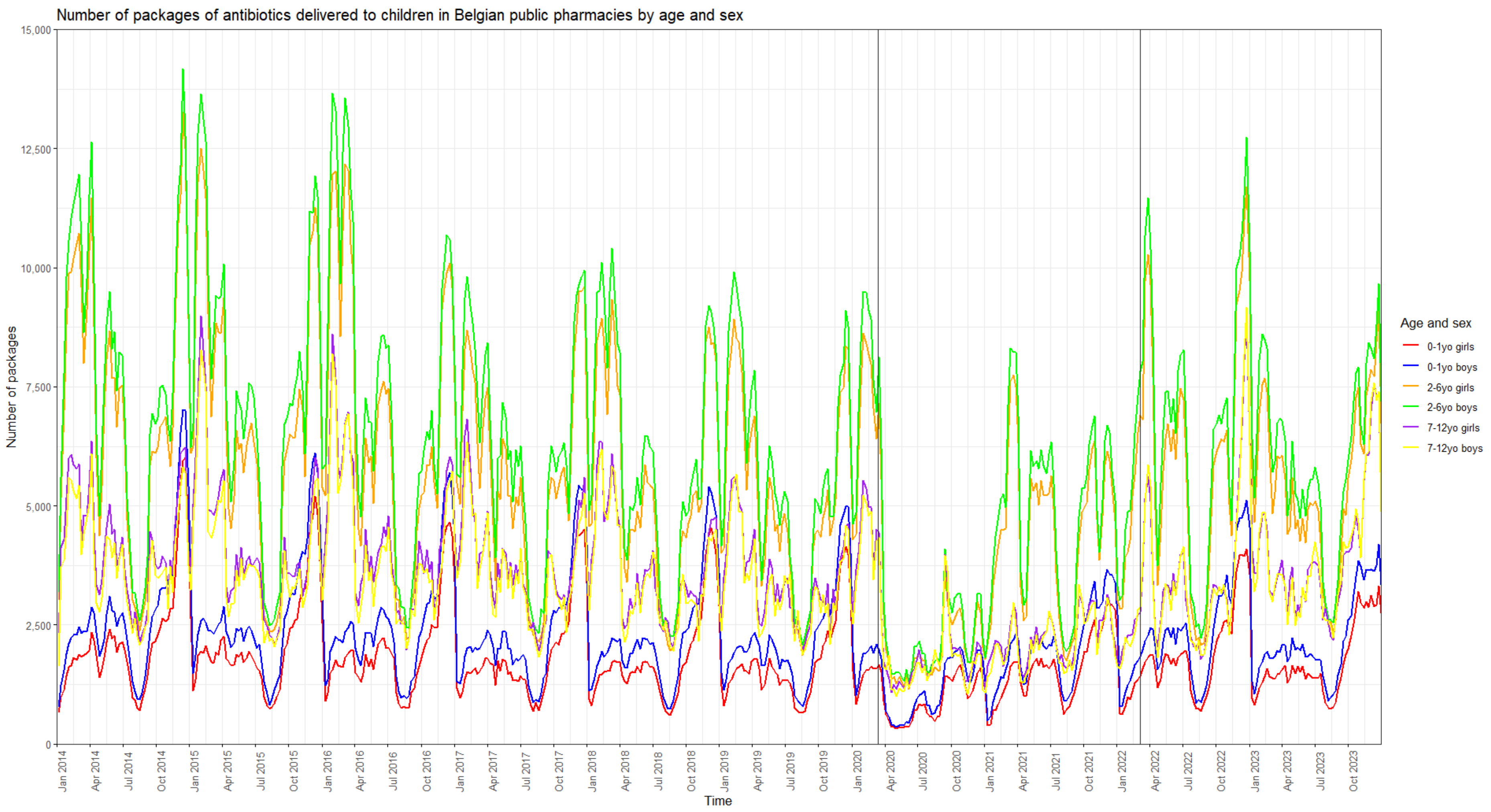
Number of packages of antibiotics delivered to children in Belgian public pharmacies by age and sex
Number of packages
15,000
12,500
10,000
7,500
5,000
2,500
0
Time
Jan 2014
Apr 2014
Jul 2014
Oct 2014
Jan 2015
Apr 2015
Jul 2015
Oct 2015
Jan 2016
Apr 2016
Jul 2016
Oct 2016
Jan 2017
Apr 2017
Jul 2017
Oct 2017
Jan 2018
Apr 2018
Jul 2018
Oct 2018
Jan 2019
Apr 2019
Jul 2019
Oct 2019
Jan 2020
Apr 2020
Jul 2020
Oct 2020
Jan 2021
Apr 2021
Jul 2021
Oct 2021
Jan 2022
Apr 2022
Jul 2022
Oct 2022
Jan 2023
Apr 2023
Jul 2023
Oct 2023
Age and sex
0-1yo girls
0-1yo boys
2-6yo girls
2-6yo boys
7-12yo girls
7-12yo boys

(b)

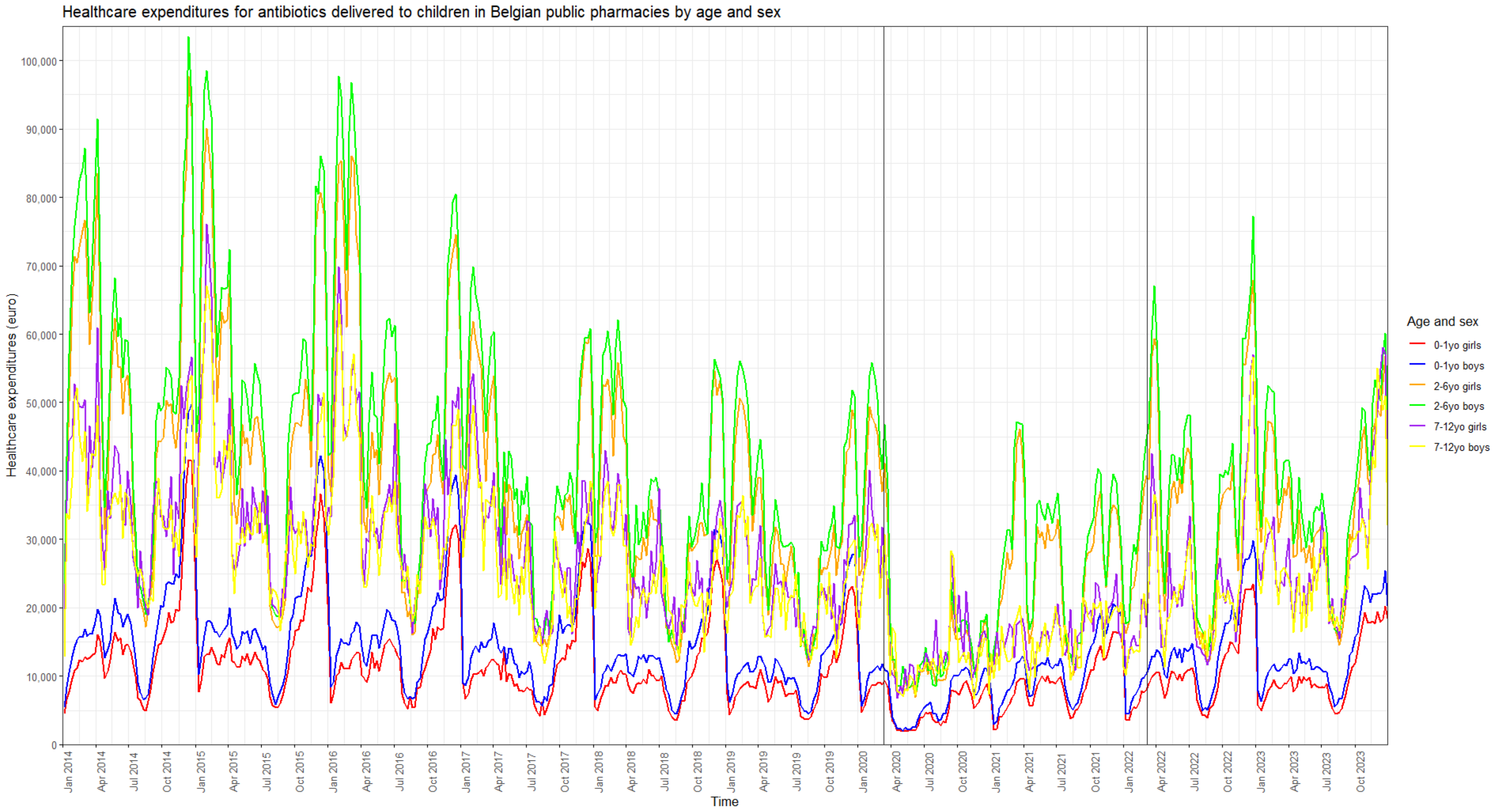

Healthcare expenditures for antibiotics delivered to children in Belgian public pharmacies by age and sex
Healthcare expenditures (euro)
100,000
90,000
80,000
70,000
60,000
50,000
40,000
30,000
20,000
10,000
0
Jan 2014
Apr 2014
Jul 2014
Oct 2014
Jan 2015
Apr 2015
Jul 2015
Oct 2015
Jan 2016
Apr 2016
Jul 2016
Oct 2016
Jan 2017
Apr 2017
Jul 2017
Oct 2017
Jan 2018
Apr 2018
Jul 2018
Oct 2018
Jan 2019
Apr 2019
Jul 2019
Oct 2019
Jan 2020
Apr 2020
Jul 2020
Oct 2020
Jan 2021
Apr 2021
Jul 2021
Oct 2021
Jan 2022
Apr 2022
Jul 2022
Oct 2022
Jan 2023
Apr 2023
Jul 2023
Oct 2023
Time
Age and sex
0-1yo girls
0-1yo boys
2-6yo girls
2-6yo boys
7-12yo girls
7-12yo boys

(c)

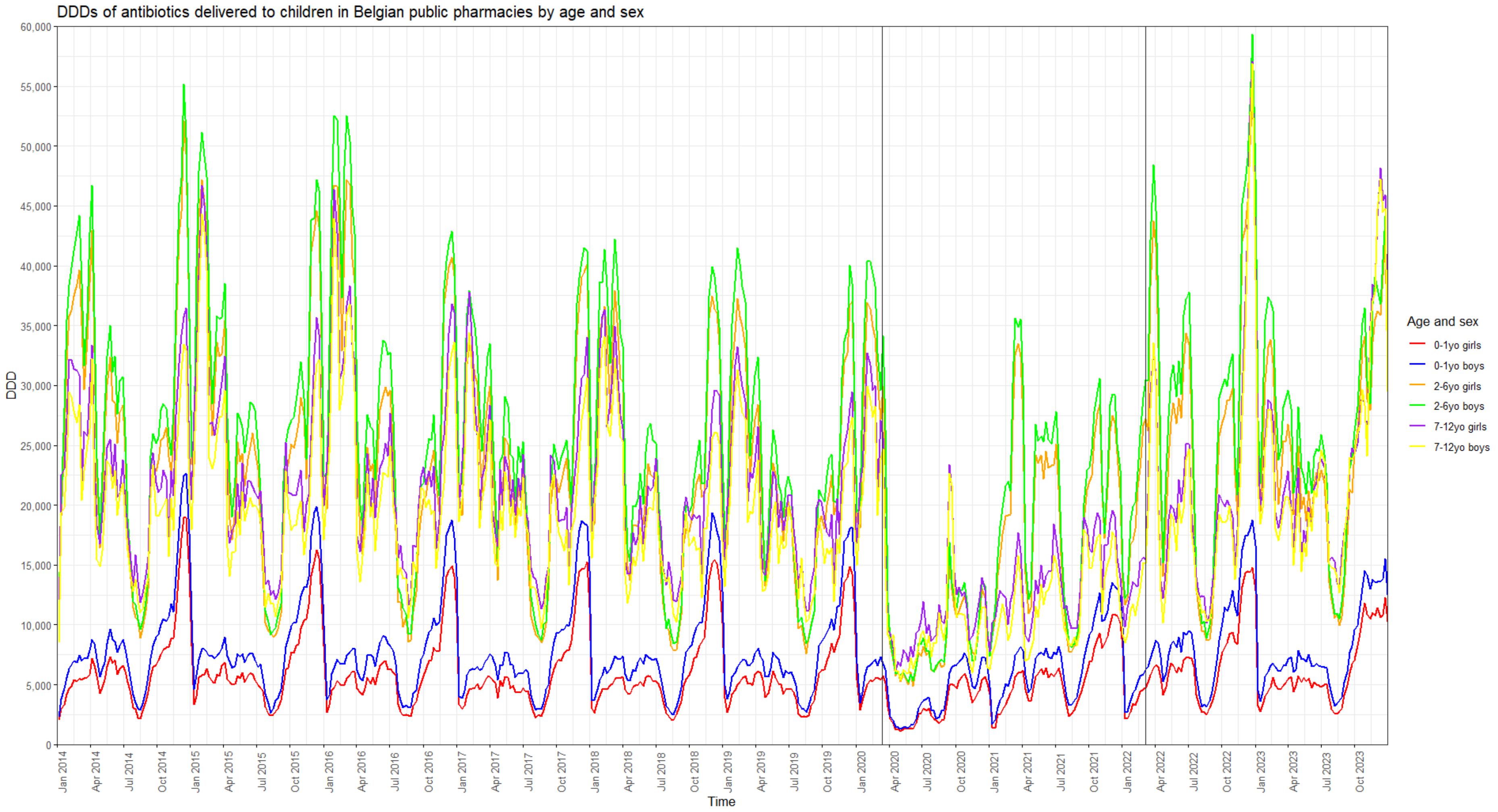

Figure S10. Line chart of antibiotics delivered to children in Belgian public pharmacies by age and sex, expressed as number of packages (a), healthcare expenditures (b), and Defined Daily Doses (DDDs) (c).
The grey vertical lines represent the start of the COVID-19 pandemic (i.e., the week of 16 March 2020) and the start of the post-COVID period (i.e., the week of 14 March 2022), respectively.

(a)

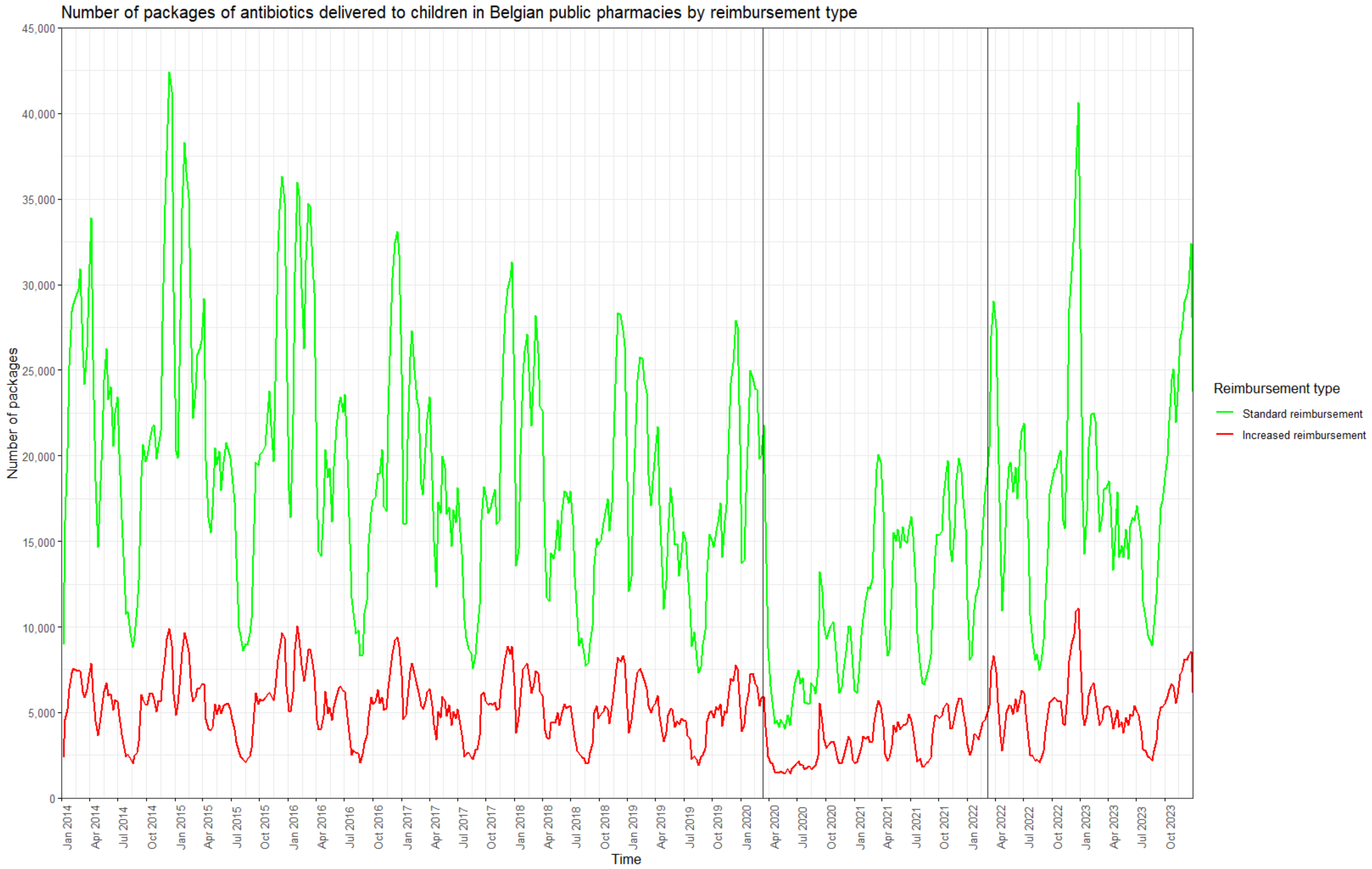
Number of packages of antibiotics delivered to children in Belgian public pharmacies by reimbursement type
Number of packages
45,000
40,000
35,000
30,000
25,000
20,000
15,000
10,000
5,000
0
Jan 2014
Apr 2014
Jul 2014
Oct 2014
Jan 2015
Apr 2015
Jul 2015
Oct 2015
Jan 2016
Apr 2016
Jul 2016
Oct 2016
Jan 2017
Apr 2017
Jul 2017
Oct 2017
Jan 2018
Apr 2018
Jul 2018
Oct 2018
Jan 2019
Apr 2019
Jul 2019
Oct 2019
Jan 2020
Apr 2020
Jul 2020
Oct 2020
Jan 2021
Apr 2021
Jul 2021
Oct 2021
Jan 2022
Apr 2022
Jul 2022
Oct 2022
Jan 2023
Apr 2023
Jul 2023
Oct 2023
Time
Reimbursement type
Standard reimbursement
Increased reimbursement

(b)

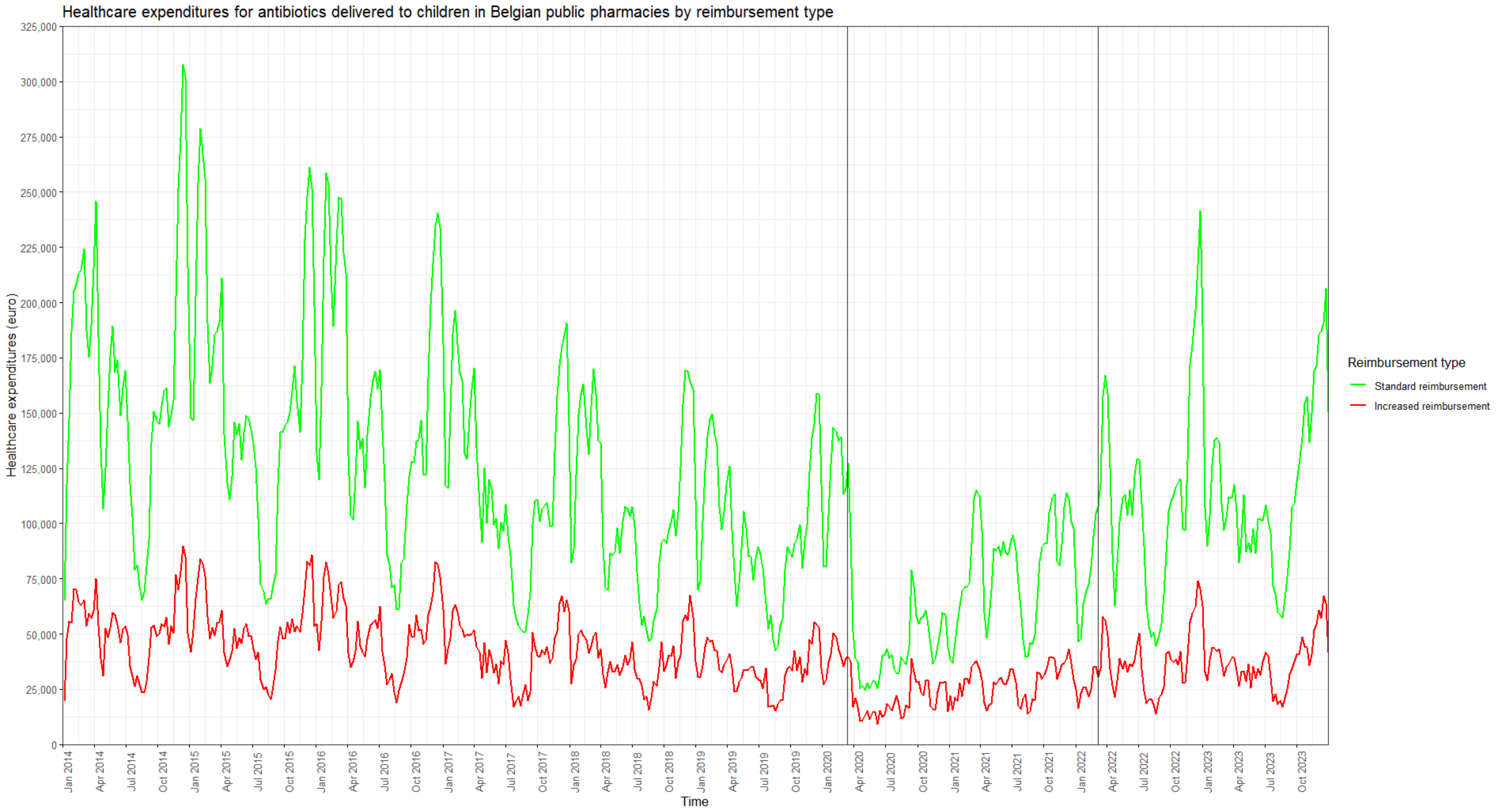

Healthcare expenditures for antibiotics delivered to children in Belgian public pharmacies by reimbursement type
Healthcare expenditures (euro)
325,000
300,000
275,000
250,000
225,000
200,000
175,000
150,000
125,000
100,000
75,000
50,000
25,000
0
Jan 2014
Apr 2014
Jul 2014
Oct 2014
Jan 2015
Apr 2015
Jul 2015
Oct 2015
Jan 2016
Apr 2016
Jul 2016
Oct 2016
Jan 2017
Apr 2017
Jul 2017
Oct 2017
Jan 2018
Apr 2018
Jul 2018
Oct 2018
Jan 2019
Apr 2019
Jul 2019
Oct 2019
Jan 2020
Apr 2020
Jul 2020
Oct 2020
Jan 2021
Apr 2021
Jul 2021
Oct 2021
Jan 2022
Apr 2022
Jul 2022
Oct 2022
Jan 2023
Apr 2023
Jul 2023
Oct 2023
Time
Reimbursement type
Standard reimbursement
Increased reimbursement

(c)

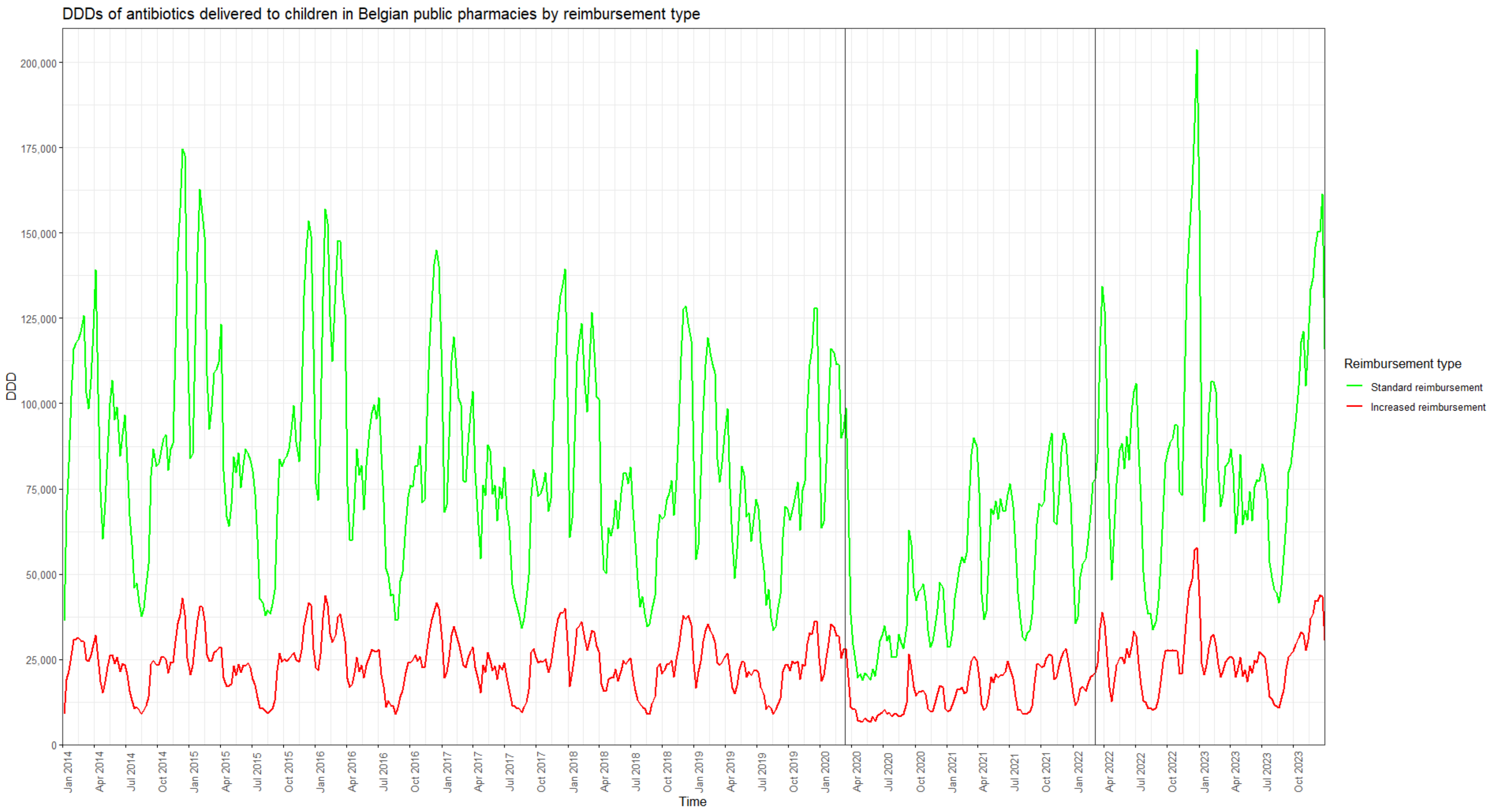

Figure S11. Line chart of antibiotics delivered to children in Belgian public pharmacies by reimbursement type, expressed as number of packages (a), healthcare expenditures (b), and Defined Daily Doses (DDDs) (c).

The grey vertical lines represent the start of the COVID-19 pandemic (i.e., the week of 16 March 2020) and the start of the post-COVID period (i.e., the week of 14 March 2022), respectively.

(a)

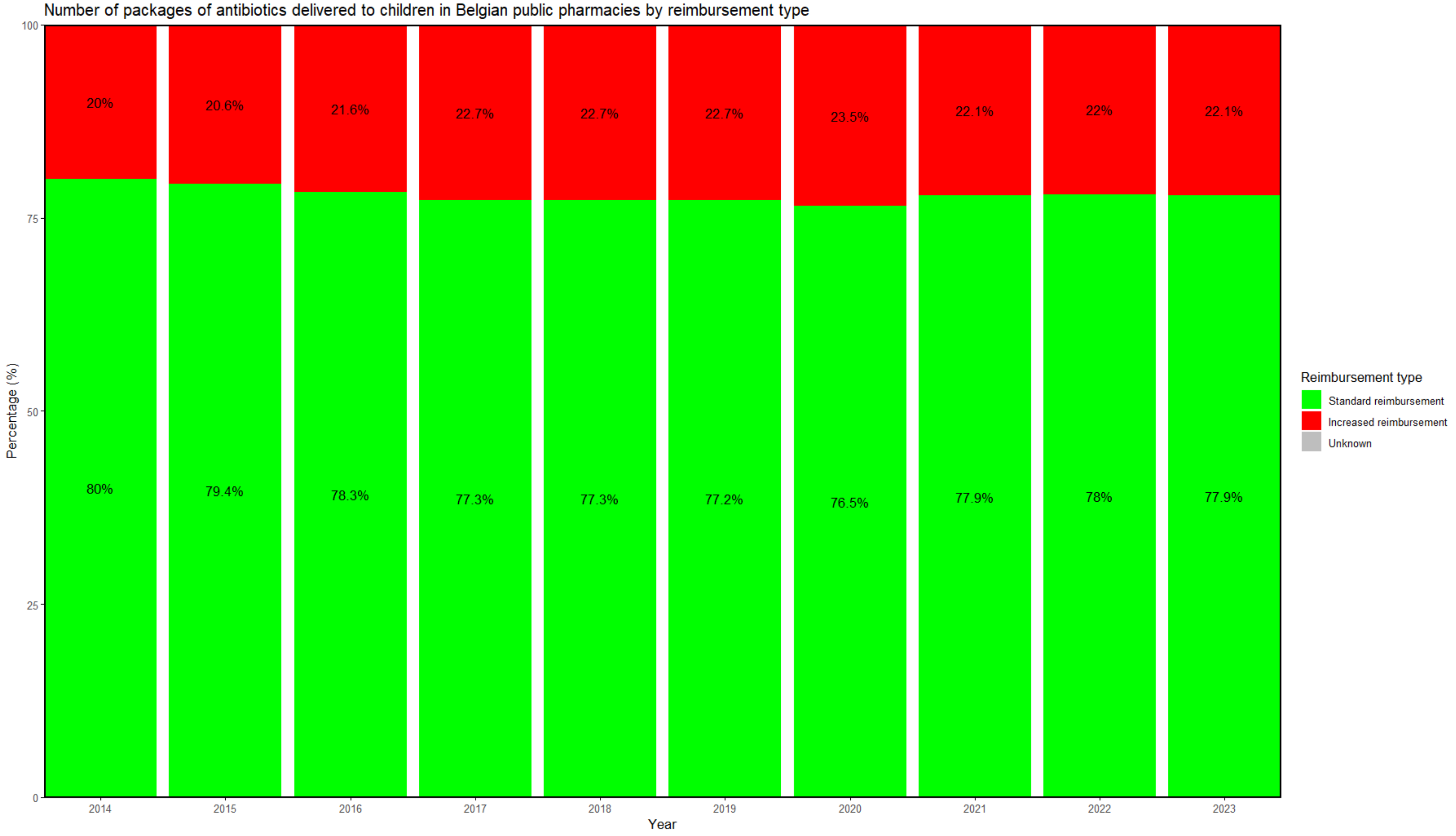
Number of packages of antibiotics delivered to children in Belgian public pharmacies by reimbursement type
100
75
50
25
0
Percentage (%)
20%
20.6%
21.6%
22.7%
22.7%
22.7%
23.5%
22.1%
22%
22.1%
80%
79.4%
78.3%
77.3%
77.3%
77.2%
76.5%
77.9%
78%
77.9%
2014
2015
2016
2017
2018
2019
2020
2021
2022
2023
Year
Reimbursement type
Standard reimbursement
Increased reimbursement
Unknown

(b)

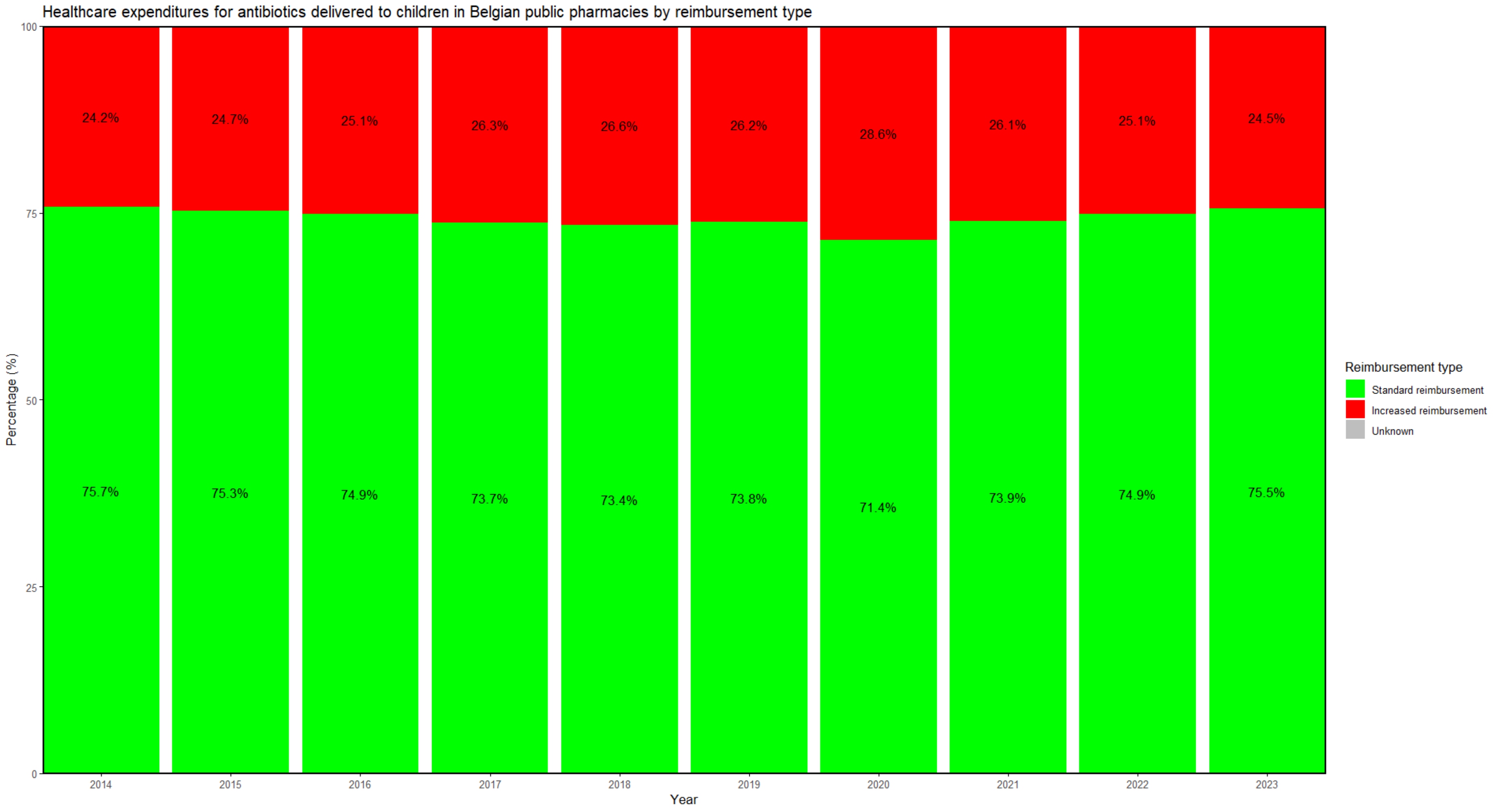
Healthcare expenditures for antibiotics delivered to children in Belgian public pharmacies by reimbursement type
Percentage (%)
100
75
50
25
0
24.2%
24.7%
25.1%
26.3%
26.6%
26.2%
28.6%
26.1%
25.1%
24.5%
75.7%
75.3%
74.9%
73.7%
73.4%
73.8%
71.4%
73.9%
74.9%
75.5%
2014
2015
2016
2017
2018
2019
2020
2021
2022
2023
Year
Reimbursement type
Standard reimbursement
Increased reimbursement
Unknown

(c)

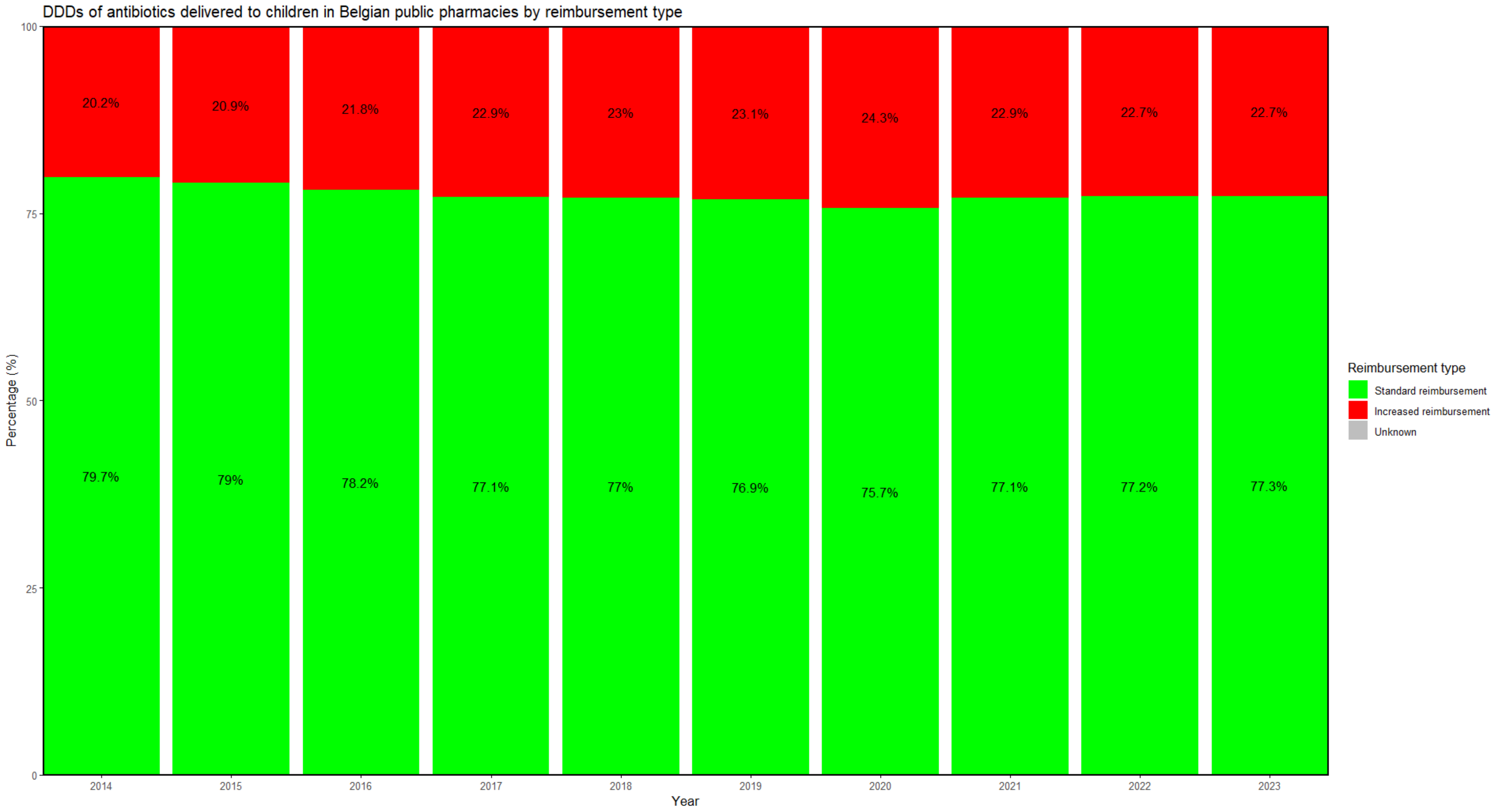

Figure S12. Bar chart of antibiotics delivered to children in Belgian public pharmacies by reimbursement type, expressed as number of packages (a), healthcare expenditures (b), and Defined Daily Doses (DDDs) (c).

0.019% of the prescriptions has missing data on the reimbursement type (i.e., ‘Unknown’).

SPECIALTY OF THE PRESCRIBER

Table S11. Relative change of number of packages, healthcare expenditures and Defined Daily Doses (DDDs) by specialty, per period (standardised per week).

	Number of packages	Healthcare expenditures	DDDs
During vs before COVID			
General practitioner	- 46.6%	- 53.9%	- 43.9%
Specialist	- 35.3%	- 40.6%	- 31.1%
Paediatrician	- 35.7%	- 40.4%	- 31.7%
Emergency physician	- 25.0%	- 36.2%	- 19.6%
Ear/nose/throat physician	- 45.3%	- 51.8%	- 43.4%
Dermatologist	- 20.3%	- 33.8%	- 10.4%
Dentist	- 18.3%	- 31.1%	- 9.17%
After vs during COVID			
General practitioner	+ 77.2%	+ 85.9%	+ 88.1%
Specialist	+ 54.2%	+ 47.3%	+ 52.4%
Paediatrician	+ 55.2%	+ 46.0%	+ 54.5%
Emergency physician	+ 80.6%	+ 99.8%	+ 79.7%
Ear/nose/throat physician	+ 77.6%	+ 80.9%	+ 78.9%
Dermatologist	+ 6.75%	+ 7.50%	- 8.96%
Dentist	- 1.52%	+ 3.93%	+ 2.35%

(a)

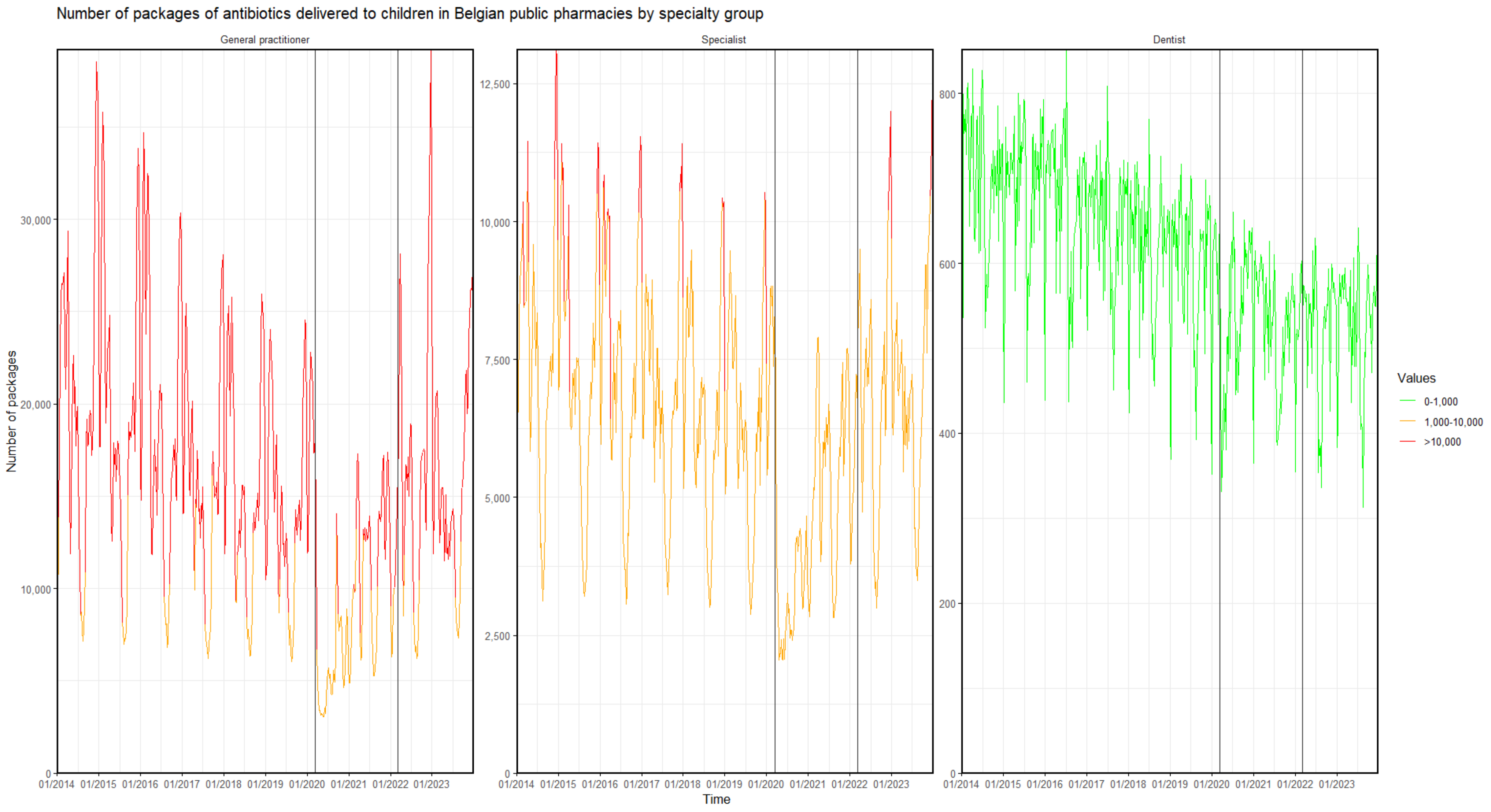
Number of packages of antibiotics delivered to children in Belgian public pharmacies by specialty group
General practitioner
Specialist
Dentist
Number of packages
Time
Values
0-1,000
1,000-10,000
>10,000
0
10,000
20,000
30,000
2,500
5,000
7,500
10,000
12,500
200
400
600
800
01/2014
01/2015
01/2016
01/2017
01/2018
01/2019
01/2020
01/2021
01/2022
01/2023

(b)

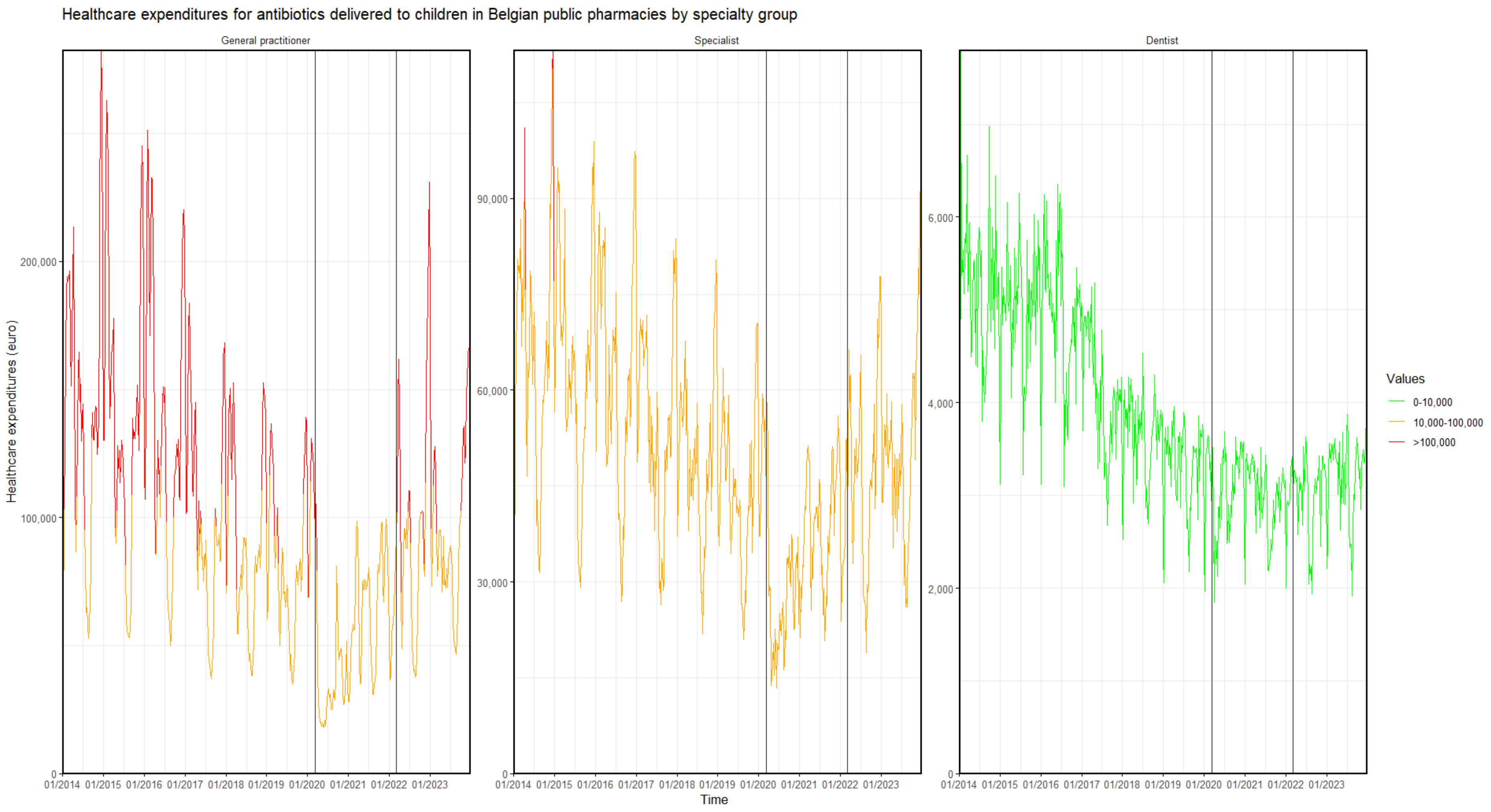
Healthcare expenditures for antibiotics delivered to children in Belgian public pharmacies by specialty group
General practitioner
Specialist
Dentist
Healthcare expenditures (euro)
200,000
100,000
0
90,000
60,000
30,000
6,000
4,000
2,000
01/2014 01/2015 01/2016 01/2017 01/2018 01/2019 01/2020 01/2021 01/2022 01/2023
Time
Values
0-10,000
10,000-100,000
>100,000

(c)

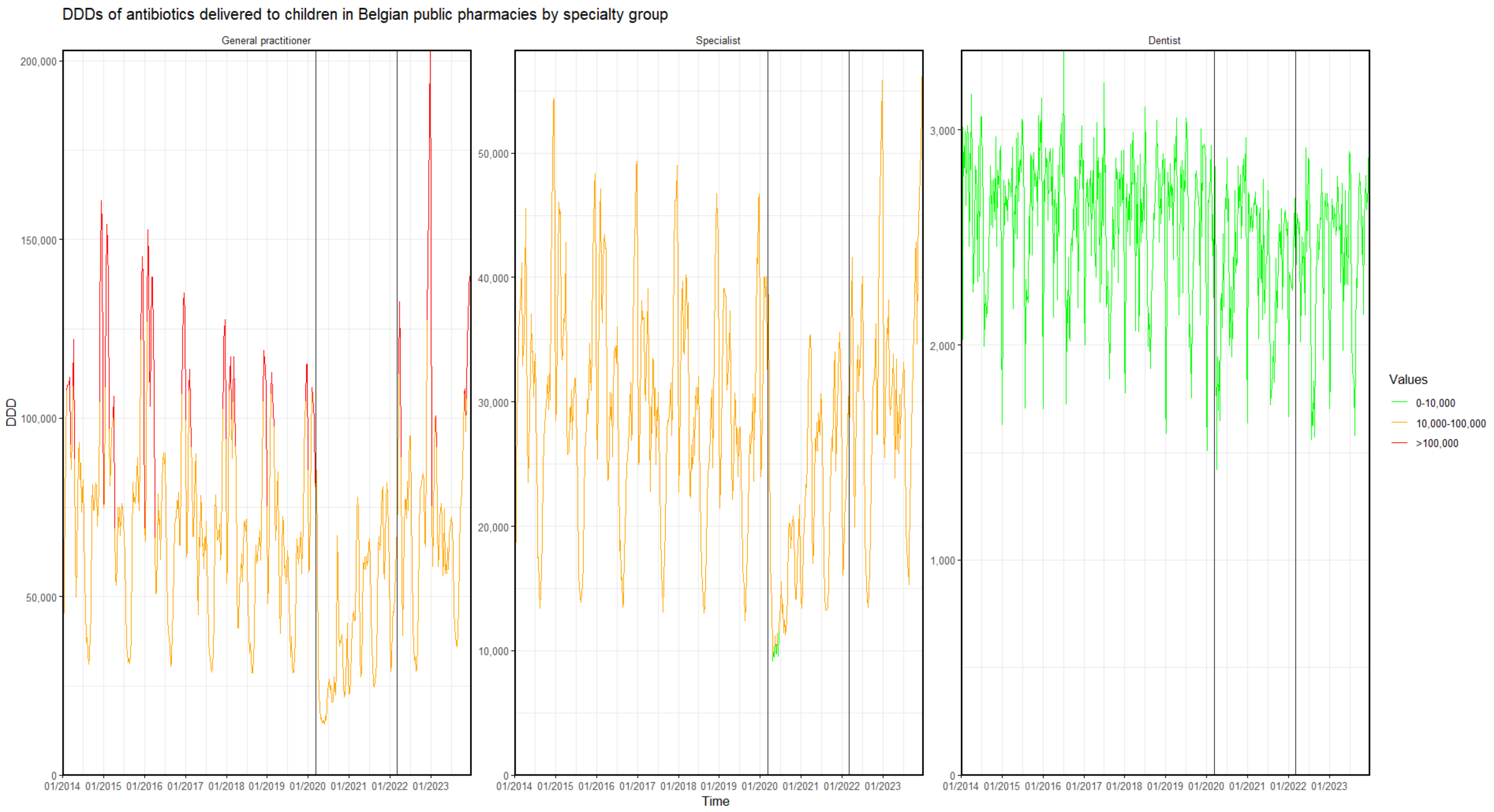

Figure S13. Line chart of antibiotics delivered to children in Belgian public pharmacies by specialty group, expressed as number of packages (a), healthcare expenditures (b), and Defined Daily Doses (DDDs) (c).
The grey vertical lines represent the start of the COVID-19 pandemic (i.e., the week of 16 March 2020) and the start of the post-COVID period (i.e., the week of 14 March 2022), respectively.

(a)

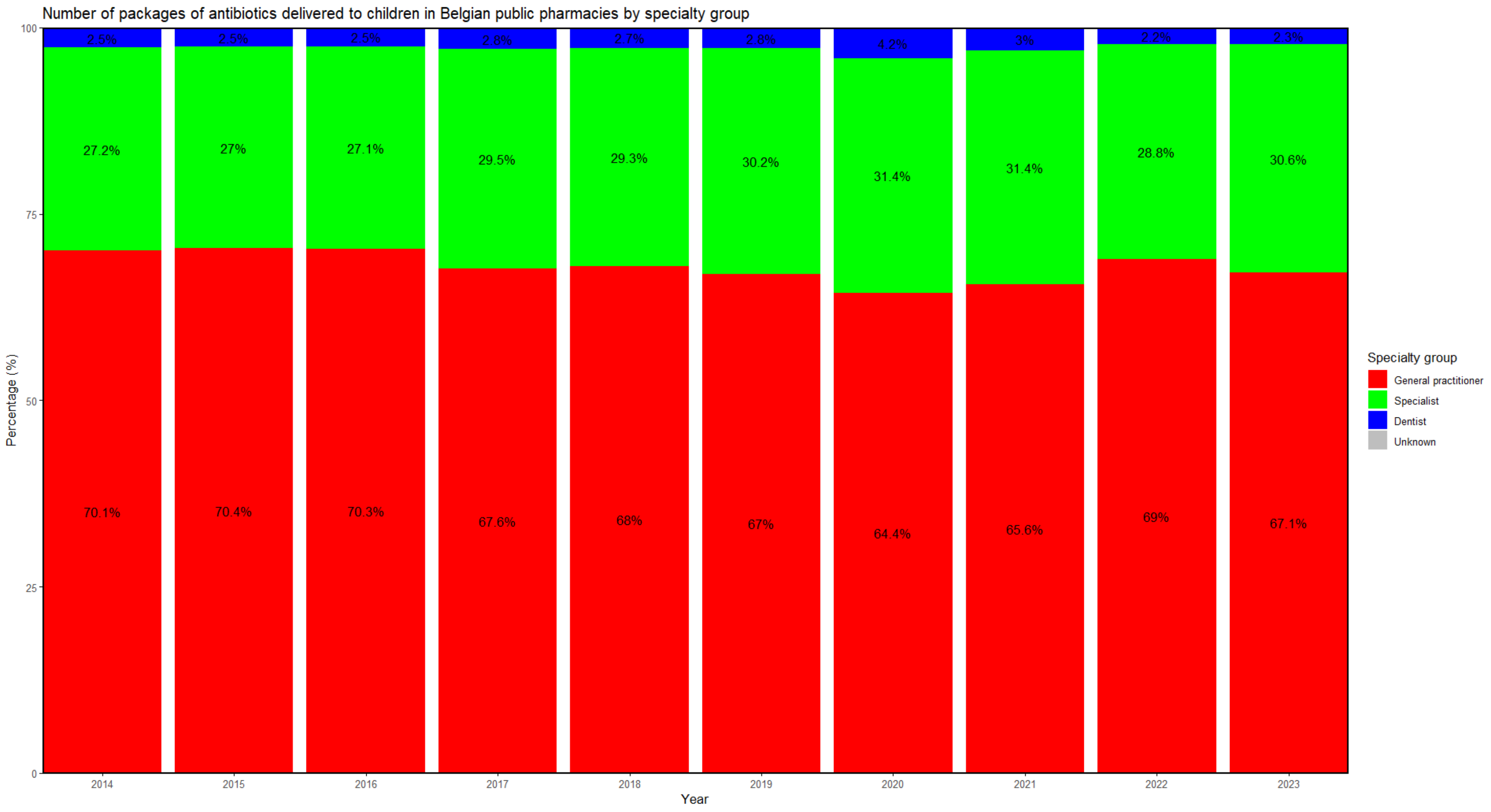
Number of packages of antibiotics delivered to children in Belgian public pharmacies by specialty group
Percentage (%)
100
75
50
25
0
2.5%
2.5%
2.5%
2.8%
2.7%
2.8%
4.2%
3%
2.2%
2.3%
27.2%
27%
27.1%
29.5%
29.3%
30.2%
31.4%
31.4%
28.8%
30.6%
70.1%
70.4%
70.3%
67.6%
68%
67%
64.4%
65.6%
69%
67.1%
2014
2015
2016
2017
2018
2019
2020
2021
2022
2023
Year
Specialty group
General practitioner
Specialist
Dentist
Unknown

(b)

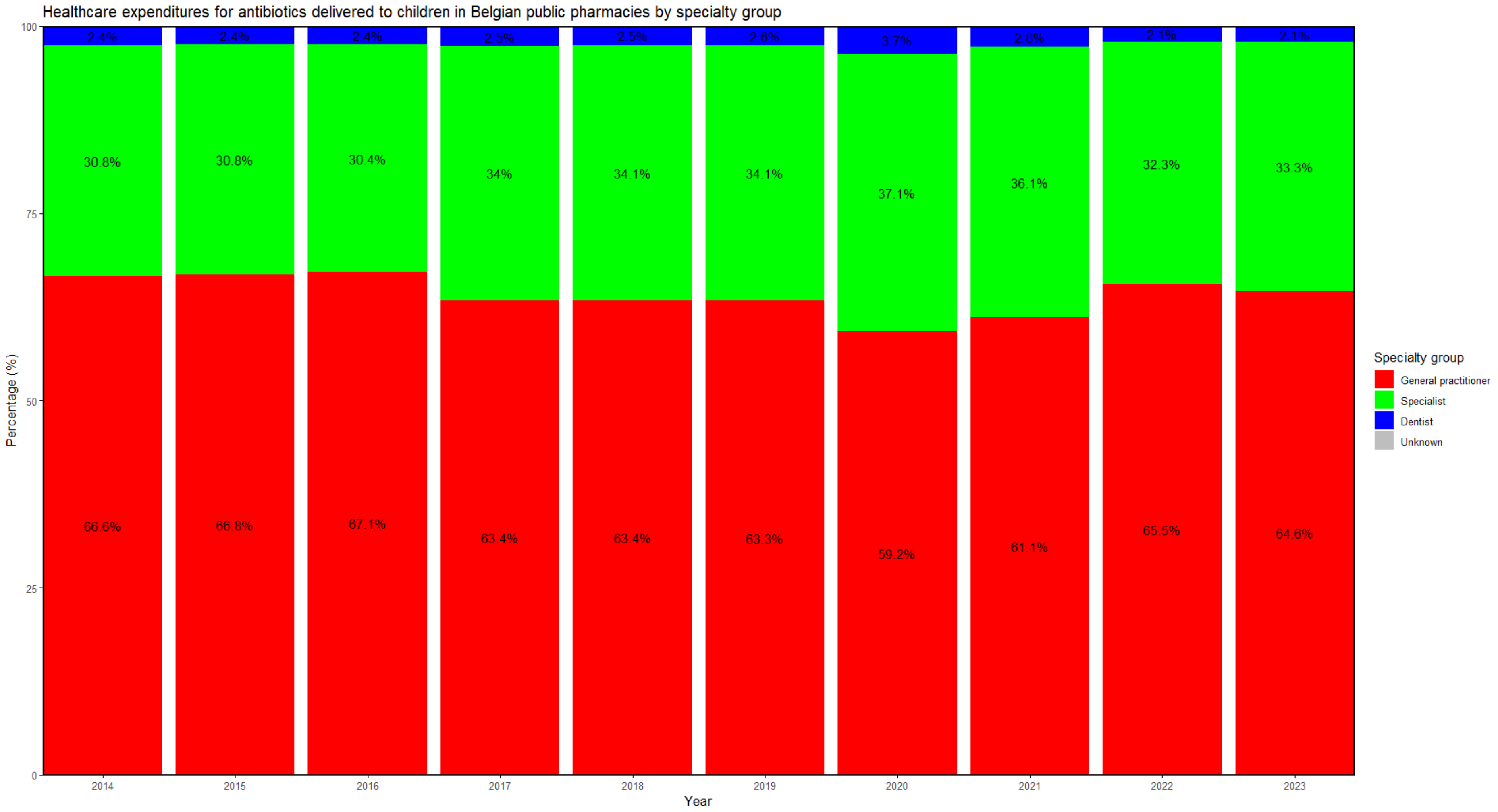

Healthcare expenditures for antibiotics delivered to children in Belgian public pharmacies by specialty group
Percentage (%)
100
75
50
25
0
2.4%
2.4%
2.4%
2.5%
2.5%
2.6%
3.7%
2.8%
2.1%
2.1%
30.8%
30.8%
30.4%
34%
34.1%
34.1%
37.1%
36.1%
32.3%
33.3%
66.6%
66.8%
67.1%
63.4%
63.4%
63.3%
59.2%
61.1%
65.5%
64.6%
2014
2015
2016
2017
2018
2019
2020
2021
2022
2023
Year
Specialty group
General practitioner
Specialist
Dentist
Unknown

(c)

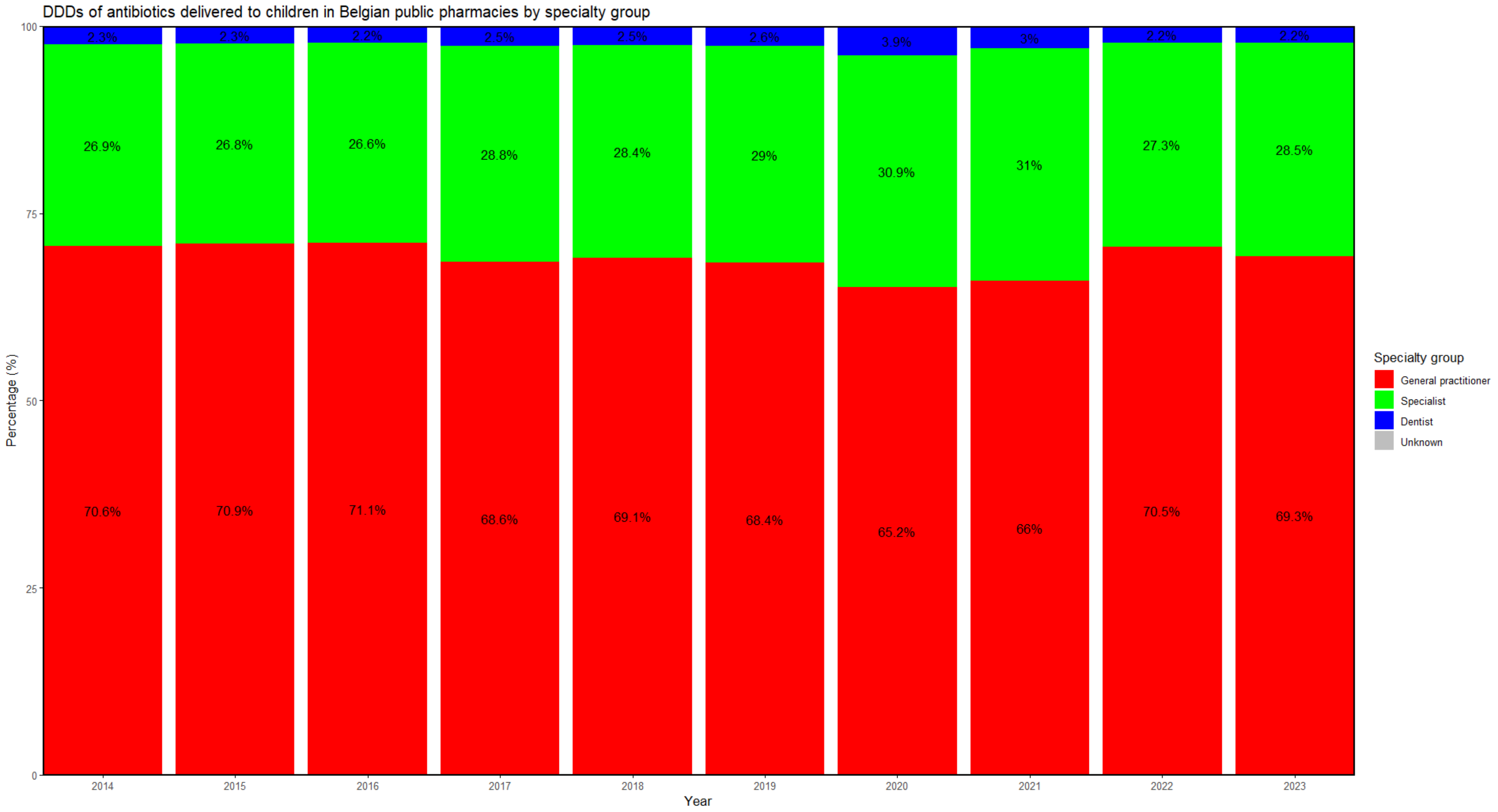

Figure S14. Bar chart of antibiotics delivered to children in Belgian public pharmacies by specialty group, expressed as number of packages (a), healthcare expenditures (b), and Defined Daily Doses (DDDs) (c).
0.043 to 0.046% of the prescriptions has missing data on the specialty of the prescriber (i.e., ‘Unknown’).

(a)

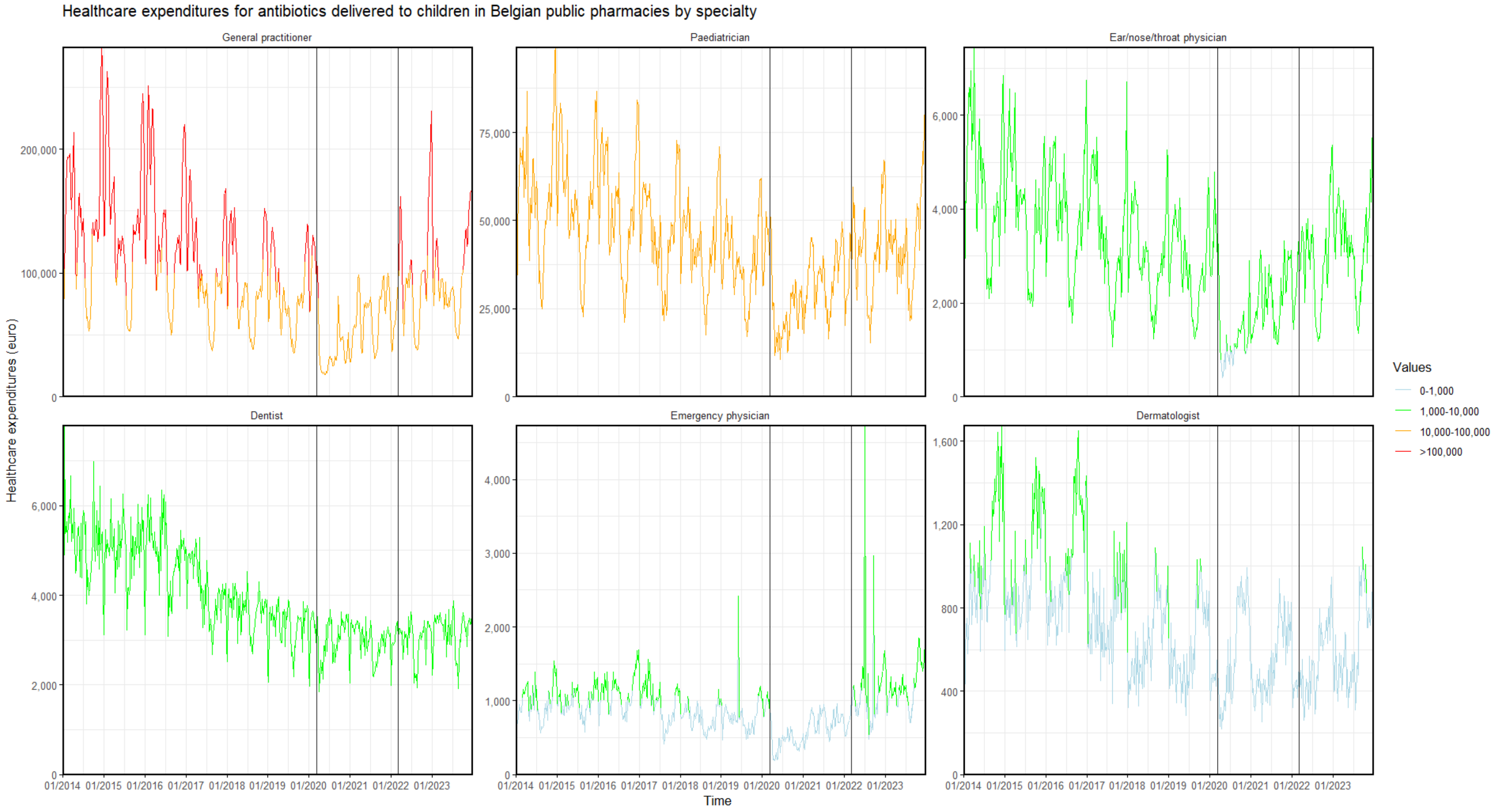

Healthcare expenditures for antibiotics delivered to children in Belgian public pharmacies by specialty
General practitioner
Paediatrician
Ear/nose/throat physician
Dentist
Emergency physician
Dermatologist
Healthcare expenditures (euro)
Time
Values
0-1,000
1,000-10,000
10,000-100,000
>100,000
01/2014 01/2015 01/2016 01/2017 01/2018 01/2019 01/2020 01/2021 01/2022 01/2023

(b)

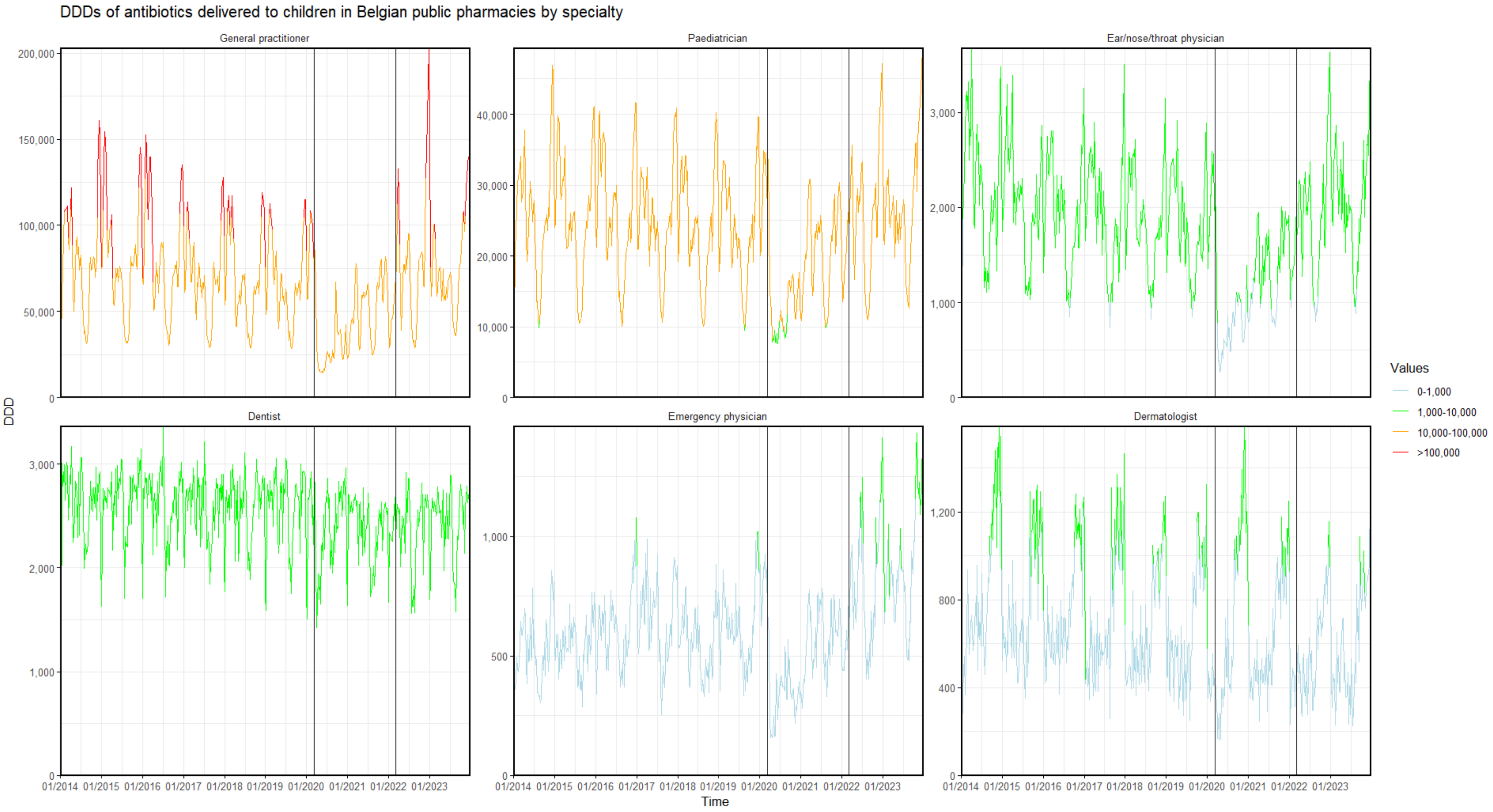

Figure S15. Line chart of antibiotics delivered to children in Belgian public pharmacies by specialty, expressed as healthcare expenditures (a) and Defined Daily Doses (DDDs) (b).

The grey vertical lines represent the start of the COVID-19 pandemic (i.e., the week of 16 March 2020) and the start of the post-COVID period (i.e., the week of 14 March 2022), respectively.

(a)

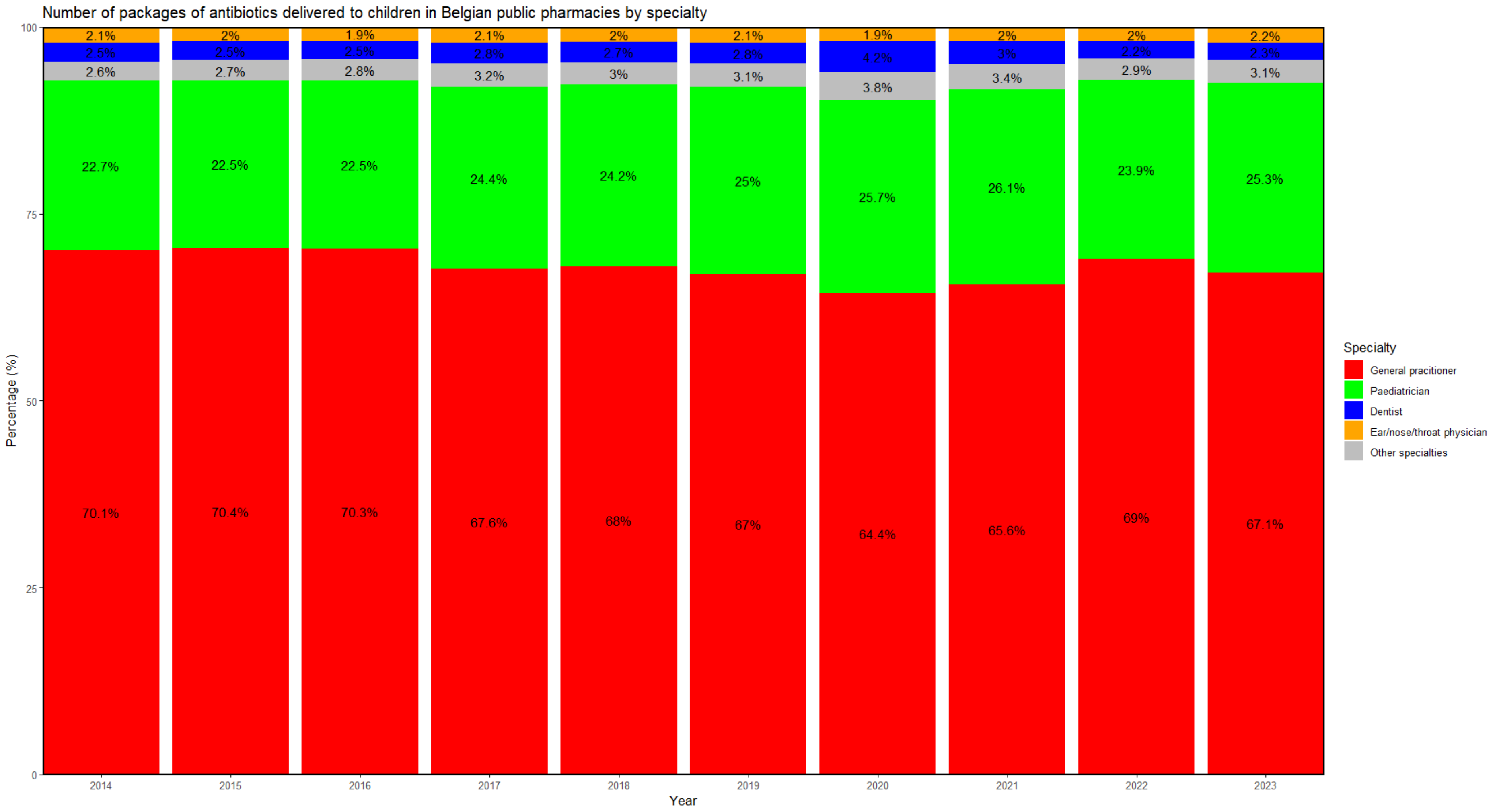
Number of packages of antibiotics delivered to children in Belgian public pharmacies by specialty
Percentage (%)
Year
Specialty
General pracitioner
Paediatrician
Dentist
Ear/nose/throat physician
Other specialties
2014
2015
2016
2017
2018
2019
2020
2021
2022
2023
70.1%
70.4%
70.3%
67.6%
68%
67%
64.4%
65.6%
69%
67.1%
22.7%
22.5%
22.5%
24.4%
24.2%
25%
25.7%
26.1%
23.9%
25.3%
2.6%
2.7%
2.8%
3.2%
3%
3.1%
3.8%
3.4%
2.9%
3.1%
2.5%
2.5%
2.5%
2.8%
2.7%
2.8%
4.2%
3%
2.2%
2.3%
2.1%
2%
1.9%
2.1%
2%
2.1%
1.9%
2%
2%
2.2%

(b)

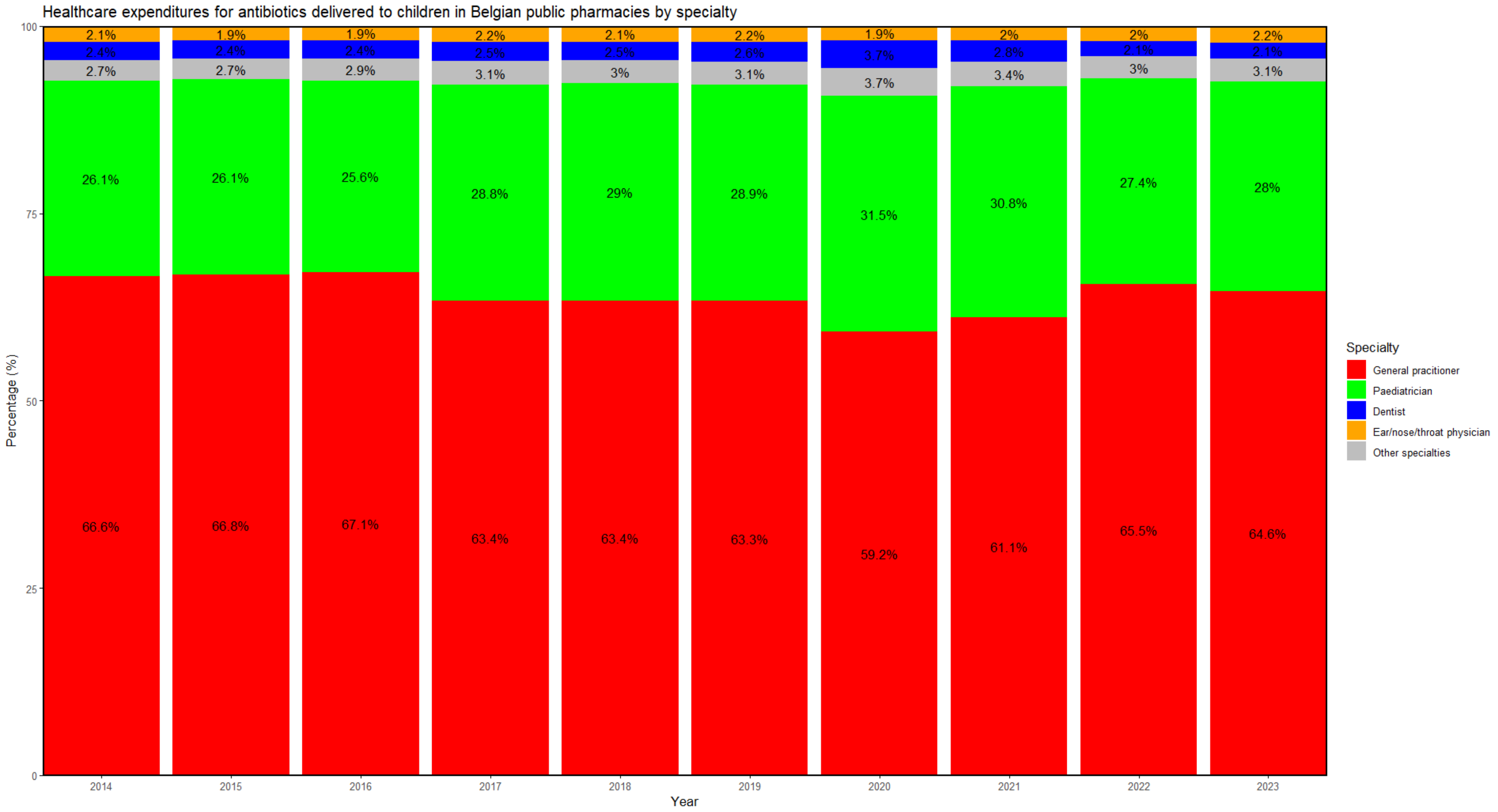
Healthcare expenditures for antibiotics delivered to children in Belgian public pharmacies by specialty
Percentage (%)
Year
Specialty
General pracitioner
Paediatrician
Dentist
Ear/nose/throat physician
Other specialties
2014
2015
2016
2017
2018
2019
2020
2021
2022
2023
2.1%
1.9%
1.9%
2.2%
2.1%
2.2%
1.9%
2%
2%
2.2%
2.4%
2.4%
2.4%
2.5%
2.5%
2.6%
3.7%
2.8%
2.1%
2.1%
2.7%
2.7%
2.9%
3.1%
3%
3.1%
3.7%
3.4%
3%
3.1%
26.1%
26.1%
25.6%
28.8%
29%
28.9%
31.5%
30.8%
27.4%
28%
66.6%
66.8%
67.1%
63.4%
63.4%
63.3%
59.2%
61.1%
65.5%
64.6%

(c)

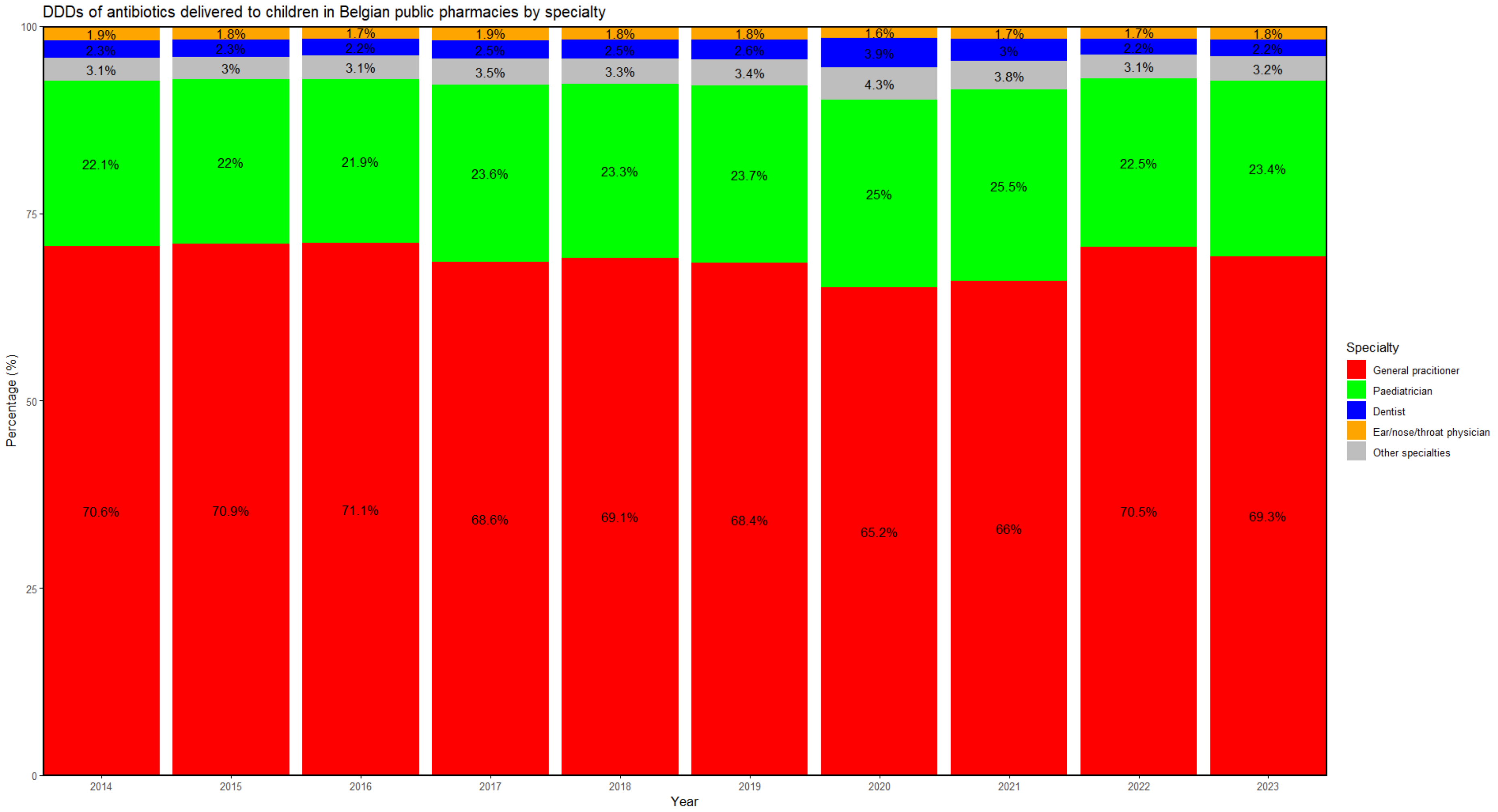

Figure S16. Bar chart of antibiotics delivered to children in Belgian public pharmacies by specialty, expressed as number of packages (a), healthcare expenditures (b), and Defined Daily Doses (DDDs) (c).

GEOGRAPHIC REGIONS

Table S12. Relative change of number of packages, healthcare expenditures and Defined Daily Doses (DDDs) by geographic region, per period (standardised per week).

	Number of packages	Healthcare expenditures	DDDs
During vs before COVID			
Flanders	- 40.9%	- 47.7%	- 38.6%
Walloon region	- 46.0%	- 51.7%	- 41.8%
Brussels Capital Region	- 40.2%	- 47.0%	- 35.8%
Cities	- 44.0%	- 50.9%	- 39.9%
Towns and suburbs	- 44.2%	- 50.3%	- 41.5%
Rural areas	- 46.9%	- 52.2%	- 44.1%
Cities in Flanders	- 40.4%	- 48.4%	- 37.6%
Towns and suburbs in Flanders	- 43.0%	- 49.1%	- 40.7%
Rural areas in Flanders	- 46.1%	- 52.8%	- 44.6%
Cities in Walloon region	- 48.2%	- 54.6%	- 43.4%
Towns and suburbs in Walloon region	- 47.8%	- 53.4%	- 43.8%
Rural areas in Walloon region	- 47.3%	- 51.9%	- 43.9%
After vs during COVID			
Flanders	+ 61.7%	+ 65.3%	+ 68.7%
Walloon region	+ 78.0%	+ 76.9%	+ 83.9%
Brussels Capital Region	+ 58.7%	+ 59.3%	+ 65.7%
Cities	+ 69.1%	+ 72.8%	+ 74.7%
Towns and suburbs	+ 75.0%	+ 75.8%	+ 82.1%
Rural areas	+ 81.6%	+ 82.1%	+ 89.0%
Cities in Flanders	+ 61.5%	+ 67.2%	+ 66.1%
Towns and suburbs in Flanders	+ 70.2%	+ 72.6%	+ 77.2%
Rural areas in Flanders	+ 70.2%	+ 77.1%	+ 79.1%
Cities in Walloon region	+ 79.0%	+ 84.3%	+ 83.9%
Towns and suburbs in Walloon region	+ 90.1%	+ 85.2%	+ 96.2%
Rural areas in Walloon region	+ 88.2%	+ 84.7%	+ 94.1%

(a)

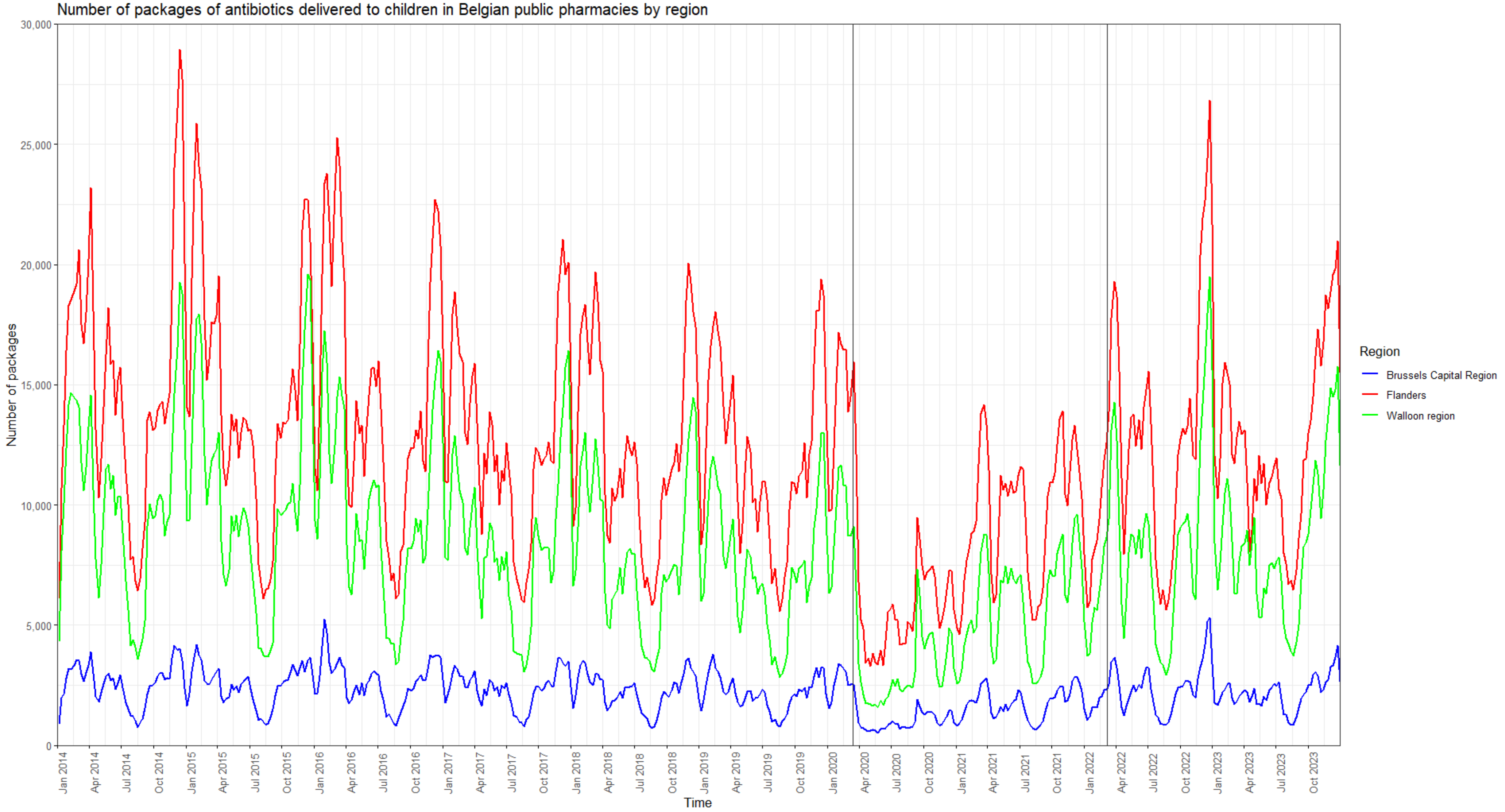

Number of packages of antibiotics delivered to children in Belgian public pharmacies by region
Number of packages
30,000
25,000
20,000
15,000
10,000
5,000
0
Jan 2014
Apr 2014
Jul 2014
Oct 2014
Jan 2015
Apr 2015
Jul 2015
Oct 2015
Jan 2016
Apr 2016
Jul 2016
Oct 2016
Jan 2017
Apr 2017
Jul 2017
Oct 2017
Jan 2018
Apr 2018
Jul 2018
Oct 2018
Jan 2019
Apr 2019
Jul 2019
Oct 2019
Jan 2020
Apr 2020
Jul 2020
Oct 2020
Jan 2021
Apr 2021
Jul 2021
Oct 2021
Jan 2022
Apr 2022
Jul 2022
Oct 2022
Jan 2023
Apr 2023
Jul 2023
Oct 2023
Time
Region
Brussels Capital Region
Flanders
Walloon region

(b)

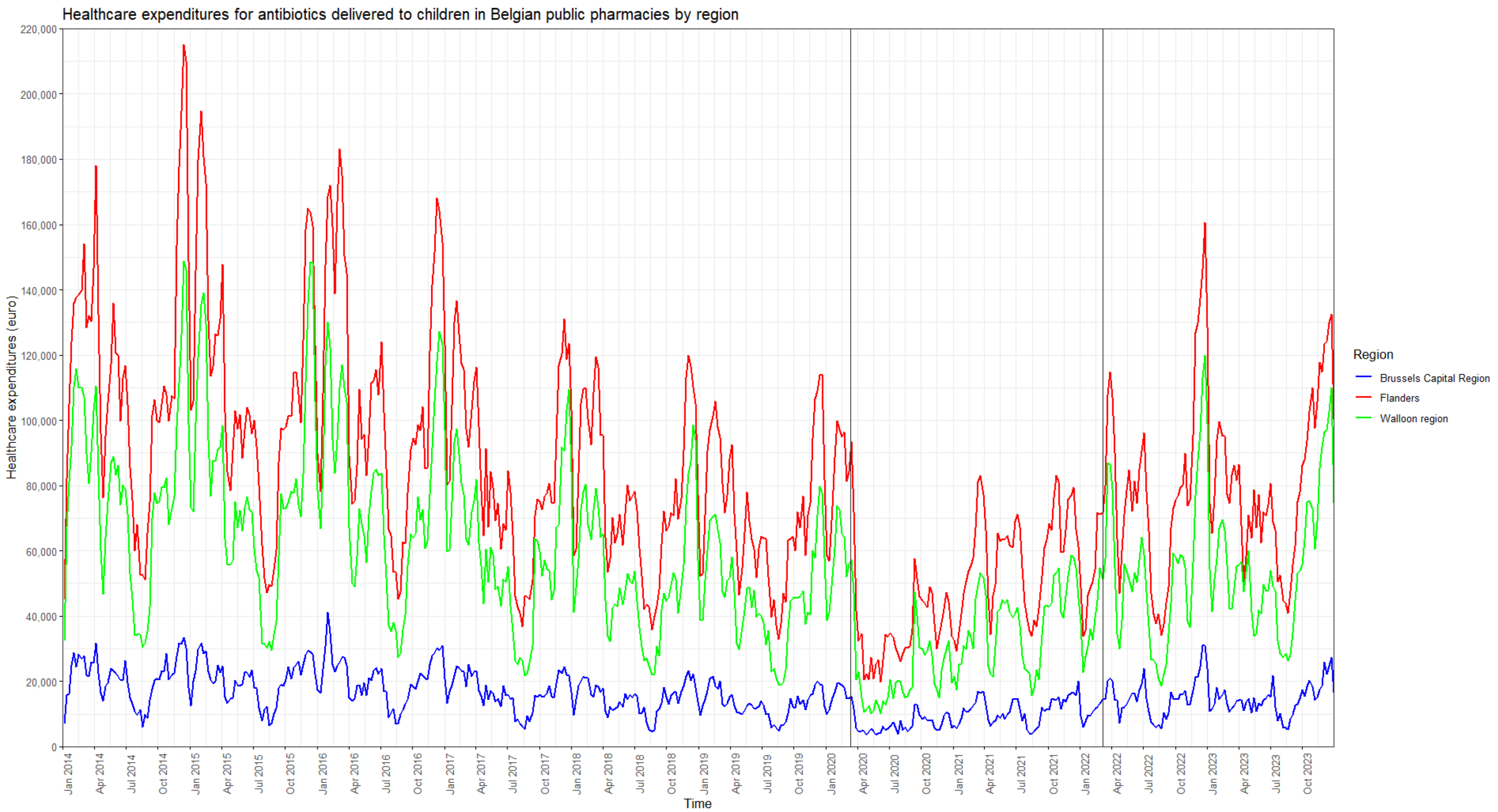

Healthcare expenditures for antibiotics delivered to children in Belgian public pharmacies by region
Healthcare expenditures (euro)
220,000
200,000
180,000
160,000
140,000
120,000
100,000
80,000
60,000
40,000
20,000
0
Jan 2014
Apr 2014
Jul 2014
Oct 2014
Jan 2015
Apr 2015
Jul 2015
Oct 2015
Jan 2016
Apr 2016
Jul 2016
Oct 2016
Jan 2017
Apr 2017
Jul 2017
Oct 2017
Jan 2018
Apr 2018
Jul 2018
Oct 2018
Jan 2019
Apr 2019
Jul 2019
Oct 2019
Jan 2020
Apr 2020
Jul 2020
Oct 2020
Jan 2021
Apr 2021
Jul 2021
Oct 2021
Jan 2022
Apr 2022
Jul 2022
Oct 2022
Jan 2023
Apr 2023
Jul 2023
Oct 2023
Time
Region
Brussels Capital Region
Flanders
Walloon region

(c)

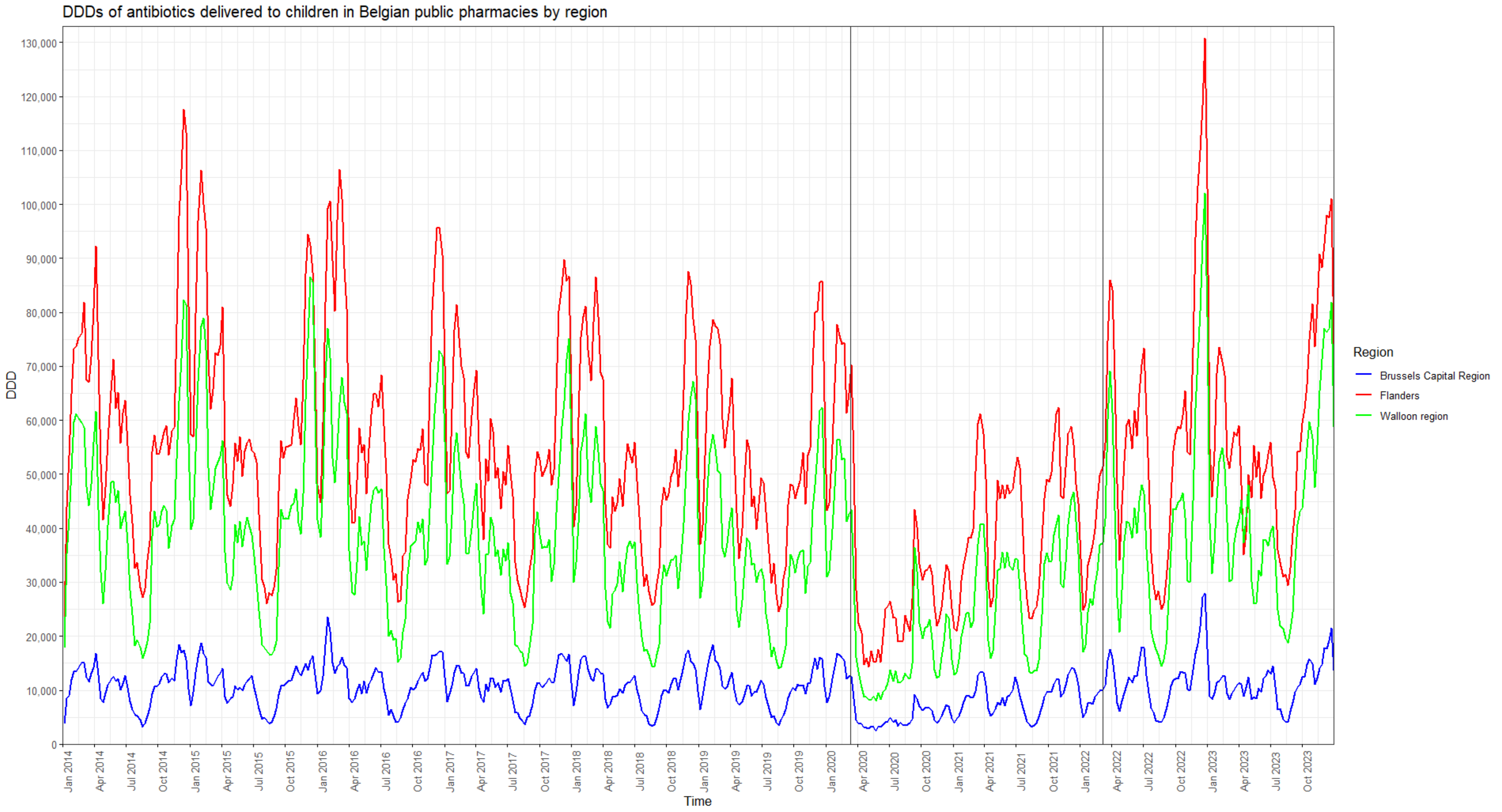

Figure S17. Line chart of antibiotics delivered to children in Belgian public pharmacies by region, expressed as number of packages (a), healthcare expenditures (b), and Defined Daily Doses (DDDs) (c).

The grey vertical lines represent the start of the COVID-19 pandemic (i.e., the week of 16 March 2020) and the start of the post-COVID period (i.e., the week of 14 March 2022), respectively.

(a)

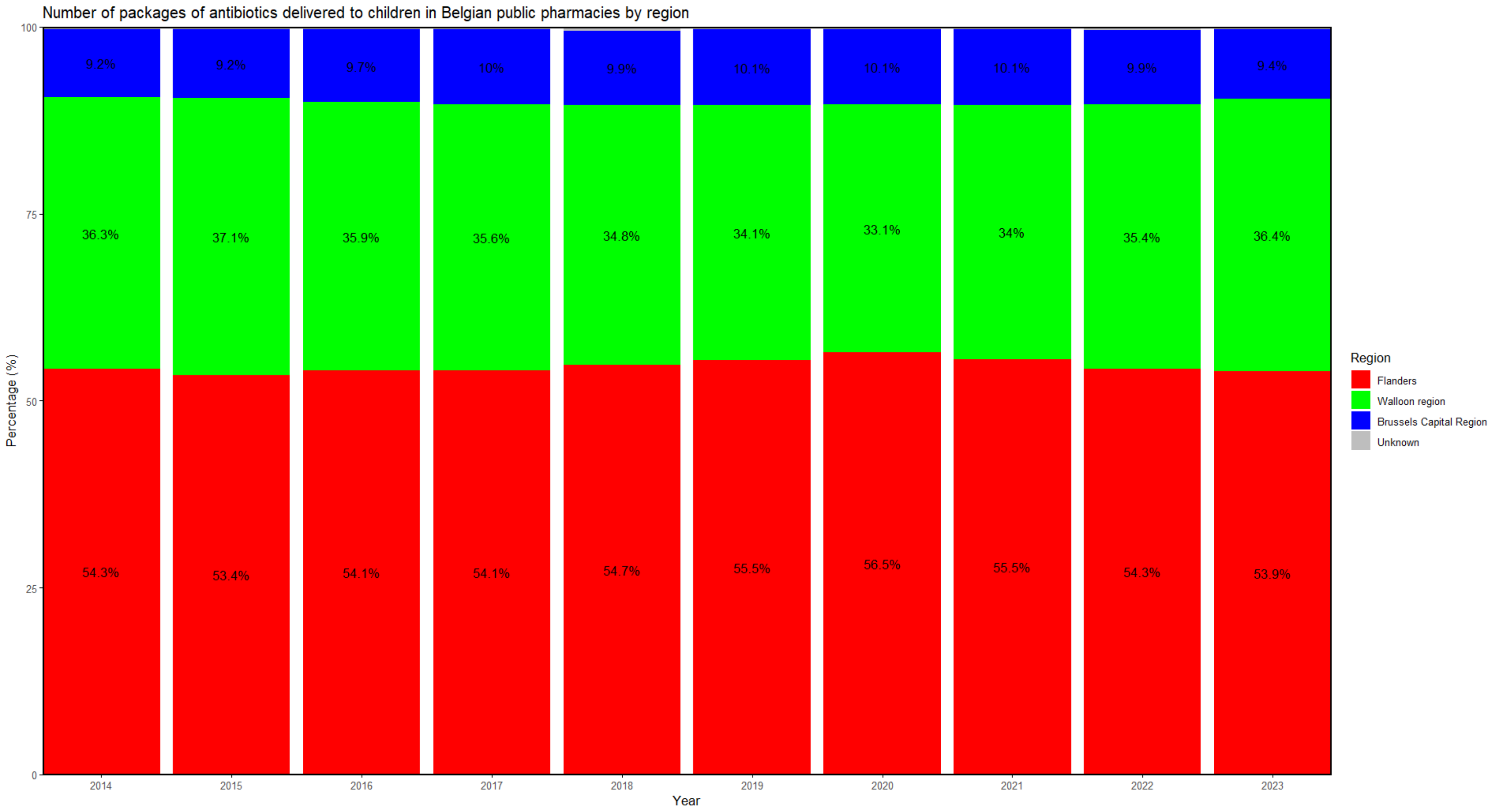

Number of packages of antibiotics delivered to children in Belgian public pharmacies by region
Percentage (%)
Year
2014
2015
2016
2017
2018
2019
2020
2021
2022
2023
0
25
50
75
100
9.2%
9.2%
9.7%
10%
9.9%
10.1%
10.1%
10.1%
9.9%
9.4%
36.3%
37.1%
35.9%
35.6%
34.8%
34.1%
33.1%
34%
35.4%
36.4%
54.3%
53.4%
54.1%
54.1%
54.7%
55.5%
56.5%
55.5%
54.3%
53.9%
Region
Flanders
Walloon region
Brussels Capital Region
Unknown

(b)

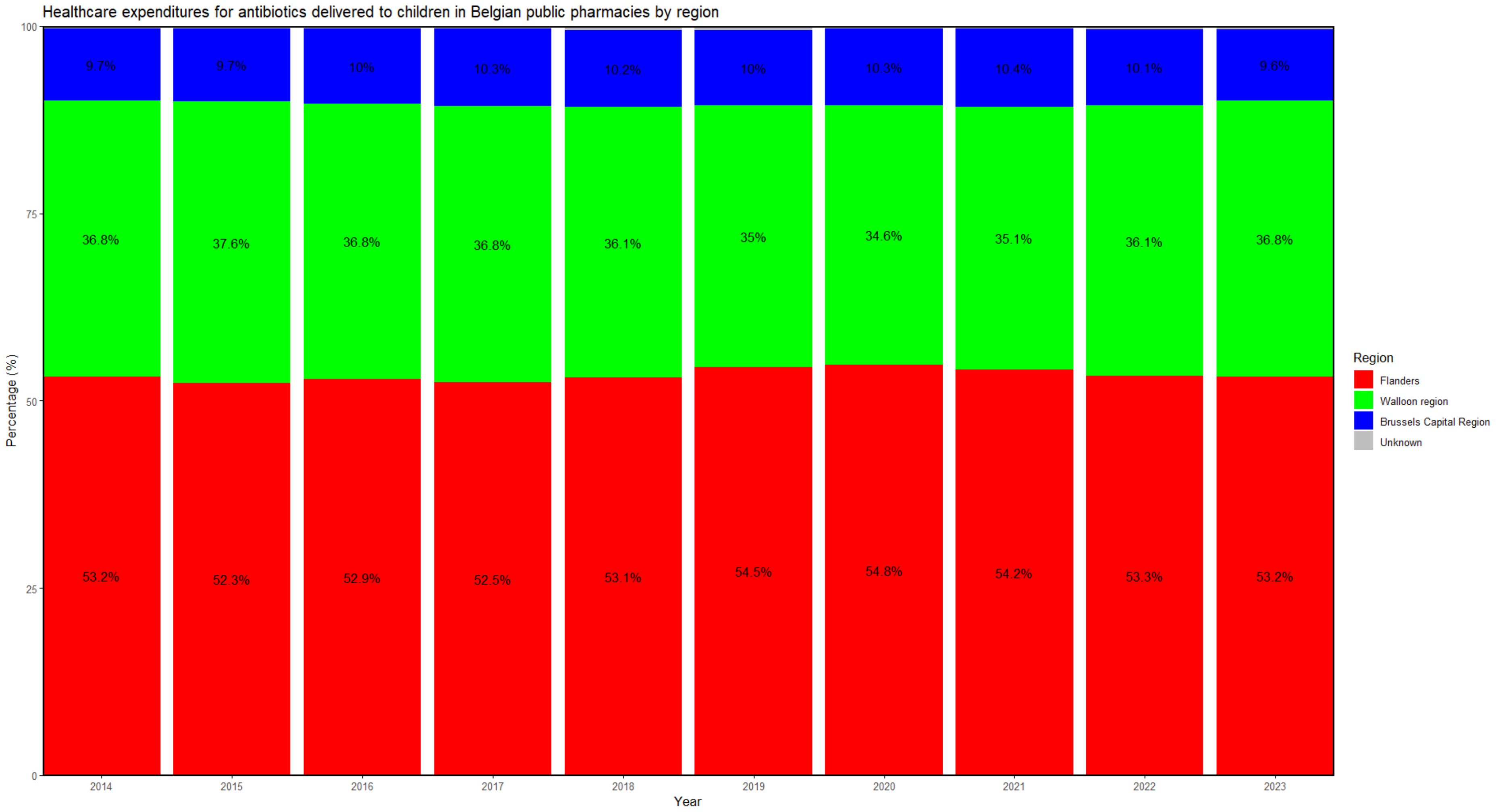
Healthcare expenditures for antibiotics delivered to children in Belgian public pharmacies by region
Percentage (%)
100
75
50
25
0
9.7%
9.7%
10%
10.3%
10.2%
10%
10.3%
10.4%
10.1%
9.6%
36.8%
37.6%
36.8%
36.8%
36.1%
35%
34.6%
35.1%
36.1%
36.8%
53.2%
52.3%
52.9%
52.5%
53.1%
54.5%
54.8%
54.2%
53.3%
53.2%
2014
2015
2016
2017
2018
2019
2020
2021
2022
2023
Year
Region
Flanders
Walloon region
Brussels Capital Region
Unknown

(c)

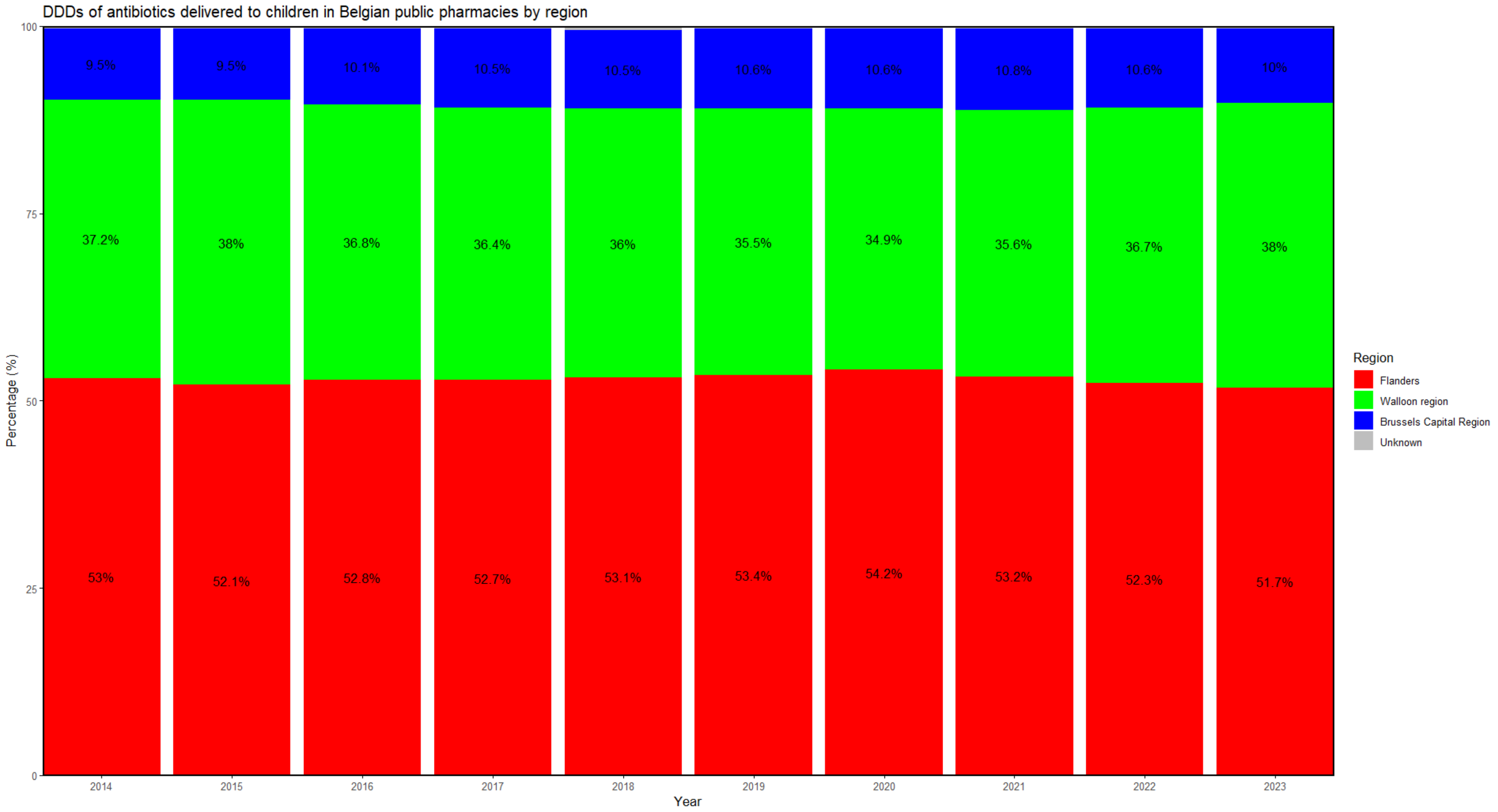

Figure S18. Bar chart of antibiotics delivered to children in Belgian public pharmacies by region, expressed as number of packages (a), healthcare expenditures (b), and Defined Daily Doses (DDDs) (c).

0.34 to 0.37% of the prescriptions has missing data on the geographical region of the child (i.e., ‘Unknown’).

(a)

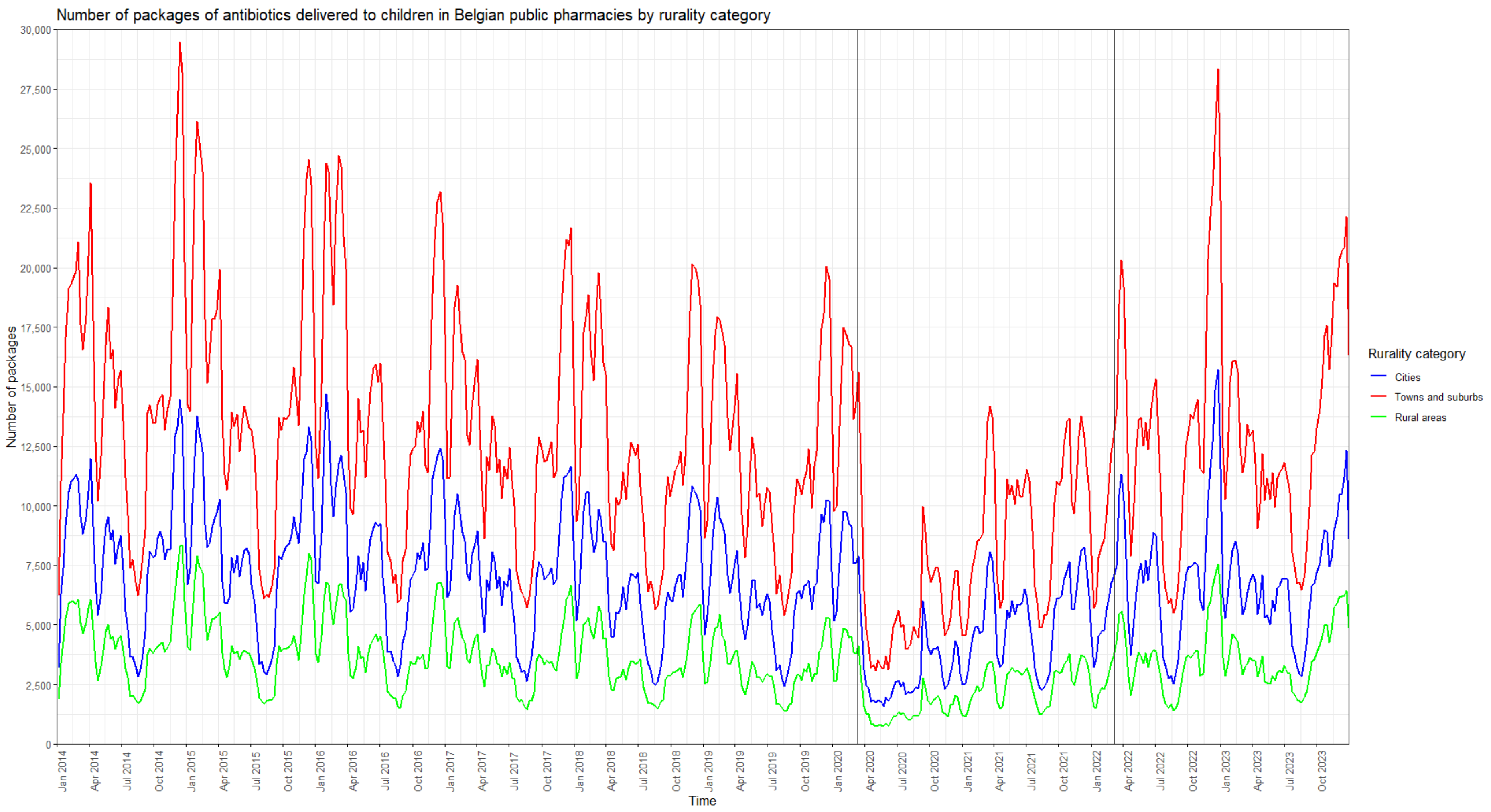

Number of packages of antibiotics delivered to children in Belgian public pharmacies by rurality category
Number of packages
30,000
27,500
25,000
22,500
20,000
17,500
15,000
12,500
10,000
7,500
5,000
2,500
0
Jan 2014
Apr 2014
Jul 2014
Oct 2014
Jan 2015
Apr 2015
Jul 2015
Oct 2015
Jan 2016
Apr 2016
Jul 2016
Oct 2016
Jan 2017
Apr 2017
Jul 2017
Oct 2017
Jan 2018
Apr 2018
Jul 2018
Oct 2018
Jan 2019
Apr 2019
Jul 2019
Oct 2019
Jan 2020
Apr 2020
Jul 2020
Oct 2020
Jan 2021
Apr 2021
Jul 2021
Oct 2021
Jan 2022
Apr 2022
Jul 2022
Oct 2022
Jan 2023
Apr 2023
Jul 2023
Oct 2023
Time
Rurality category
Cities
Towns and suburbs
Rural areas

(b)

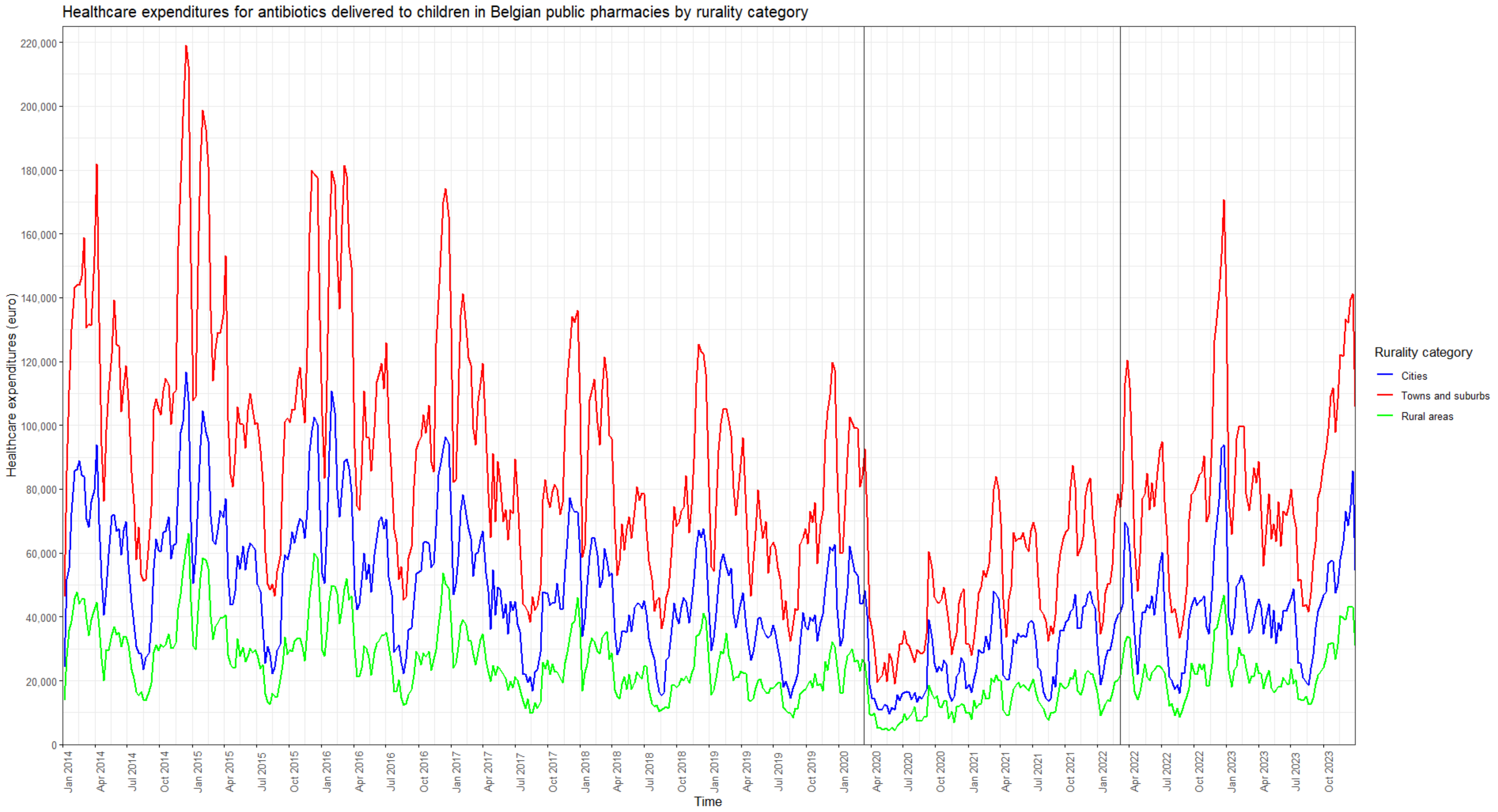
Healthcare expenditures for antibiotics delivered to children in Belgian public pharmacies by rurality category
Healthcare expenditures (euro)
220,000
200,000
180,000
160,000
140,000
120,000
100,000
80,000
60,000
40,000
20,000
0
Jan 2014
Apr 2014
Jul 2014
Oct 2014
Jan 2015
Apr 2015
Jul 2015
Oct 2015
Jan 2016
Apr 2016
Jul 2016
Oct 2016
Jan 2017
Apr 2017
Jul 2017
Oct 2017
Jan 2018
Apr 2018
Jul 2018
Oct 2018
Jan 2019
Apr 2019
Jul 2019
Oct 2019
Jan 2020
Apr 2020
Jul 2020
Oct 2020
Jan 2021
Apr 2021
Jul 2021
Oct 2021
Jan 2022
Apr 2022
Jul 2022
Oct 2022
Jan 2023
Apr 2023
Jul 2023
Oct 2023
Time
Rurality category
Cities
Towns and suburbs
Rural areas

(c)

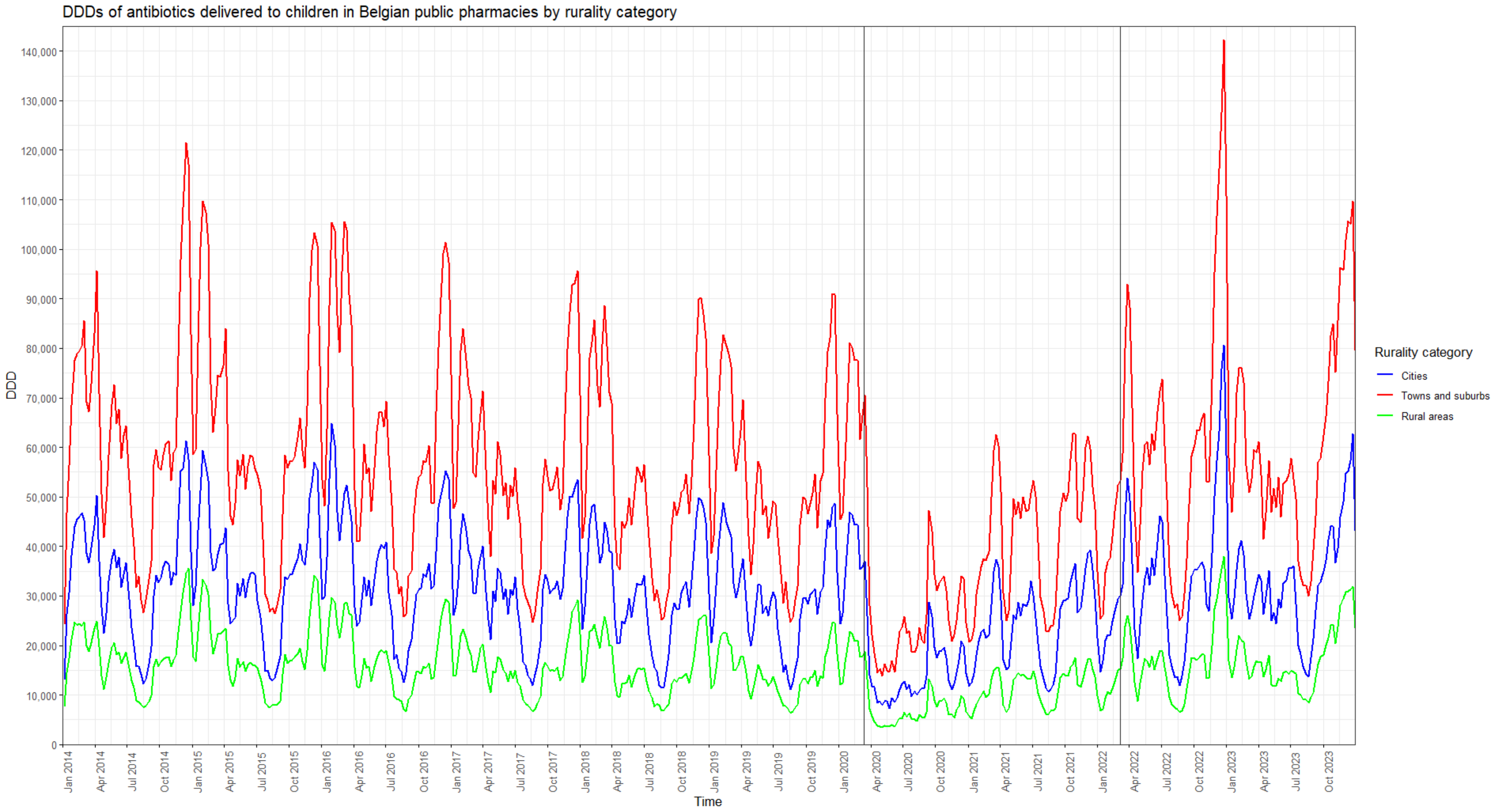

Figure S19. Line chart of antibiotics delivered to children in Belgian public pharmacies by rurality category, expressed as number of packages (a), healthcare expenditures (b), and Defined Daily Doses (DDDs) (c).

The grey vertical lines represent the start of the COVID-19 pandemic (i.e., the week of 16 March 2020) and the start of the post-COVID period (i.e., the week of 14 March 2022), respectively.

(a)

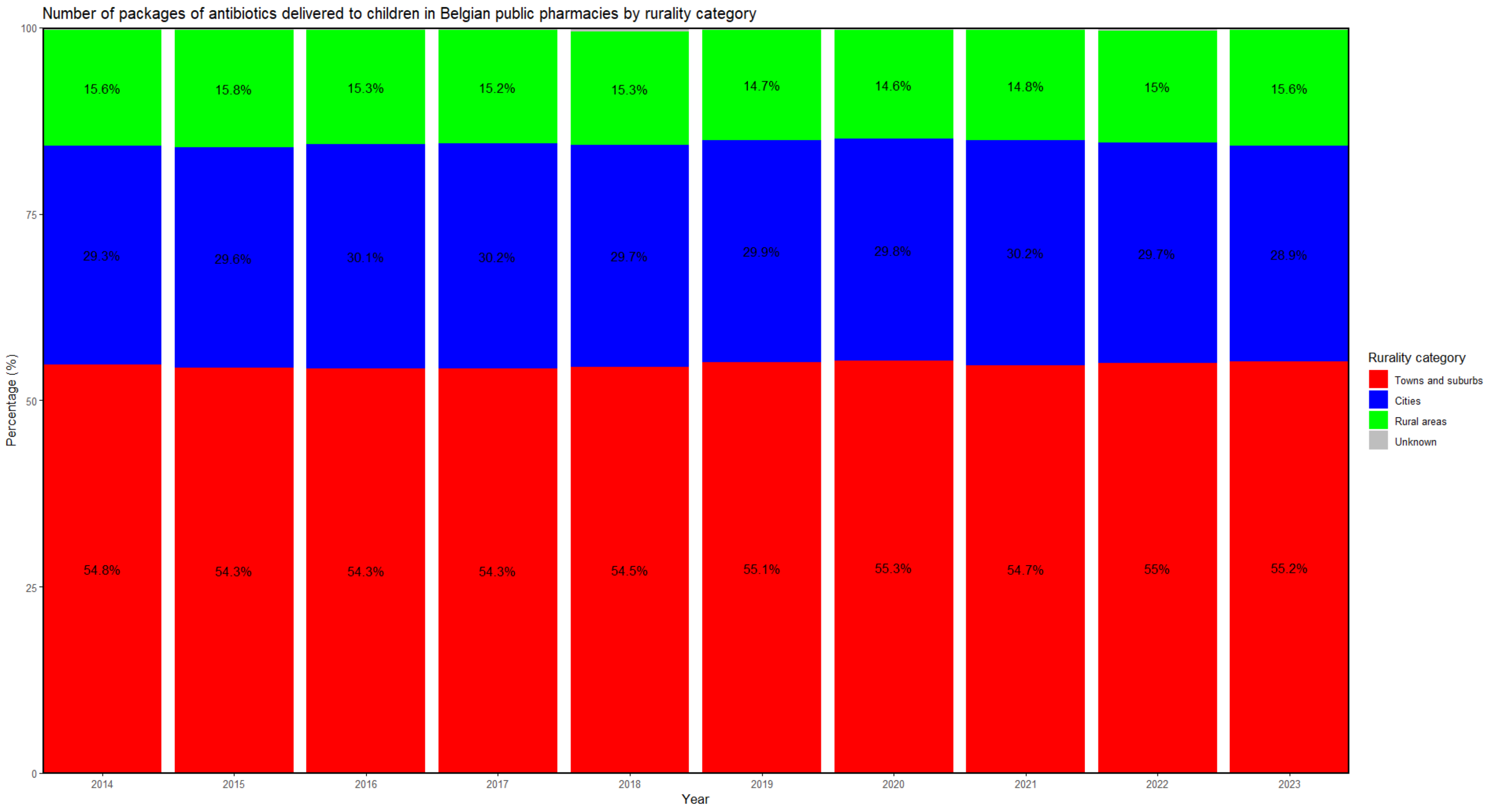
Number of packages of antibiotics delivered to children in Belgian public pharmacies by rurality category
Percentage (%)
100
75
50
25
0
Year
2014
2015
2016
2017
2018
2019
2020
2021
2022
2023
15.6%
15.8%
15.3%
15.2%
15.3%
14.7%
14.6%
14.8%
15%
15.6%
29.3%
29.6%
30.1%
30.2%
29.7%
29.9%
29.8%
30.2%
29.7%
28.9%
54.8%
54.3%
54.3%
54.3%
54.5%
55.1%
55.3%
54.7%
55%
55.2%
Rurality category
Towns and suburbs
Cities
Rural areas
Unknown

(b)

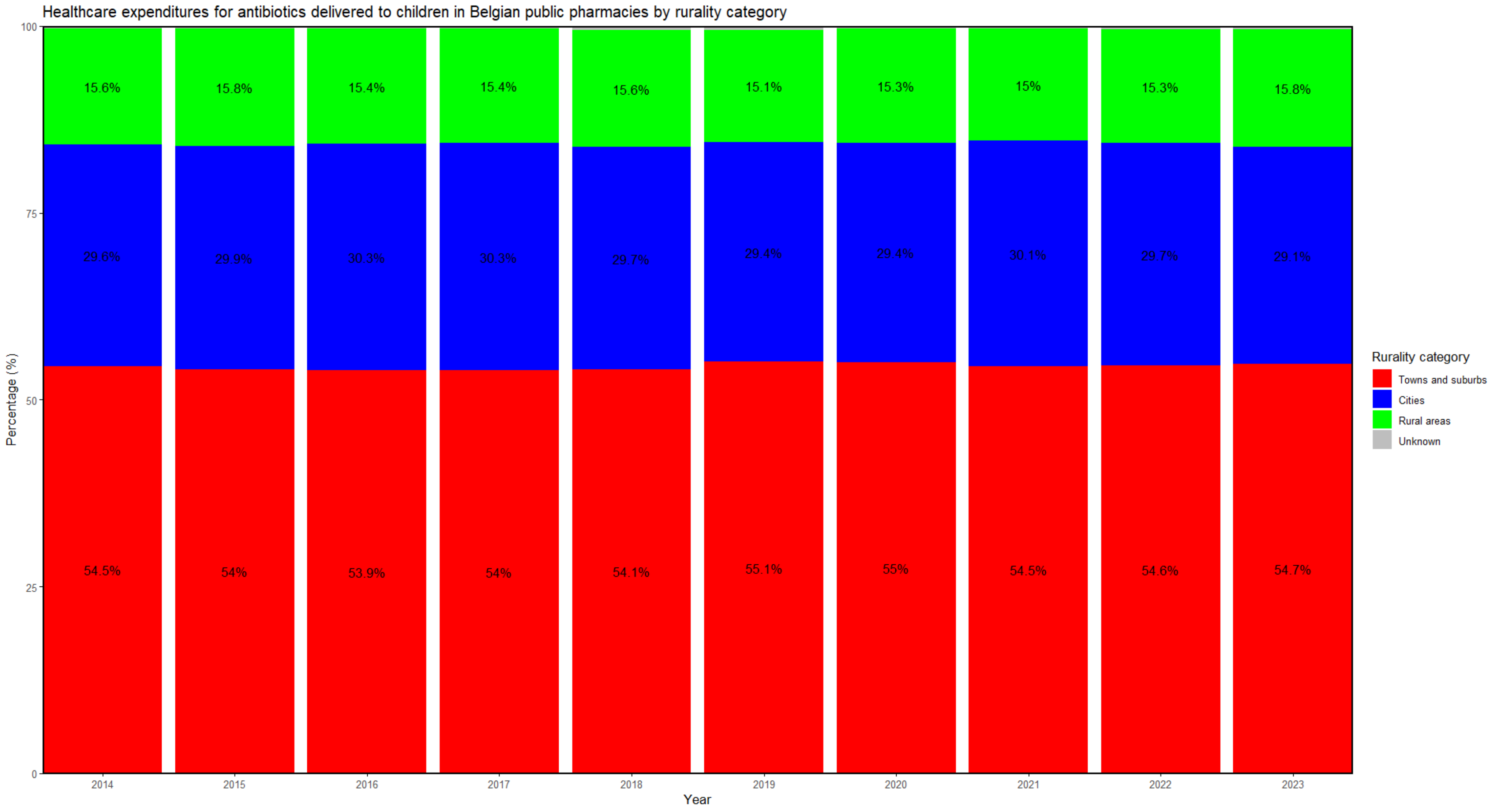

Healthcare expenditures for antibiotics delivered to children in Belgian public pharmacies by rurality category
Percentage (%)
Year
2014
2015
2016
2017
2018
2019
2020
2021
2022
2023
0
25
50
75
100
15.6%
15.8%
15.4%
15.4%
15.6%
15.1%
15.3%
15%
15.3%
15.8%
29.6%
29.9%
30.3%
30.3%
29.7%
29.4%
29.4%
30.1%
29.7%
29.1%
54.5%
54%
53.9%
54%
54.1%
55.1%
55%
54.5%
54.6%
54.7%
Rurality category
Towns and suburbs
Cities
Rural areas
Unknown

(c)

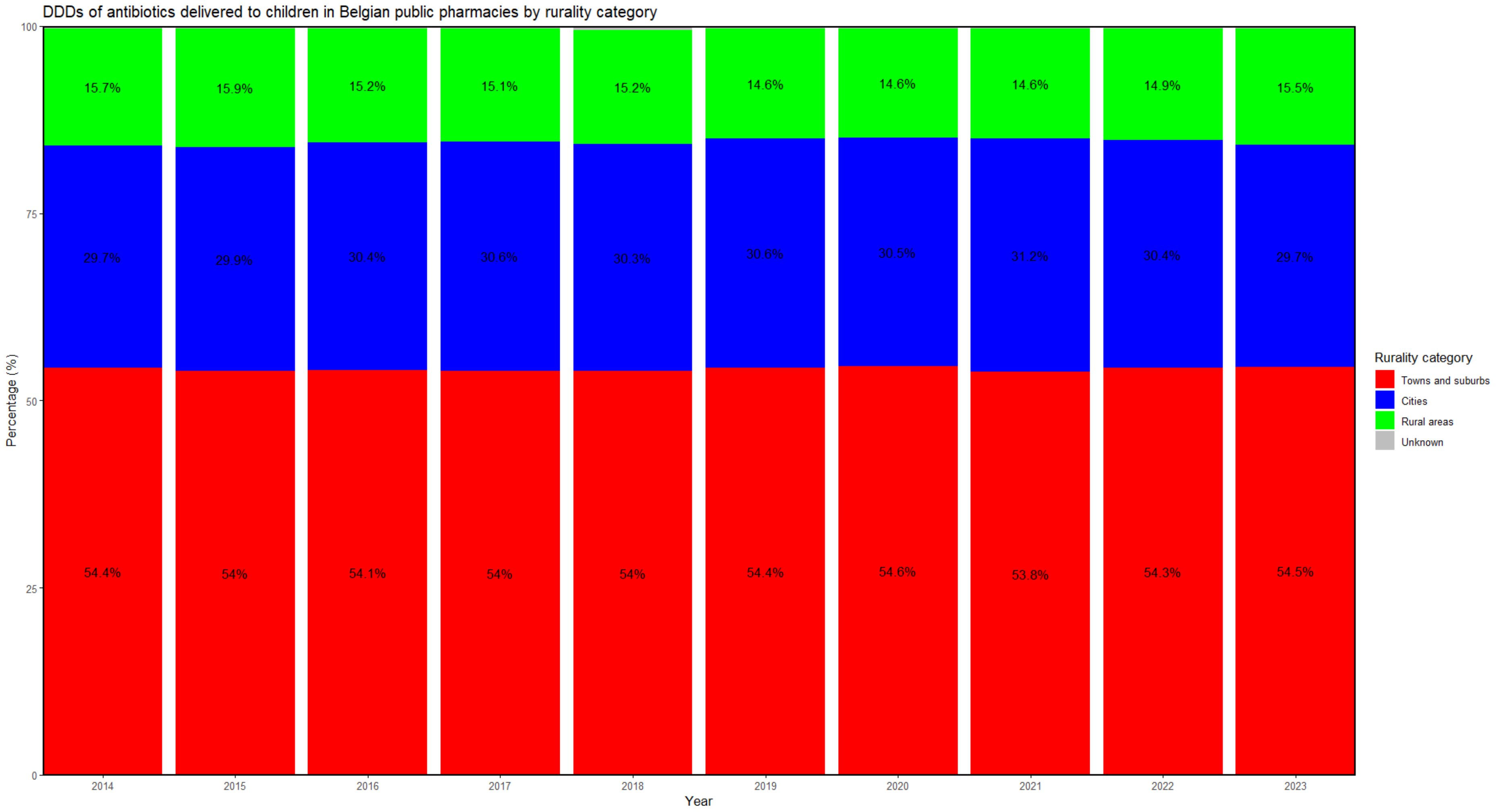

Figure S20. Bar chart of antibiotics delivered to children in Belgian public pharmacies by rurality category, expressed as number of packages (a), healthcare expenditures (b), and Defined Daily Doses (DDDs) (c).
0.34 to 0.37% of the prescriptions has missing data on the geographical region of the child (i.e., ‘Unknown’).

(a)

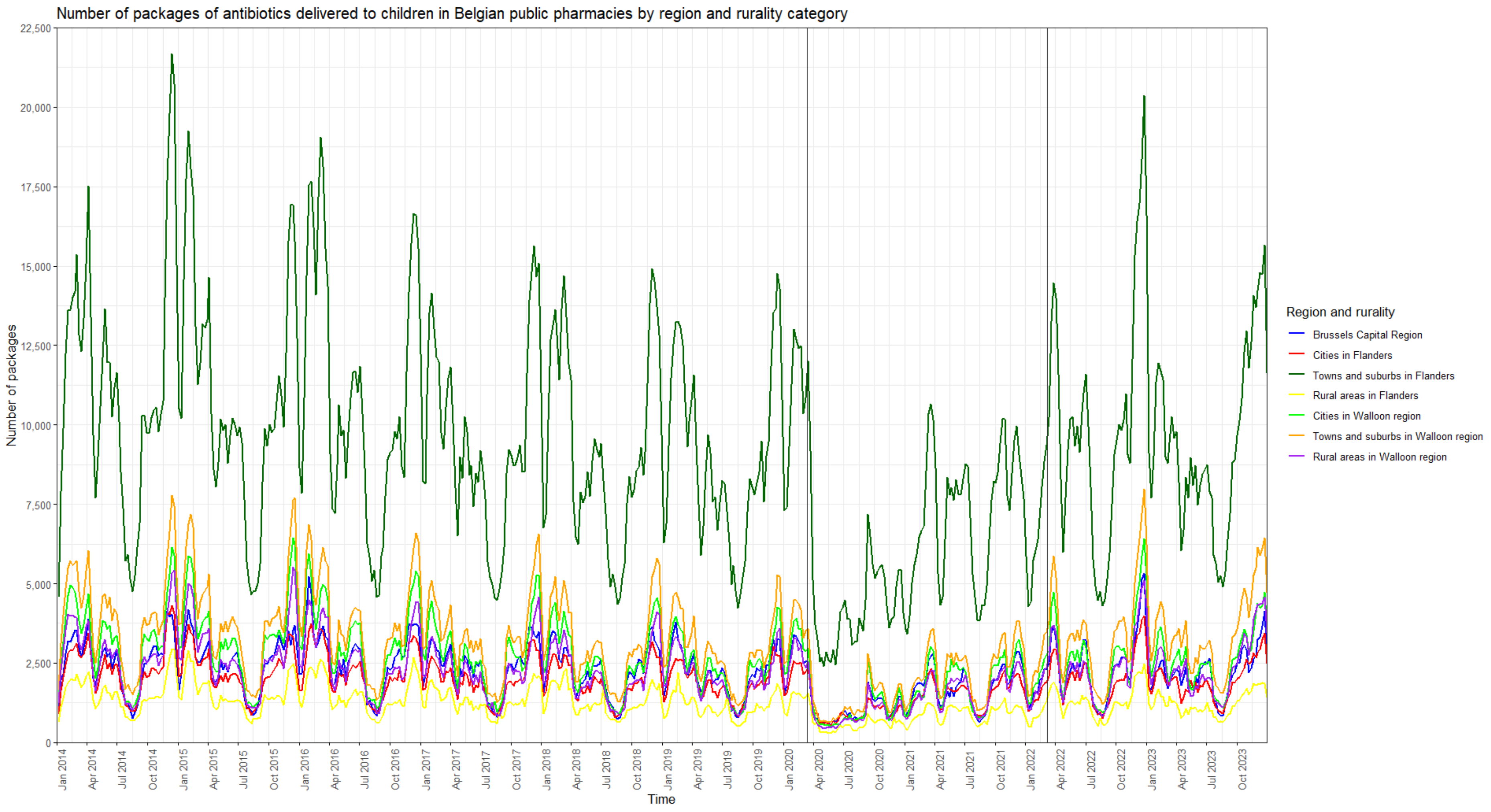
Number of packages of antibiotics delivered to children in Belgian public pharmacies by region and rurality category
Number of packages
Time
Region and rurality
Brussels Capital Region
Cities in Flanders
Towns and suburbs in Flanders
Rural areas in Flanders
Cities in Walloon region
Towns and suburbs in Walloon region
Rural areas in Walloon region

(b)

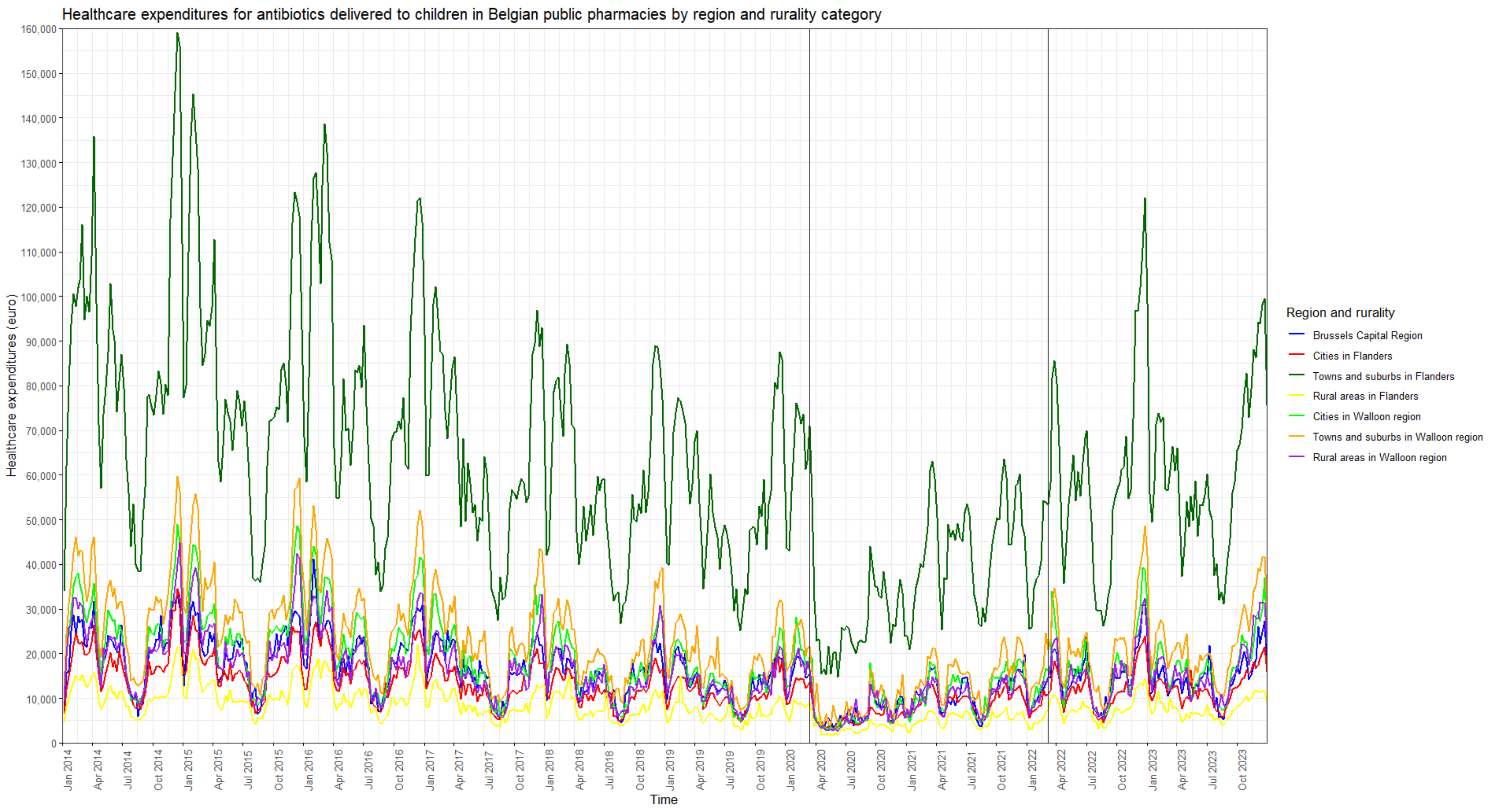

Healthcare expenditures for antibiotics delivered to children in Belgian public pharmacies by region and rurality category
Healthcare expenditures (euro)
Time
Region and rurality
Brussels Capital Region
Cities in Flanders
Towns and suburbs in Flanders
Rural areas in Flanders
Cities in Walloon region
Towns and suburbs in Walloon region
Rural areas in Walloon region

(c)

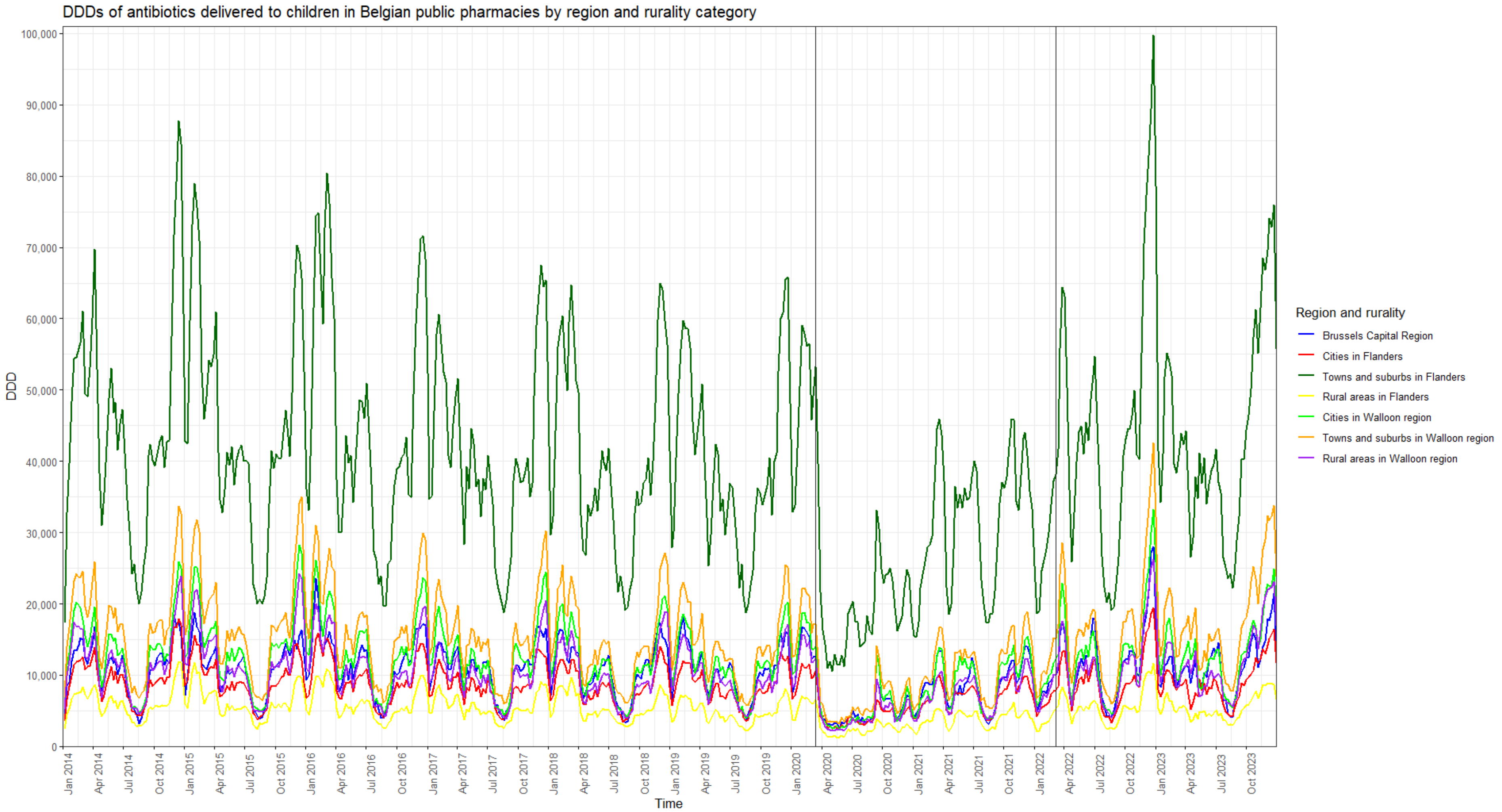

Figure S21. Line chart of antibiotics delivered to children in Belgian public pharmacies by region and rurality category, expressed as number of packages (a), healthcare expenditures (b), and Defined Daily Doses (DDDs) (c).

The grey vertical lines represent the start of the COVID-19 pandemic (i.e., the week of 16 March 2020) and the start of the post-COVID period (i.e., the week of 14 March 2022), respectively.

ANTIBIOTIC CLASSES AND COMPOUNDS

Table S13. Proportion of Amoxicillin packages compared to the sum of Amoxicillin and Amoxicillin / clavulanate packages, per year.

Year	Packages of Amoxicillin divided by (Amoxicillin + Amoxicillin / clavulanate)
2014	69.04%
2015	70.52%
2016	71.95%
2017	73.52%
2018	74.52%
2019	75.92%
2020	74.86%
2021	76.64%
2022	77.57%
2023	78.72%

Table S14. Relative change of number of packages, healthcare expenditures and Defined Daily Doses (DDDs) by ATC-3 class, per period (standardised per week).

	Number of packages	Healthcare expenditures	DDDs
During vs before COVID			
Penicillins (J01C)	- 41.6%	- 49.6%	- 38.2%
Macrolides, lincosamides and streptogramins (J01F)	- 44.0%	- 54.3%	- 50.4%
Other beta-lactam antibacterials (J01D)	- 54.8%	- 59.5%	- 35.9%
Other antibacterials (J01X)	- 15.1%	- 7.03%	- 17.3%
Amphenicols (J01B)	- 53.6%	- 47.2%	- 55.9%
Tetracyclines (J01A)	+ 0.65%	- 21.7%	+ 0.99%
Sulfonamides and trimethoprim (J01E)	- 77.3%	- 78.3%	- 63.3%
Aminoglycoside antibacterials (J01G)	- 59.6%	- 34.9%	- 4.94%
Quinolone antibacterials (J01M)	- 64.3%	- 73.5%	- 59.1%
After vs during COVID			
Penicillins (J01C)	+ 68.0%	+ 77.0%	+ 81.7%
Macrolides, lincosamides and streptogramins (J01F)	+ 90.7%	+ 100.1%	+ 93.0%
Other beta-lactam antibacterials (J01D)	- 15.6%	- 10.7%	- 13.7%
Other antibacterials (J01X)	- 20.0%	- 9.80%	- 11.3%
Amphenicols (J01B)	+ 53.1%	+ 62.6%	+ 53.1%
Tetracyclines (J01A)	+ 20.9%	+ 14.4%	+ 12.8%
Sulfonamides and trimethoprim (J01E)	+ 15.5%	+ 22.6%	+ 15.6%
Aminoglycoside antibacterials (J01G)	- 17.7%	- 11.3%	- 20.8%
Quinolone antibacterials (J01M)	- 7.78%	+ 3.52%	- 1.32%

ATC: Anatomical Therapeutic Chemical classification system

Table S15. Relative change of number of packages, healthcare expenditures and Defined Daily Doses (DDDs) by compound, per period (standardised per week).

	Main indication in children	Number of packages	Healthcare expenditures	DDDs
During vs before COVID				
Amoxicillin	RTI	- 40.1%	- 48.0%	- 36.1%
Amoxicillin / clavulanate	RTI	- 49.9%	- 57.7%	- 48.5%
Azithromycin	RTI	- 26.4%	- 36.9%	- 28.1%

Clarithromycin	RTI	- 72.0%	- 77.6%	- 70.3%
Flucloxacillin	SSTI	+ 6.79%	- 7.58%	+ 6.02%
Sulfamethoxazole and trimethoprim	RTI	- 77.3%	- 78.3%	- 63.3%
Cefuroxime	UTI	- 16.6%	- 31.5%	- 17.2%
Doxycycline	SSTI	+ 7.76%	- 3.28%	+ 10.8%
Nitrofurantoin	UTI	- 15.6%	- 24.1%	- 16.0%
Fosfomycin	UTI	- 24.8%	- 34.5%	- 24.8%
Ciprofloxacin	UTI	- 62.2%	- 72.0%	- 56.2%
Phenoxymethylpenicillin	RTI	- 100.0%	- 100.0%	- 100.0%
After vs during COVID				
Amoxicillin	RTI	+ 72.9%	+ 82.6%	+ 88.0%
Amoxicillin / clavulanate	RTI	+ 52.9%	+ 60.2%	+ 54.9%
Azithromycin	RTI	+ 96.2%	+ 106.2%	+ 96.9%
Clarithromycin	RTI	+ 82.1%	+ 92.3%	+ 92.7%
Flucloxacillin	SSTI	+ 62.9%	+ 71.5%	+ 65.2%
Sulfamethoxazole and trimethoprim	RTI	+ 15.5%	+ 22.6%	+ 15.6%
Cefuroxime	UTI	- 21.0%	- 17.0%	- 14.2%
Doxycycline	SSTI	+ 67.8%	+ 76.1%	+ 65.4%
Nitrofurantoin	UTI	- 15.7%	- 11.2%	- 11.8%
Fosfomycin	UTI	- 4.56%	+ 0.09%	- 4.56%
Ciprofloxacin	UTI	- 8.78%	+ 0.44%	- 4.57%
Phenoxymethylpenicillin	RTI	NA	NA	NA

RTI: respiratory tract infection; SSTI: skin and soft tissue infection; UTI: urinary tract infection; NA: not applicable.

Table S16. Relative change of number of packages, healthcare expenditures and Defined Daily Doses (DDDs) by spectrum of antibiotic activity, per period (standardised per week).

	Number of packages	Healthcare expenditures	DDDs
During vs before COVID			
Broad-spectrum	- 42.3%	- 50.8%	- 39.4%
Narrow-spectrum	+ 1.09%	- 12.4%	- 4.04%
After vs during COVID			
Broad-spectrum	+ 68.9%	+ 77.5%	+ 76.0%
Narrow-spectrum	+ 62.9%	+ 71.5%	+ 65.2%

Broad-spectrum antibiotics: Amoxicillin, Amoxicillin / clavulanate, Azithromycin, Clarithromycin, Sulfamethoxazole and trimethoprim, Cefuroxime, Doxycycline, Nitrofurantoin, Fosfomycin, and Ciprofloxacin; Narrow-spectrum antibiotics: Flucloxacillin and Phenoxymethylpenicillin.

(a)

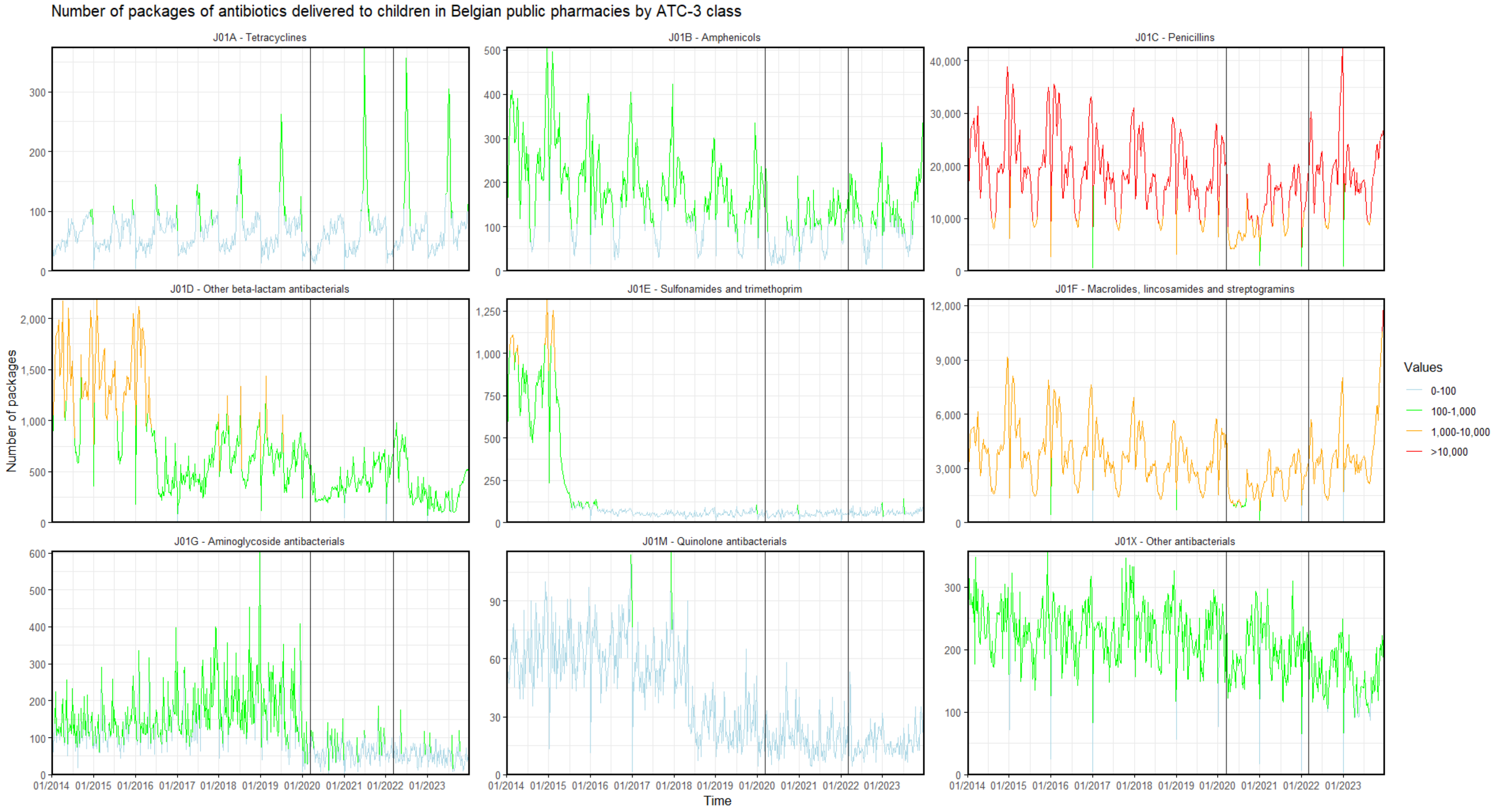

Number of packages of antibiotics delivered to children in Belgian public pharmacies by ATC-3 class
J01A - Tetracyclines
J01B - Amphenicols
J01C - Penicillins
J01D - Other beta-lactam antibacterials
J01E - Sulfonamides and trimethoprim
J01F - Macrolides, lincosamides and streptogramins
J01G - Aminoglycoside antibacterials
J01M - Quinolone antibacterials
J01X - Other antibacterials
Number of packages
Time
01/2014 01/2015 01/2016 01/2017 01/2018 01/2019 01/2020 01/2021 01/2022 01/2023
Values
0-100
100-1,000
1,000-10,000
>10,000

(b)

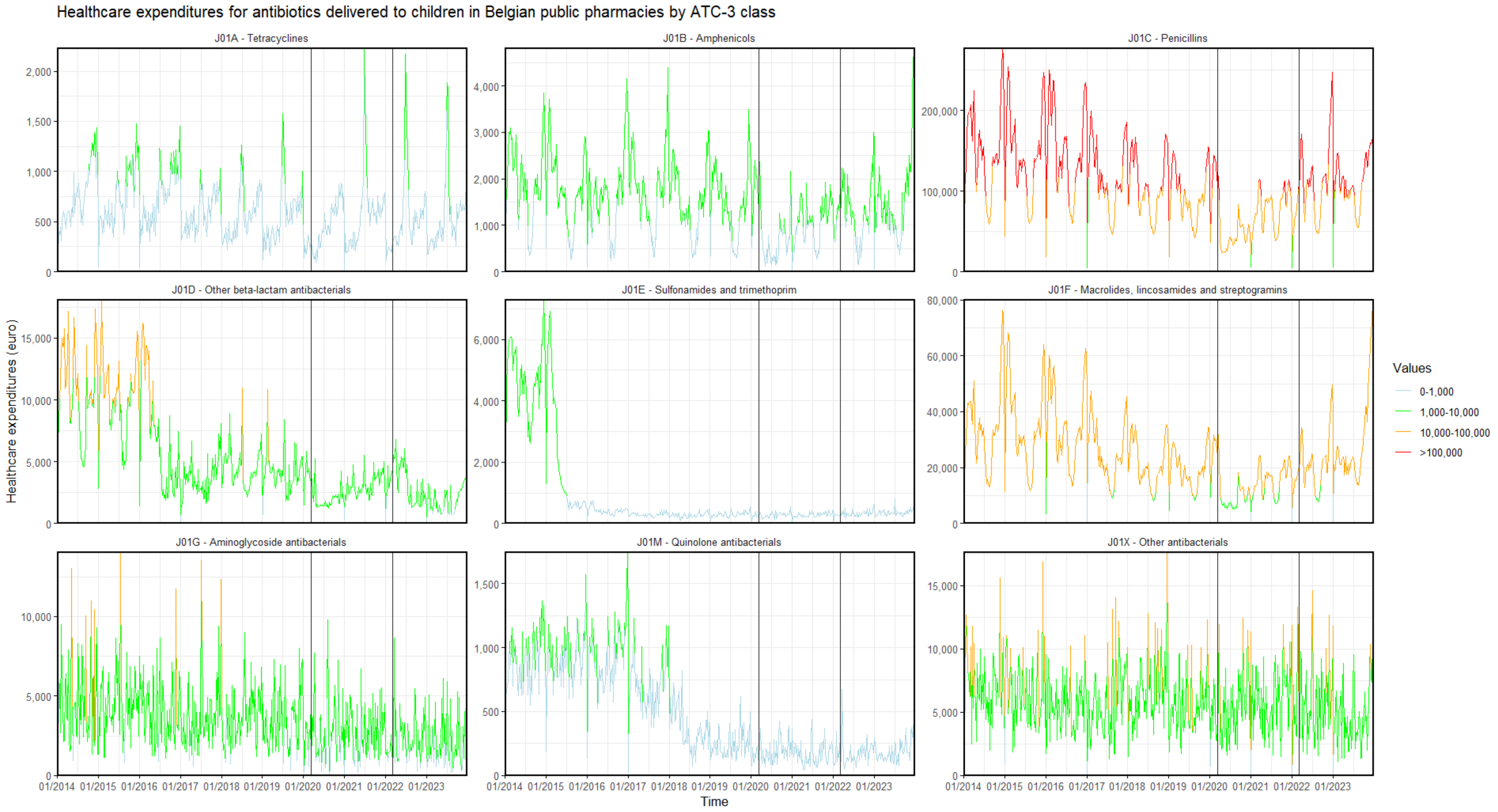
Healthcare expenditures for antibiotics delivered to children in Belgian public pharmacies by ATC-3 class
J01A - Tetracyclines
J01B - Amphenicols
J01C - Penicillins
J01D - Other beta-lactam antibacterials
J01E - Sulfonamides and trimethoprim
J01F - Macrolides, lincosamides and streptogramins
J01G - Aminoglycoside antibacterials
J01M - Quinolone antibacterials
J01X - Other antibacterials
Healthcare expenditures (euro)
Time
Values
0-1,000
1,000-10,000
10,000-100,000
>100,000

(c)

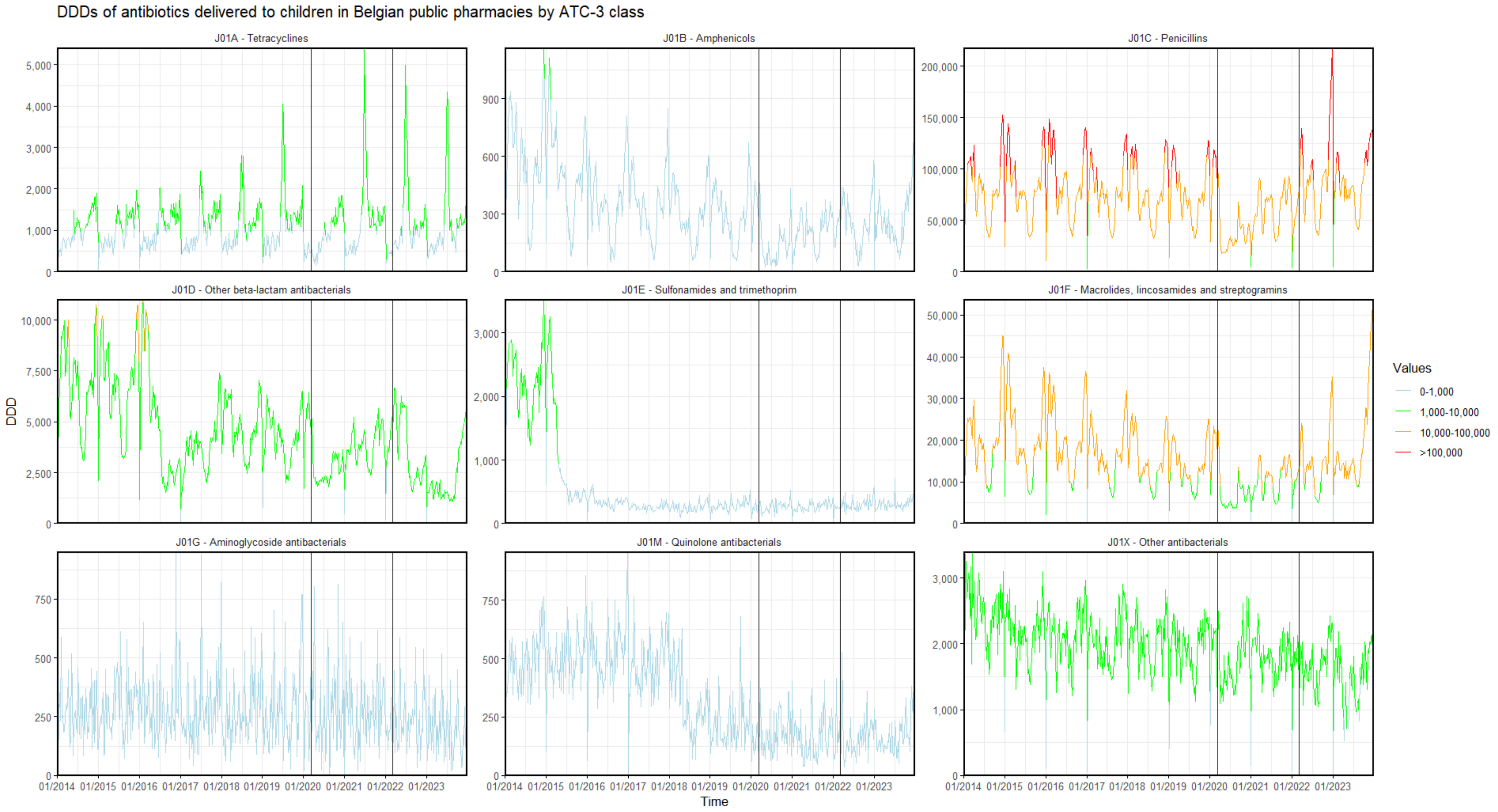

Figure S22. Line chart of antibiotics delivered to children in Belgian public pharmacies by ATC-3 class, expressed as number of packages (a), healthcare expenditures (b), and Defined Daily Doses (DDDs) (c).

ATC: Anatomical Therapeutic Chemical classification system.

The grey vertical lines represent the start of the COVID-19 pandemic (i.e., the week of 16 March 2020) and the start of the post-COVID period (i.e., the week of 14 March 2022), respectively.

(a)

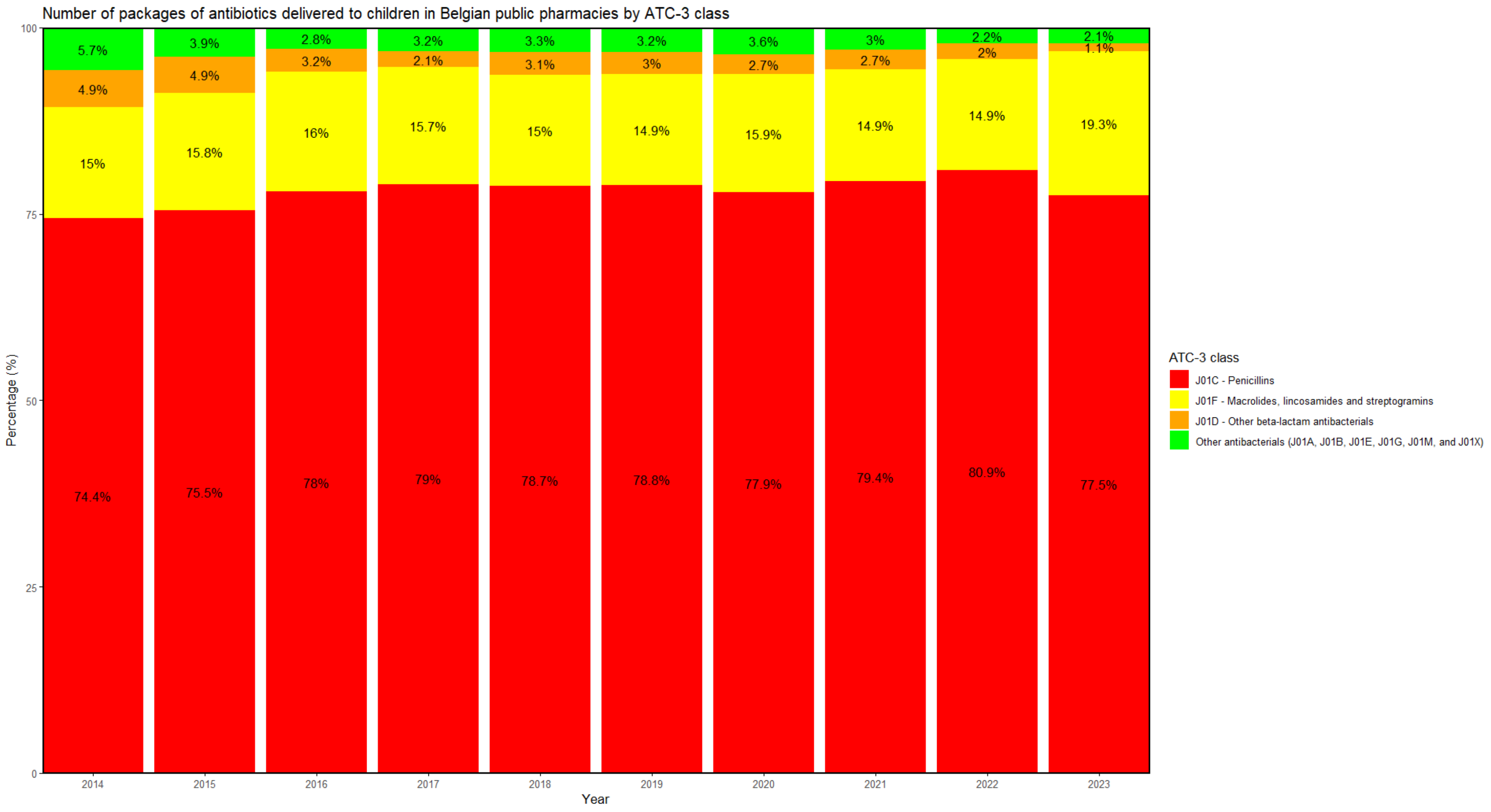

Number of packages of antibiotics delivered to children in Belgian public pharmacies by ATC-3 class
Percentage (%)
100
75
50
25
0
Year
2014
2015
2016
2017
2018
2019
2020
2021
2022
2023
5.7%
4.9%
15%
74.4%
3.9%
4.9%
15.8%
75.5%
2.8%
3.2%
16%
78%
3.2%
2.1%
15.7%
79%
3.3%
3.1%
15%
78.7%
3.2%
3%
14.9%
78.8%
3.6%
2.7%
15.9%
77.9%
3%
2.7%
14.9%
79.4%
2.2%
2%
14.9%
80.9%
2.1%
1.1%
19.3%
77.5%
ATC-3 class
J01C - Penicillins
J01F - Macrolides, lincosamides and streptogramins
J01D - Other beta-lactam antibacterials
Other antibacterials (J01A, J01B, J01E, J01G, J01M, and J01X)

(b)

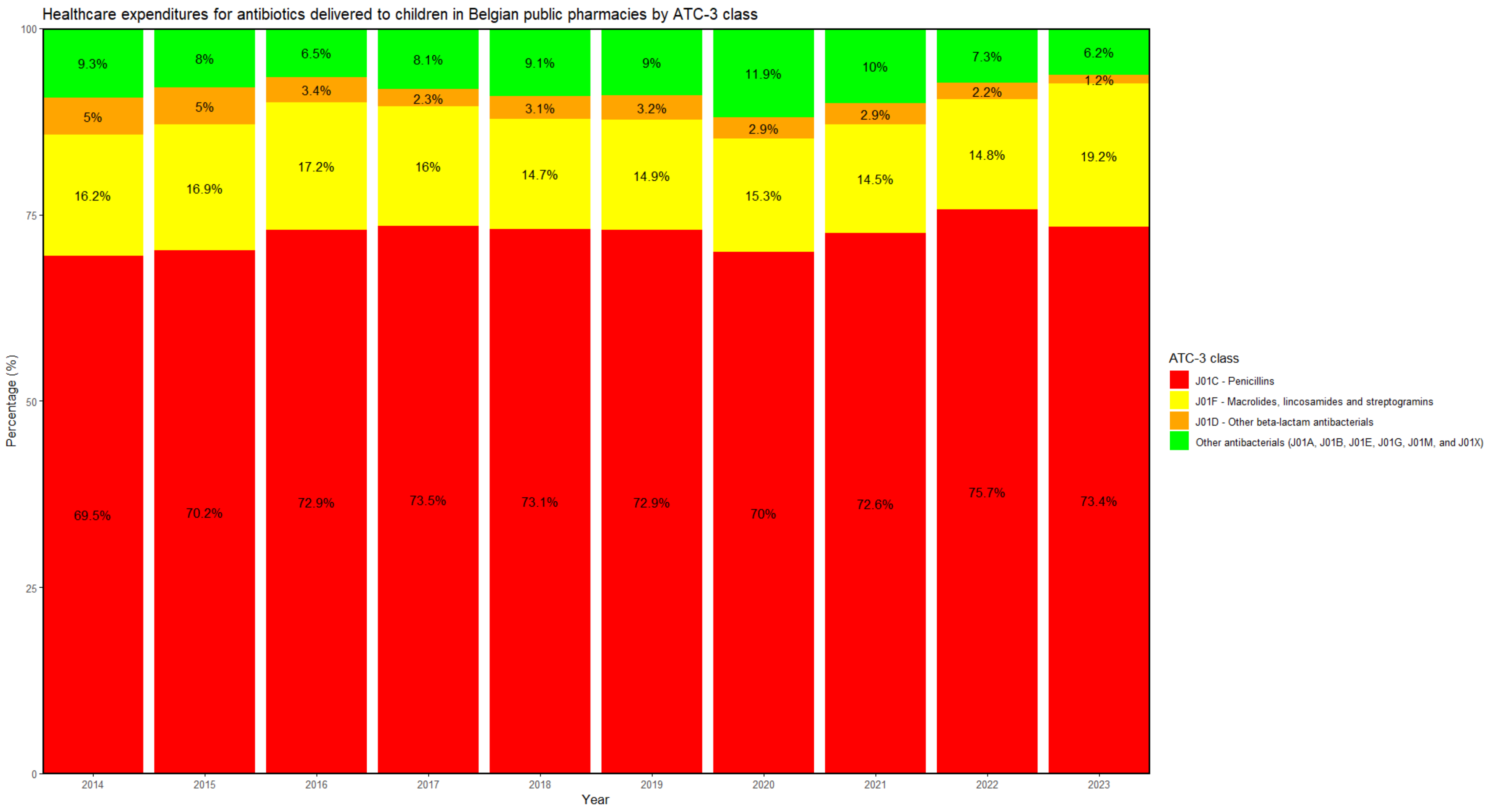
Healthcare expenditures for antibiotics delivered to children in Belgian public pharmacies by ATC-3 class
Percentage (%)
Year
2014
2015
2016
2017
2018
2019
2020
2021
2022
2023
0
25
50
75
100
9.3%
5%
16.2%
69.5%
8%
5%
16.9%
70.2%
6.5%
3.4%
17.2%
72.9%
8.1%
2.3%
16%
73.5%
9.1%
3.1%
14.7%
73.1%
9%
3.2%
14.9%
72.9%
11.9%
2.9%
15.3%
70%
10%
2.9%
14.5%
72.6%
7.3%
2.2%
14.8%
75.7%
6.2%
1.2%
19.2%
73.4%
ATC-3 class
J01C - Penicillins
J01F - Macrolides, lincosamides and streptogramins
J01D - Other beta-lactam antibacterials
Other antibacterials (J01A, J01B, J01E, J01G, J01M, and J01X)

(c)

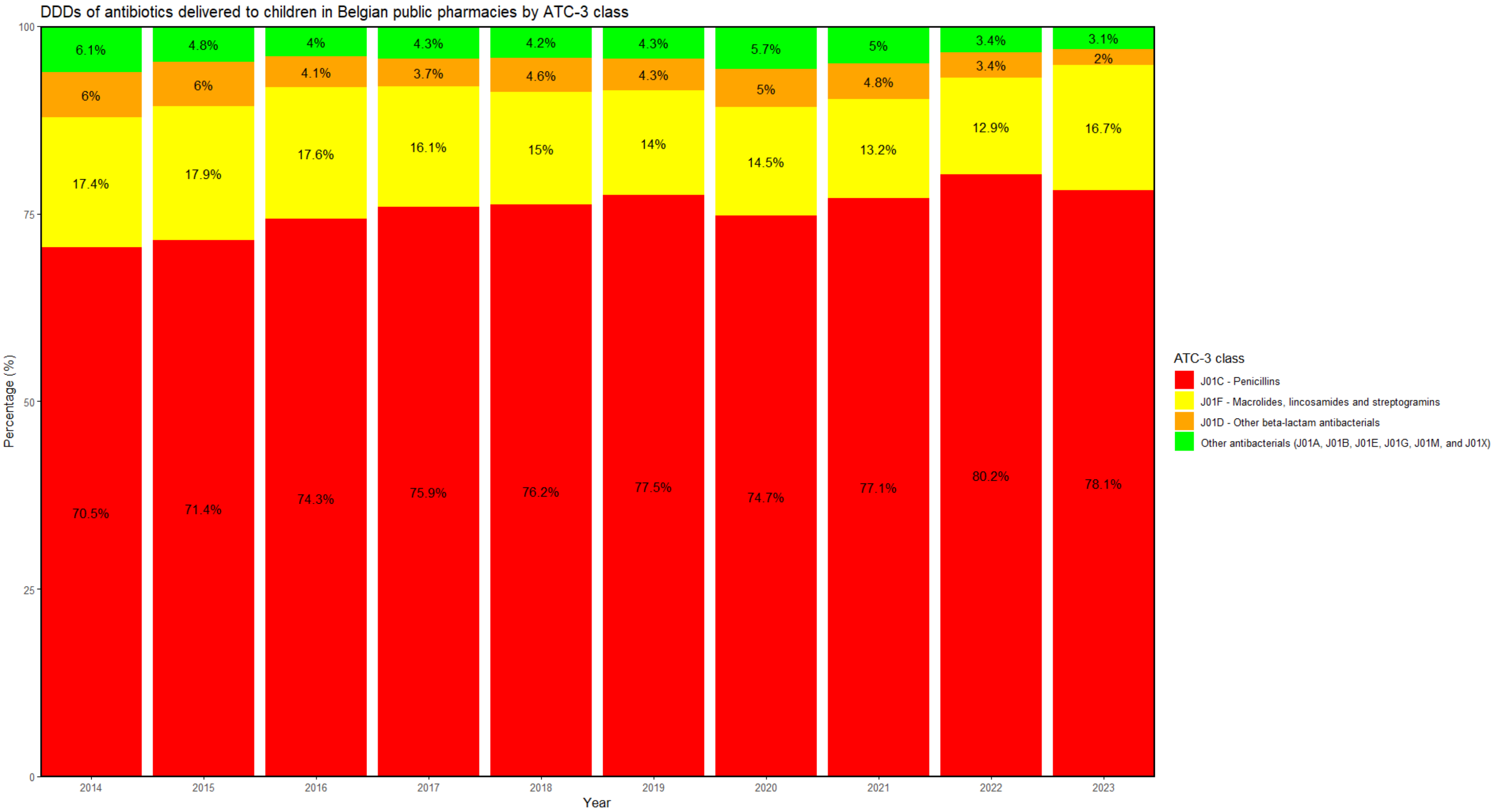

Figure S23. Bar chart of antibiotics delivered to children in Belgian public pharmacies by ATC-3 class, expressed as number of packages (a), healthcare expenditures (b), and Defined Daily Doses (DDDs) (c).

ATC: Anatomical Therapeutic Chemical classification system

(a)

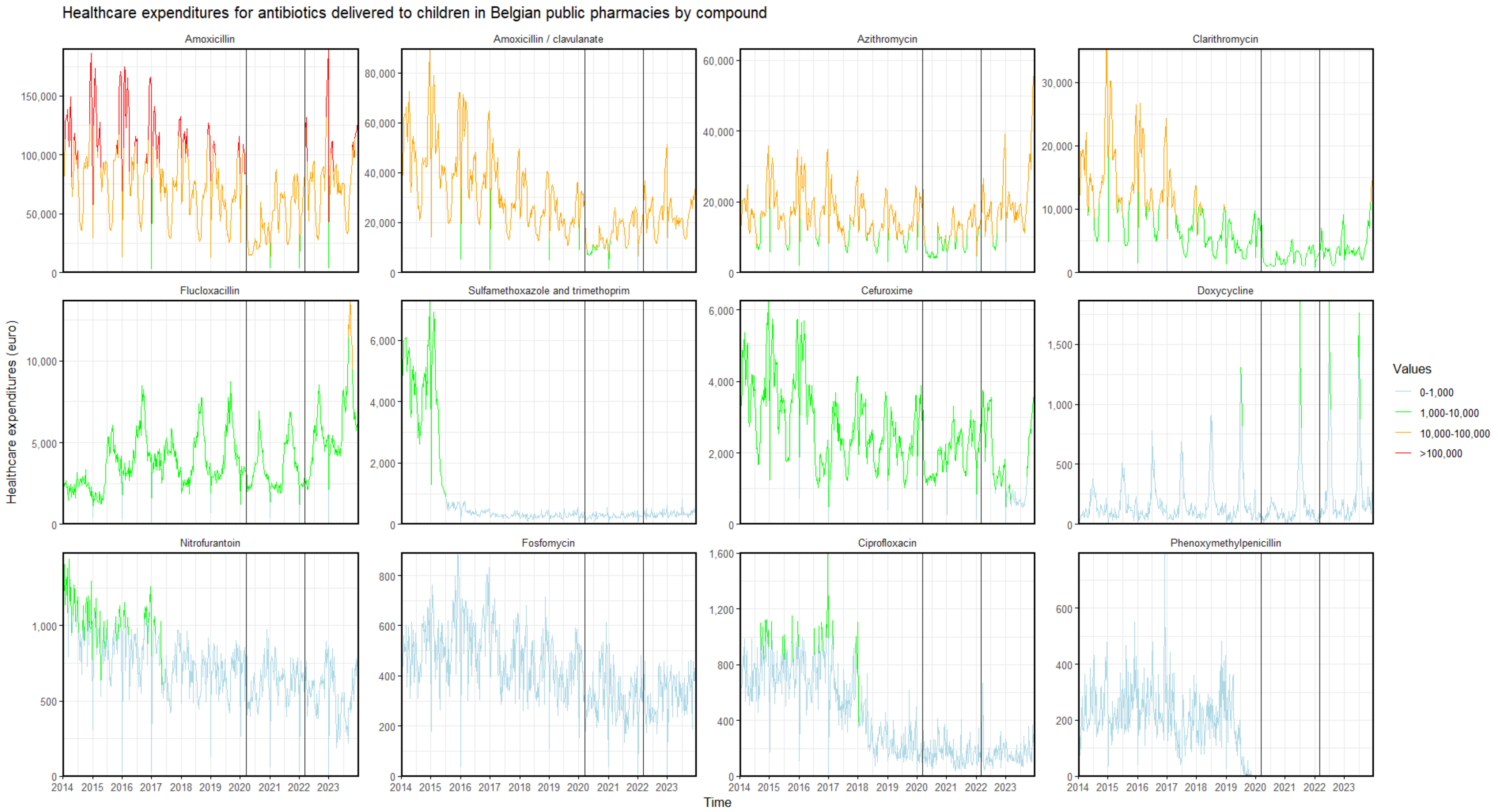
Healthcare expenditures for antibiotics delivered to children in Belgian public pharmacies by compound
Amoxicillin
Amoxicillin / clavulanate
Azithromycin
Clarithromycin
Flucloxacillin
Sulfamethoxazole and trimethoprim
Cefuroxime
Doxycycline
Nitrofurantoin
Fosfomycin
Ciprofloxacin
Phenoxymethylpenicillin
Healthcare expenditures (euro)
Time
Values
0-1,000
1,000-10,000
10,000-100,000
>100,000

(b)

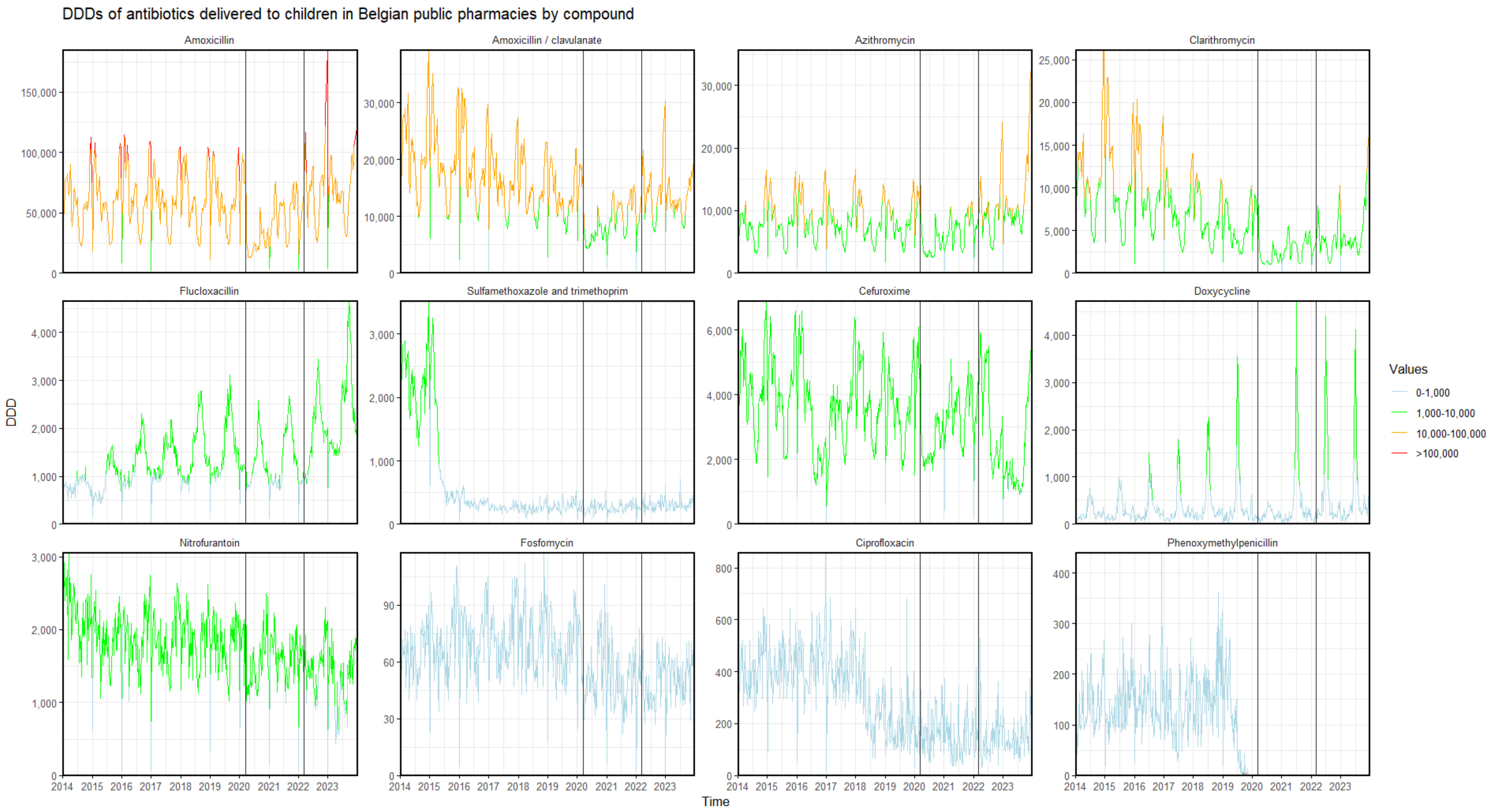

Figure S24. Line chart of antibiotics delivered to children in Belgian public pharmacies by compound, expressed as healthcare expenditures (a) and Defined Daily Doses (DDDs) (b).

The grey vertical lines represent the start of the COVID-19 pandemic (i.e., the week of 16 March 2020) and the start of the post-COVID period (i.e., the week of 14 March 2022), respectively.

(a)

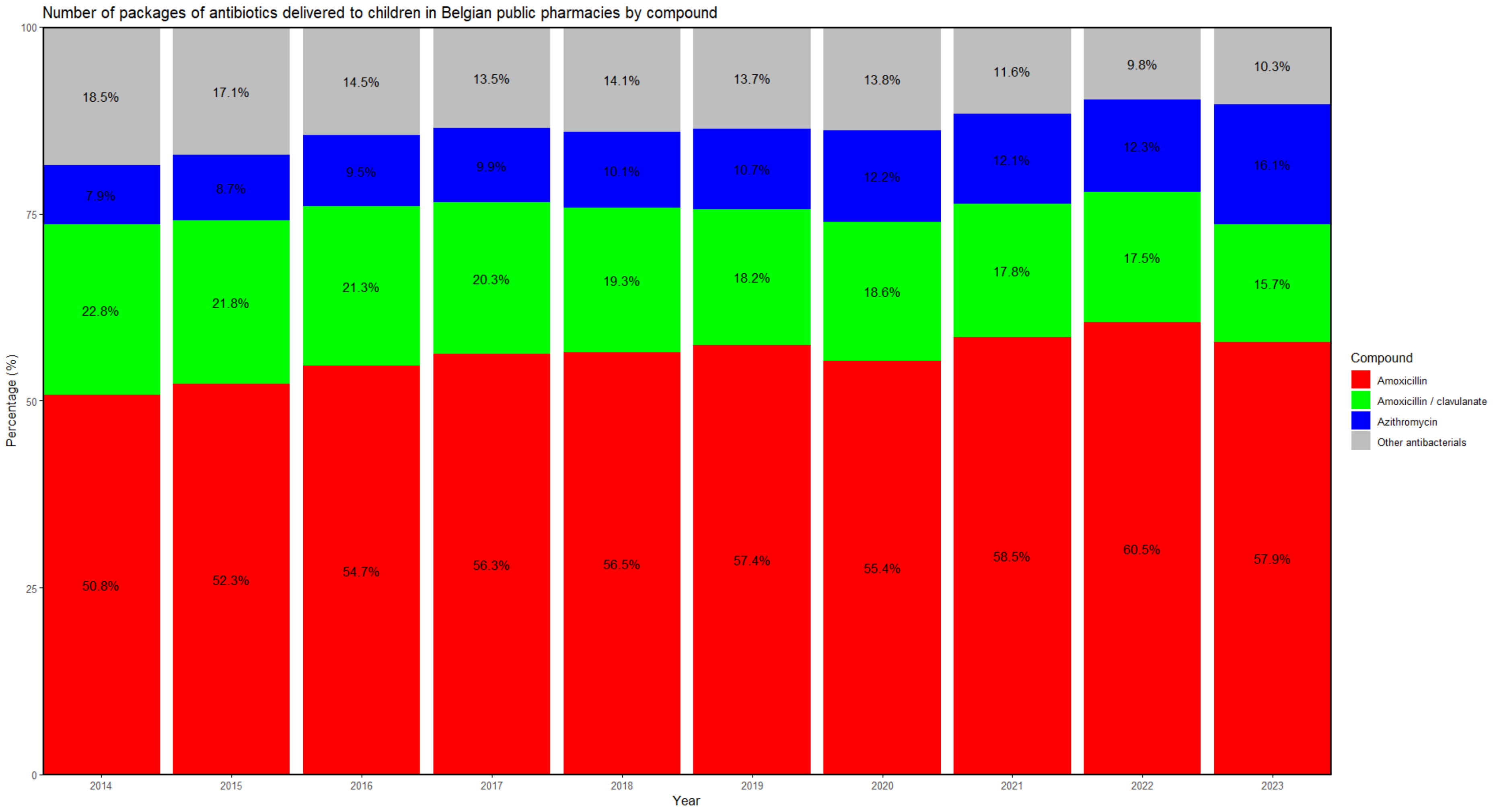
Number of packages of antibiotics delivered to children in Belgian public pharmacies by compound
Percentage (%)
Year
100
75
50
25
0
2014
2015
2016
2017
2018
2019
2020
2021
2022
2023
18.5%
17.1%
14.5%
13.5%
14.1%
13.7%
13.8%
11.6%
9.8%
10.3%
7.9%
8.7%
9.5%
9.9%
10.1%
10.7%
12.2%
12.1%
12.3%
16.1%
22.8%
21.8%
21.3%
20.3%
19.3%
18.2%
18.6%
17.8%
17.5%
15.7%
50.8%
52.3%
54.7%
56.3%
56.5%
57.4%
55.4%
58.5%
60.5%
57.9%
Compound
Amoxicillin
Amoxicillin / clavulanate
Azithromycin
Other antibacterials

(b)

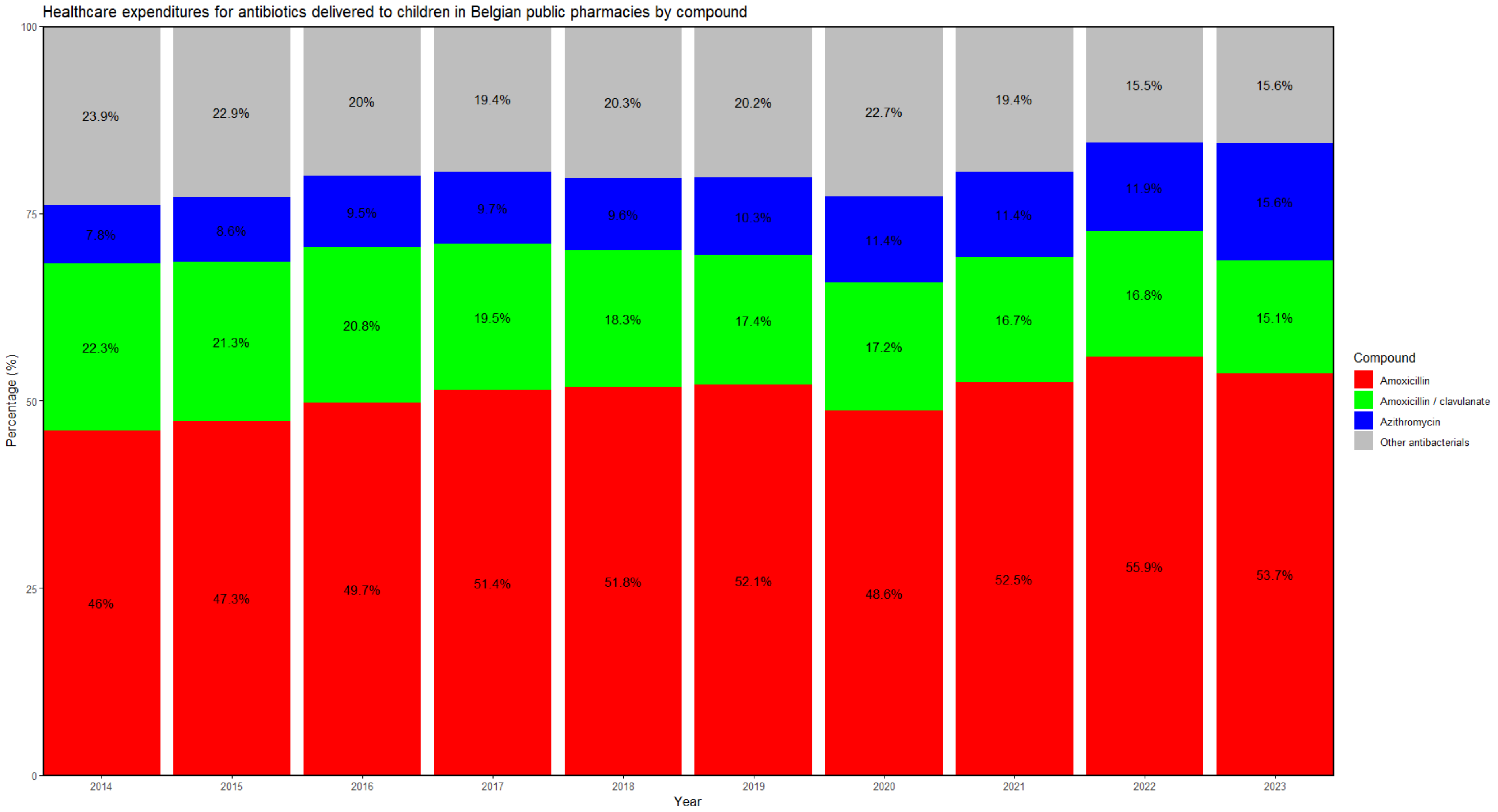

Healthcare expenditures for antibiotics delivered to children in Belgian public pharmacies by compound
Percentage (%)
Year
2014
2015
2016
2017
2018
2019
2020
2021
2022
2023
0
25
50
75
100
23.9%
22.9%
20%
19.4%
20.3%
20.2%
22.7%
19.4%
15.5%
15.6%
7.8%
8.6%
9.5%
9.7%
9.6%
10.3%
11.4%
11.4%
11.9%
15.6%
22.3%
21.3%
20.8%
19.5%
18.3%
17.4%
17.2%
16.7%
16.8%
15.1%
46%
47.3%
49.7%
51.4%
51.8%
52.1%
48.6%
52.5%
55.9%
53.7%
Compound
Amoxicillin
Amoxicillin / clavulanate
Azithromycin
Other antibacterials

(c)

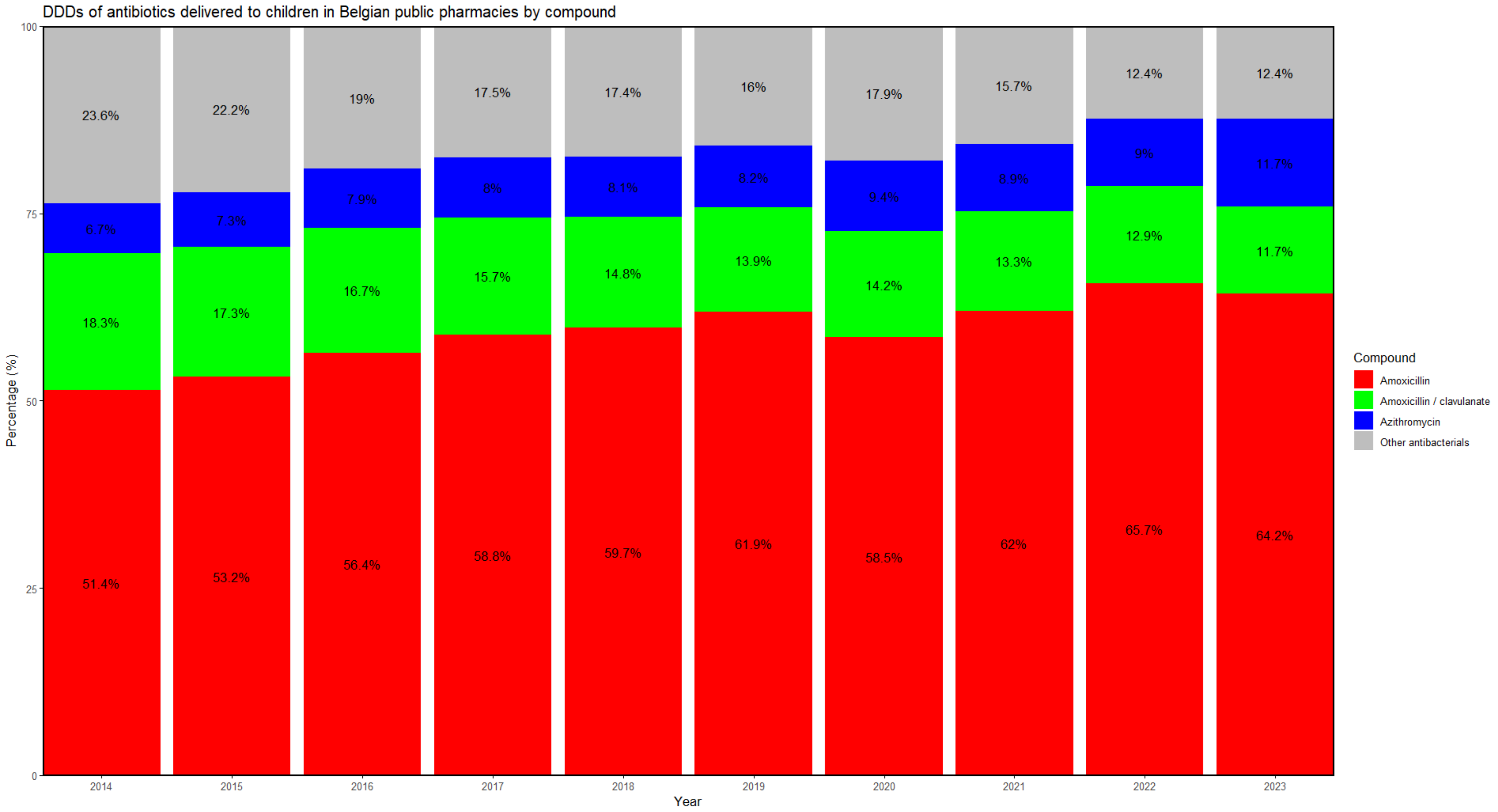

Figure S25. Bar chart of antibiotics delivered to children in Belgian public pharmacies by compound, expressed as number of packages (a), healthcare expenditures (b), and Defined Daily Doses (DDDs) (c).

(a)

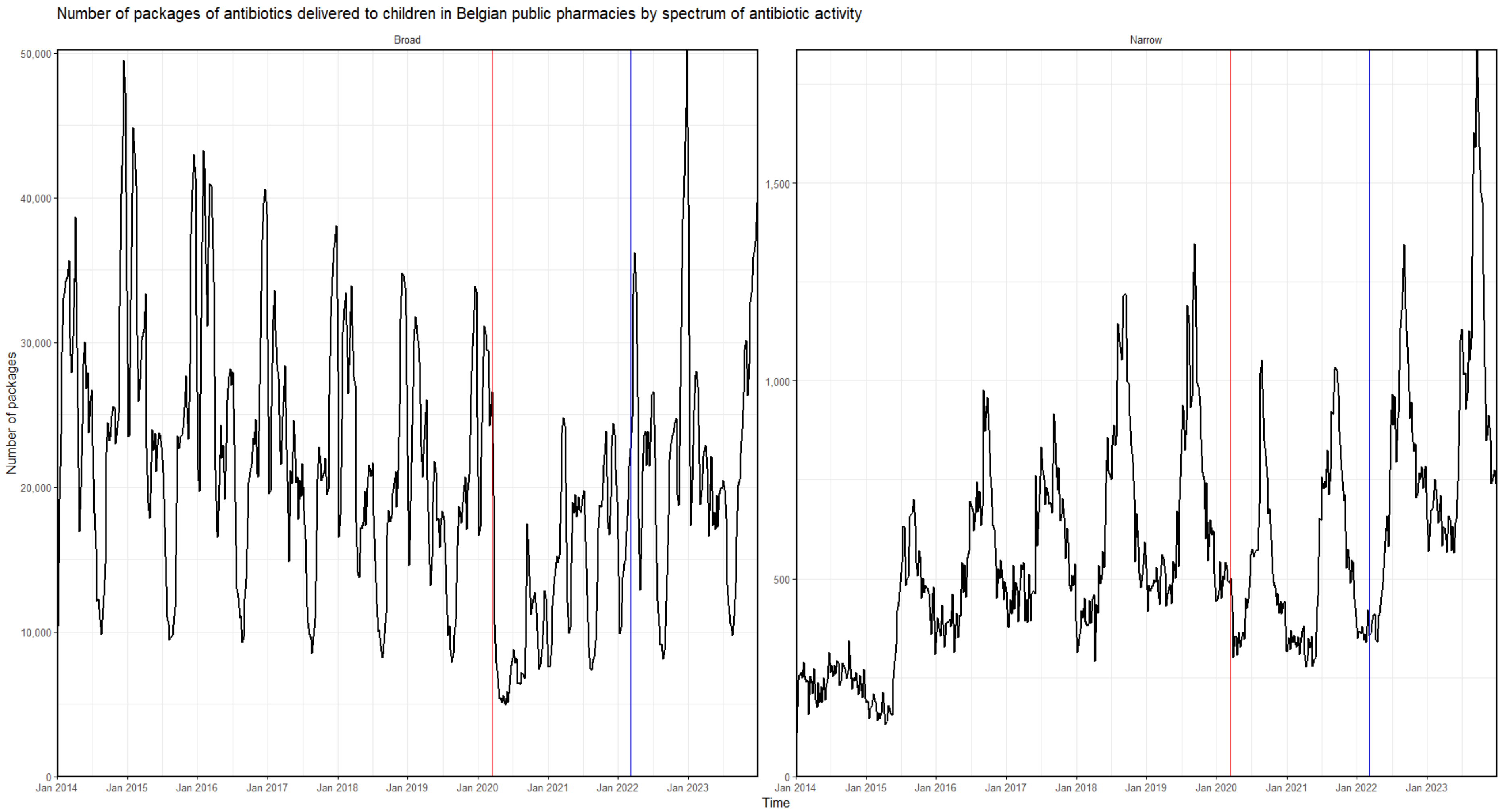
Number of packages of antibiotics delivered to children in Belgian public pharmacies by spectrum of antibiotic activity
Broad
Narrow
Number of packages
50,000
40,000
30,000
20,000
10,000
0
1,500
1,000
500
0
Jan 2014
Jan 2015
Jan 2016
Jan 2017
Jan 2018
Jan 2019
Jan 2020
Jan 2021
Jan 2022
Jan 2023
Time

(b)

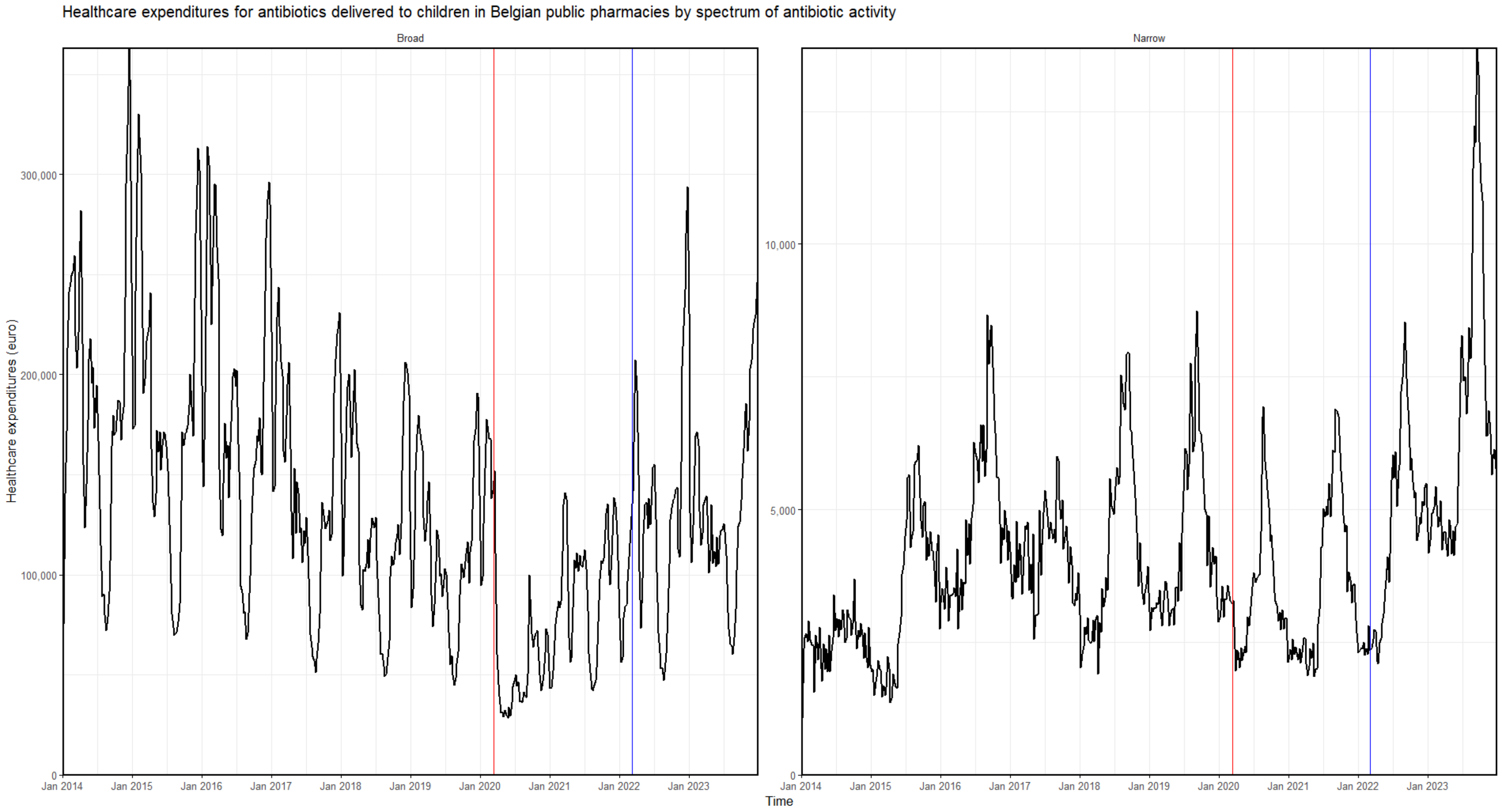
Healthcare expenditures for antibiotics delivered to children in Belgian public pharmacies by spectrum of antibiotic activity
Broad
Narrow
Healthcare expenditures (euro)
300,000
200,000
100,000
0
10,000
5,000
0
Jan 2014
Jan 2015
Jan 2016
Jan 2017
Jan 2018
Jan 2019
Jan 2020
Jan 2021
Jan 2022
Jan 2023
Time

(c)

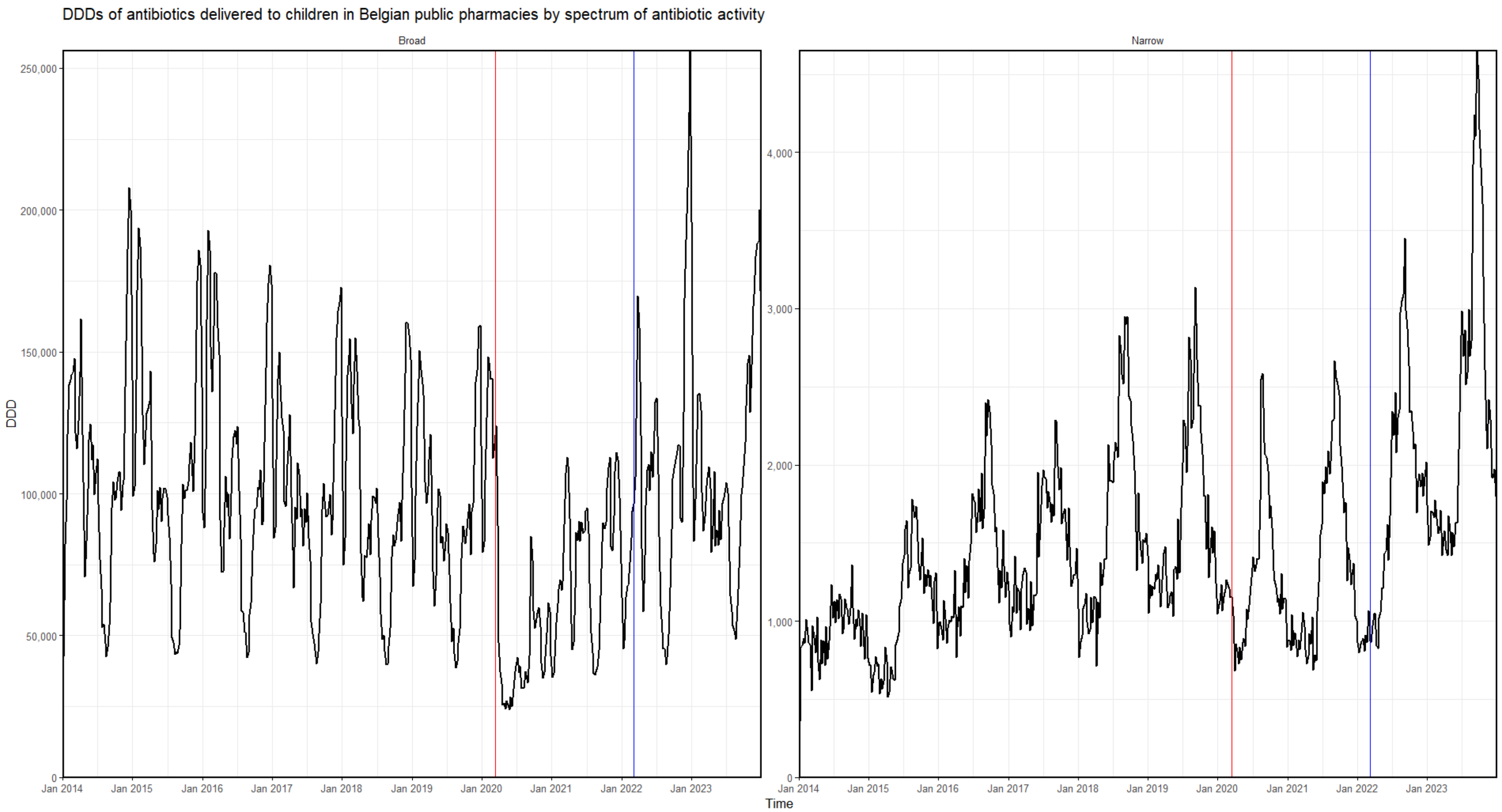

Figure S26. Line chart of antibiotics delivered to children in Belgian public pharmacies by spectrum of antibiotic activity, expressed as number of packages (a), healthcare expenditures (b), and Defined Daily Doses (DDDs) (c).
The red line represents the start of the COVID-19 pandemic (i.e., the week of 16 March 2020). The blue line represents the start of the post-COVID period (i.e., the week of 14 March 2022).
